# A comprehensive study on the impact of *Ligustrum vicaryi* L. fruit polysaccharide on myocardial fibrosis through animal experiments, network pharmacology and molecular docking

**DOI:** 10.3389/fcvm.2025.1470761

**Published:** 2025-02-20

**Authors:** Shuling Liu, Meifang Liu, Jianan Wang, Ruixue Rong, Yanwei Gao, Xiaoqi Li, Xin Liu, Shaojian Li

**Affiliations:** School of Pharmacy, Jining Medical University, Rizhao, China

**Keywords:** myocardial fibrosis, polysaccharide, network pharmacology, pathway enrichment analysis, isoproterenol

## Abstract

**Background:**

Myocardial fibrosis (MF) is a prevalent pathological condition associated with various heart diseases, such as heart failure and arrhythmias, which disrupt electrical signals and reduce pumping efficiency. This research explored the therapeutic effects and potential mechanisms of *Ligustrum vicaryi* L. fruit polysaccharide (LVFP) on MF.

**Methods:**

*In vivo* experiments, including fibrosis markers assay, echocardiography, HE staining, Sirius red staining, and Masson's trichrome staining, were performed to evaluate the therapeutic efficacy of LVFP in treating isoproterenol (ISO)-induced MF. We utilized the PharmMapper database to identify targets of LVFP, aiming to explore potential targets. Additionally, we obtained MF-related targets from the GeneCards database. We utilized Venny, a bioinformatics tool, to identify the intersection between the targets of LVFP and those related to MF. We utilized the STRING database to construct a protein interaction network for the overlapping targets and identified key targets for LVFP in treating MF through cytoHubba analysis. We conducted Gene Ontology (GO) and Kyoto Encyclopedia of Genes and Genomes (KEGG) enrichment analysis on the intersection targets. We also examined the interaction between LVFP and the key targets using molecular docking techniques.

**Results:**

LVFP significantly inhibited fibrosis biomarker such as hydroxyproline (HYP) and decreased myocardial fibrosis level as shown by heart weight to tibia length (HW/TL) measurement when compared to ISO-treated mice. Additionally, it increased ejection fraction (EF) and fractional shortening (FS) levels. LVFP showed decreased collagen levels compared to the ISO-treated mice by histological quantification of cardiac fibrosis. Based on the monosaccharide structures of LVFP, 413 targets were identified, with 67 associated with MF. Analysis indicated that the 9 hub genes (AKT1, HSP90AA1, SRC, GSK3β, VEGFR2, RHOA, ENO1, PKM, and IL-2) play roles in MF treatment by participating in signaling pathways related to prostate cancer, lipid and atherosclerosis, and insulin resistance. Molecular docking results showed that LVFP exhibited strong binding potential to VEGFR2 (−8.65 kcal/mol), AKT1 (−7.36 kcal/mol) and GSK3β (−7.68 kcal/mol).

**Conclusion:**

LVFP shows promise as a therapeutic agent for MF, primarily through the regulation of various signaling pathways and targets. These findings provide novel insights for the treatment of MF utilizing LVFP.

## 1 Introduction

Myocardial fibrosis (MF), a common feature in various cardiomyopathies (1), is marked by excessive extracellular matrix (ECM) protein deposition, leading to structural changes and functional impairment of the myocardium. MF contributes to ventricular remodeling and heart failure, commonly occurring in conditions like myocardial infarction, hypertrophy, and dilated cardiomyopathy. Despite its clinical significance, the molecular mechanisms underlying MF remain elusive, and effective therapeutic interventions are still lacking (2). Consequently, the treatment strategies for MF remain a critical concern in the clinical setting (3).

A hybrid of *Ligustrum vulgale* L. and *Ligustrum ovalifolium Hassk. var. aureo-marginatum*, *Ligustrum vicaryi* L. belongs to the Oleaceae family. Our previous study revealed that *Ligustrum vicaryi* L. fruit polysaccharide (LVFP) exhibits immunomodulatory, antioxidant, and anti-inflammatory activities in cyclophosphamide-induced immunosuppressed mice (4). In addition, fibrosis represents a crucial pathological mechanism involved in various chronic inflammatory conditions (5). Polysaccharides derived from plants have been found to enhance cardiac function by increasing antioxidant enzyme activity in both blood and heart (6). Recently, there has been growing interest in natural compounds, especially polysaccharides, for their potential in combating MF. Several polysaccharides such as a*stragalus* polysaccharides, *Lycium barbarum* polysaccharides, and *Polygonatum sibiricum* polysaccharides have shown effects on MF. A*stragalus* polysaccharides not only prevent ISO-induced MF (7), but also slow down MF diabetic cardiomyopathy db/db mice by regulating Bcl-2, Caspase-3 and Bax protein levels (8). Additionally*, Lycium barbarum* polysaccharides alleviate MF in rats with chronic heart failure via the TGF-β1/Smad3 signaling pathway (9). Moreover, *Polygonatum sibiricum* polysaccharides reduce collagen buildup in heart tissue, enhance hemodynamics, and prevent MF in diabetic rats by increasing BMP-7 and Smad7 expression and decreasing Smad3 expression (10). Based on the aforementioned conditions, we hypothesized that LVFP possesses anti-myocardial fibrosis properties.

**Figure 1:**
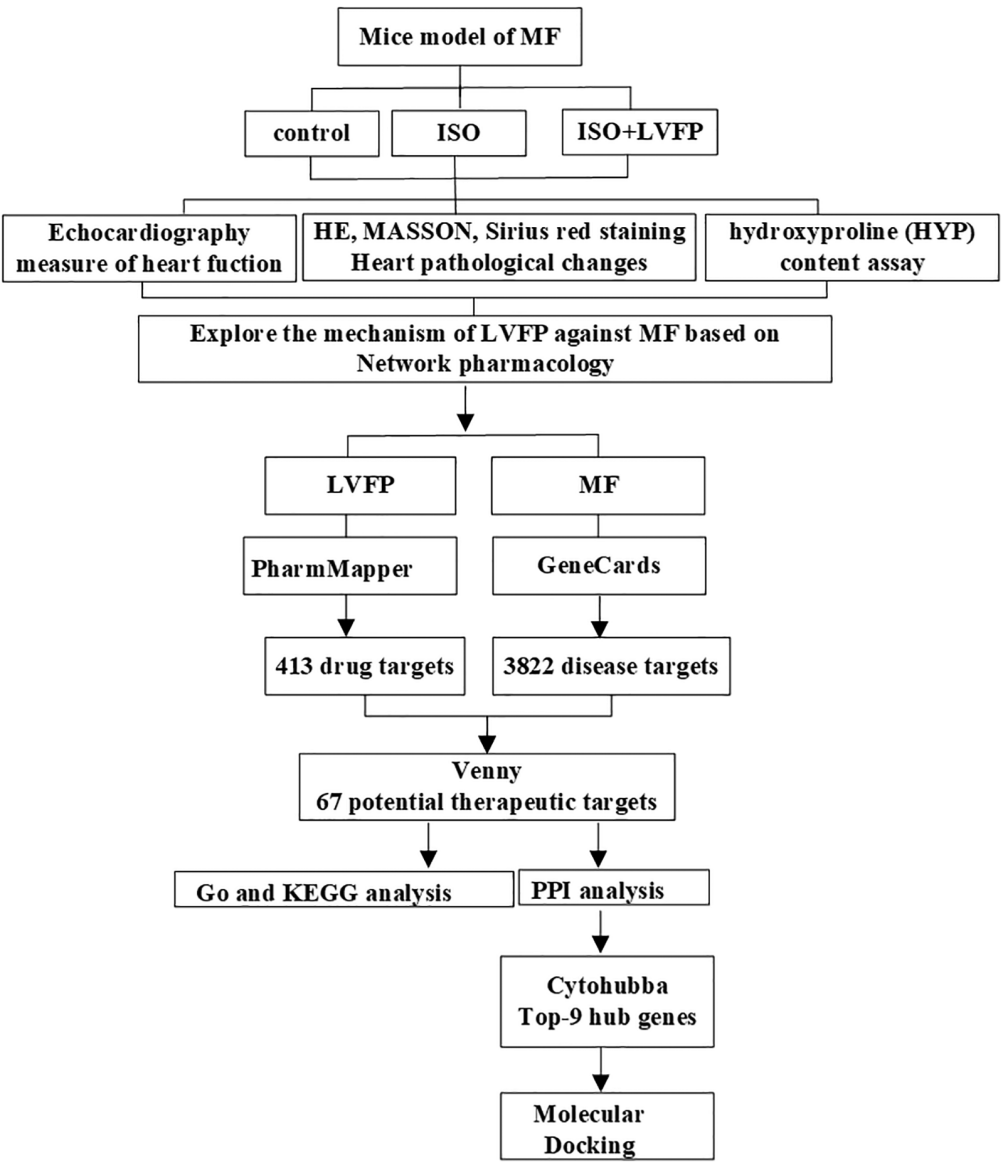
The overall framework diagram depicts how LVFP targets key biological functions and mechanisms in the treatment of MF, utilizing insights from experimental evaluations, network pharmacology, and molecular docking.

Network pharmacology is a holistic approach that explores biological systems through their networks (11). It is widely used in research on Chinese herbal medicine to investigate its various ingredients (12). In network pharmacology, the network targets and multi-components concept is the optimal method for studying the molecular therapeutic effects of herbal medicine. This concept is a valuable tool that allows researchers to explore how herbal medicine works at the molecular level (13). In addition, molecular docking is a computational approach that helps to predict how small molecules interact with larger macromolecular targets (14). Molecular docking tests indicated that these compounds bind closely to their predicted target proteins (13). In this study, we integrated with *in vivo* experiments, network pharmacology and molecular docking to uncover LVFP treats MF. Figure 1 illustrates the complete scheme of this study.

## 2 Materials and methods

### 2.1 Materials and reagents

ISO (isoproterenol), from Sigma-Aldrich (St. Louis, MO, USA), was dissolved in phosphate-buffered saline. Hydroxyproline (HYP) assay kit (Lotion: A030-2-1) was obtained from Jiancheng Bioengineering Institute (Nanjing, China). Isoflurane (Lotion: 2023032002) was gained from RWD life science (Shenzhen, China). Hematoxylin and eosin staining (Lotion: G1001), Improved Sirius red staining kit (Lotion: G1006), and Masson Tri-Color dyeing solution (Lotion: G1078) were obtained from Servicebio Technology Co., Ltd (Wuhan, China).

### 2.2 Animal model and drug treatment

The Jining Medical University Animal Care and Use Ethics Committee approved the research under authorization number JNMC-2021-DW-007. Male C57BL/6J mice, aged 8–10 weeks, were obtained from Jinan Pengyue Laboratory Animal Breeding Co., Ltd., Jinan, China. The mice were individually housed in hygienic cages with a 12/12 h light dark cycle, a temperature of 22 ± 3°C, and a humidity of 45% ± 10% for a 7-day pre-adaptation period before the experiments. The mice had unrestricted access to a regular diet and tap water. Subsequently, the mice were at random divided into three treatment groups, namely Control, ISO, and ISO + LVFP with 10 mice per group. In the ISO model group, ISO was administered subcutaneously at 5 mg/kg/day for one day, then reduced to 2.5 mg/kg/day for the next 13 days (15, 16). Based on our previous research, LVFP (400 mg/kg/day) was found to be effective and safe in the treatment groups, LVFP was administered intraperitoneally for 14 days. The control group received the same dosage of LVFP solution.

### 2.3 Echocardiography

Echocardiography was performed to measure heart function. After being anesthetized with 1.5% isoflurane, the mice underwent echocardiography using the Vevo 3100 Imaging System (RRID: SCR_022152) from Visual Sonics Inc., based in Toronto, Canada. B-mode parasternal short-axis scans were performed by positioning the probe to measure the maximum length of the left ventricle. M-mode images were captured by aligning the cursor perpendicular to the maximum LV dimension during end-diastole and systole, facilitating the measurement of wall thickness and chamber dimensions pre- and post-treatment with periplocymarin (17). Metrics such as left ventricular ejection fraction (LVEF), left ventricular fractional shortening (LVFS), and fractional area change (FAC) were assessed using Vevo LAB 5.6.0 software.

### 2.4 Biochemical markers assay and histological quantification of MF

After 14-day's treatment, all mice were weighed and blood was collected from eyeball. When sacrificed, mice were anesthetized by 5% isoflurane inhaled and decapitated. Body weight, heart weight and tibia length were measured for each mouse, and serum was separated from the blood to assess hydroxyproline (HYP) content. Mouse hearts were stored in neutral formalin for HE staining, Sirius Red staining, and Masson's trichrome staining. Following standard procedures, we performed HE staining, Sirius Red staining, and Masson's trichrome staining.

### 2.5 Prediction of potential targets of LVFP during MF treatment

Previous analyses utilizing fourier transform infrared spectroscopy, high-performance ion chromatography, and high-performance gel filtration chromatography revealed that LVFP is comprised of arabinose, rhamnose, galactose and glucose in a ratio of 7.55: 1.79:1.58:1.54, with a molecular weight of 88,949 Da (4). We investigated the chemical structure, drug likeness, and oral bioavailability of these monosaccharides in the TCMSP^1^ (RRID: SCR_023757) to predict the targets of LVFP. Targets were selected based on two criteria: drug similarity greater than 0.18 and oral bioavailability exceeding 30%. We gained a list of target genes by querying GeneCards^2^ (RRID: SCR_002773) using “myocardial fibrosis” as keywords, and further queried the gene names in the UniProt^3^ (RRID: SCR_002380). The overlap between disease genes and drug target genes were visualized using Venny diagram on Xiantao^4^ website to identify potential target genes for LVFP treats MF. Venn statistical analysis and visualization were conducted in R version 4.2.1 using the ggplot2 package [3.3.6]. The Venn Diagram analysis examined specific and common elements between data groups, and results were visualized with the ggplot2 and Venn Diagram packages.

### 2.6 Construction of a protein-protein interaction (PPI) network and identification of the hub genes

The intersection gene was entered into the search box named “Multiple proteins” in the STRING^5^ (version 12.0, RRID: SCR_005223) online database, which is an online database for exploring known protein interactions. The lowest interaction score in the final network was set to 0.400, and the primary network was concealed, showing only the essential target genes. A barplot was utilized to tally the connections for each node in the network, and the findings were saved in a TSV file. The TSV file was then imported into Cytoscape V.3.10.1 (RRID: SCR_003032) software to identify the hub genes, which may serve as potential targets for LVFP treats MF.

### 2.7 Gene ontology (Go) and kyto encyclopedia of gene and genomes (KEGG) enrichment analyses

We performed enrichment analyses on the intersecting genes to investigate their biological roles and pathways related to LVFP treats MF. GO/KEGG (RRID: SCR_012773) pathway analyses were processed using Metascape and the “cluster Profiler Package” [4.4.4] in R software (version 4.2.1), accessed through the Xiantao website^6^ according to the website tutorial. For GO and KEGG analyses, all analyses and visualizations were performed in R version 4.2.1 using the ggplot2 package. The enrichment analyses results were visualized with ggplot2.

### 2.8 Molecular docking

To elucidate the relationships between the hub genes and LVFP monosaccharide elements, the crystal structures of the key target proteins were sourced from research Collaboratory for Structural Bioinformatics Protein Data Bank^7^ (RCSB PDB, RRID: SCR_012820) (18). PyMOL (RRID: SCR_000305) and AutoDock (RRID: SCR_012746) 1.5.7 software were used to perform several preparatory steps, including the removal of water molecules, isolation of the original ligand from the receptor, hydrogenation, repair of broken chains, and calculation of protein charge (19). The structures of the core active compounds were saved in the Mol 2 format and converted to PDBQT format using Open Babel GUI software (RRID: SCR_014920). Molecular docking was performed using AutoDock1.5.7, and the results were analyzed using PyMOL. A binding energy <−5.0 kcal/mol was considered indicative of a strong docking affinity (20). Docking parameters that met both the binding energy criteria and formed hydrogen bonds were selected for further analysis.

### 2.9 Statistical analysis

Data are presented as the mean ± standard deviation (SD). We conducted normality tests (Shapiro–Wilk test) and variance homogeneity tests (Brown-Forsythe test and Bartlett's test) before performing the one-way ANOVA. The data met the assumptions of normality and homogeneity, justifying the use of one-way ANOVA for our statistical analysis. Group differences were evaluated using one-way ANOVA and subsequent *post-hoc* tests in GraphPad Prism (RRID: SCR_002798). A *p*-value of less than 0.05 was considered to indicate statistical significance.

## 3 Results

### 3.1 LVFP attenuated ISO -induced myocardial fibrosis

**Figure 2:**
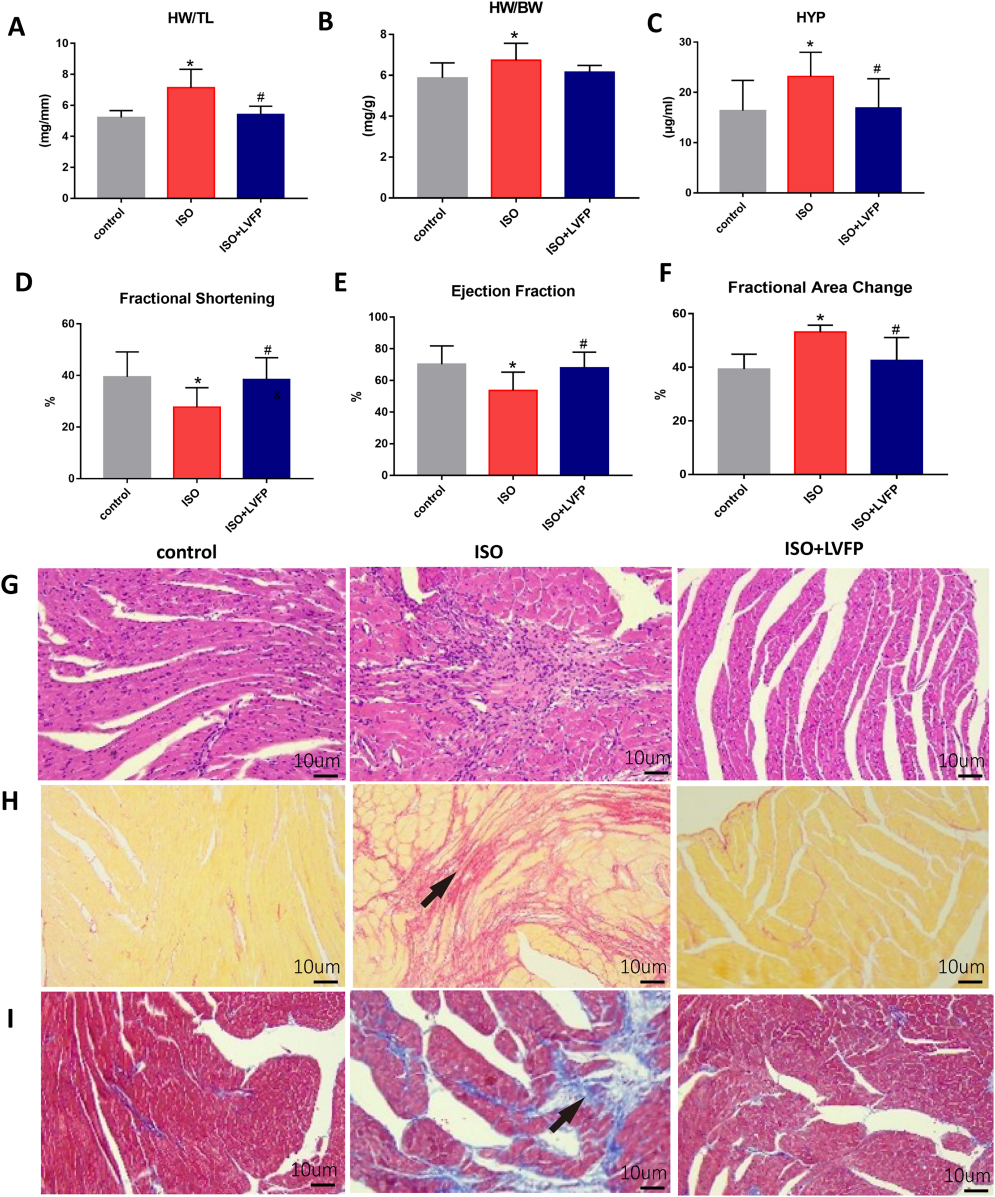
LVFP-mediated inhibitory effect on ISO-induced cardiac fibrosis. **(A)** heart weight to tibia length ratio (HW/TL). **(B)** Heart weight/body weight ratio (HW/BW) **(C)** Hydroxyproline level in serum (HYP). Data are mean ± S.E.M. *n* = 8. *P < 0.05 vs. control; ^#^*P* < 0.05, vs. ISO. Echocardiograph showing the effect of LVFP in attenuating myocardial fibrosis 14 days after ISO treatment. Supplementary Figure S1 Echo image with a resolution of 600 dpi are end-diastolic frames from the short-axis view. **(D)** Left ventricular ejection fraction (LVEF), **(E)** Left ventricular Fractional shorting (LVFS) and **(F)** Fractional area change (FAC) is shown for C57/BL6J mice. Values are expressed as the mea*n* ± standard deviation (*n* = 6). *p < 0.05. Representative micrographs (10× objective) of **(G)** HE staining of cardiac sections, **(H)** Sirius red staining for collagen deposition, indicated by red-stained ECM; and **(I)** Masson's trichrome staining for collagen deposition, indicated by blue-stained ECM. The scale bar is set at 10 μm. The arrows indicate an increase in myocardial fiber density.

We investigated how LVFP protects against ISO-induced myocardial fibrosis (MF) by measuring the weight-related parameters and examining the pathological changes in mice. The heart weight index, calculated by the heart weight to body weight (HW/BW) ratio, indicates cardiac hypertrophy (21). Moreover, myocardial fibrosis is key feature of cardiac hypertrophy (22). After 2 weeks of ISO administration, severe myocardial hypertrophy was observed in the ISO-treated mice, as evidenced by an increase in HW/TL and HW/BW ratios. LVFP treatment resulted in a significant reduction in the extent of myocardial hypertrophy compared to the ISO-treated mice, as indicated by HW/TL measurement (Figure 2A). In contrast, no significant difference in HW/BW was found between LVFP-treated and ISO-treated mice, indicating that pathological changes have not yet been detected (Figure 2B). Hydroxyproline, a crucial amino acid in collagen, acts as a biochemical marker for fibrosis (23). Administering LVFP resulted in reduced HYP concentrations (Figure 2C), suggesting that LVFP exerted a protective effect against MF. We performed echocardiographic measurements 14 days after administration of isoproterenol and LVFP to evaluate myocardial function. There were no significant differences in heart rate among the groups during anesthesia. LVFP treatment (400 mg/kg/day) significantly enhanced echocardiography parameters, including fractional shortening (FS) and ejection fraction (EF), while reducing the fractional area change (FAC) (Figures 2D–F; Supplementary Figure S1). We performed histological examinations using hematoxylin and eosin (H&E), Sirius red staining, and Masson's trichrome staining to thoroughly evaluate the degree of myocardial fibrosis. Figures 2G–I illustrates that ISO-treated mice exhibited increased collagen levels in the myocardium, while those receiving ISO + LVFP showed decreased collagen levels compared to the ISO-treated mice. Collectively, these findings demonstrate that LVFP treatment prevents ISO-induced MF and preserves cardiac function.

### 3.2 Properties and chemical structures of LVFP monosaccharides

Supplementary Table S1 presents the pharmacological properties and chemical structures of the four monosaccharides. The log *P* values of the four monosaccharides of LVFP were negative, suggesting good suitability and water solubility for oral administration. The oral bioavailability of the monosaccharides was greater than or close to 30%, indicating their ease of absorption by the body. Galactose exhibited a drug-likeness score exceeding 0.18, suggesting its viability as a pharmaceutical candidate.

### 3.3 Targets of LVFP monosaccharides

**Figure 3:**
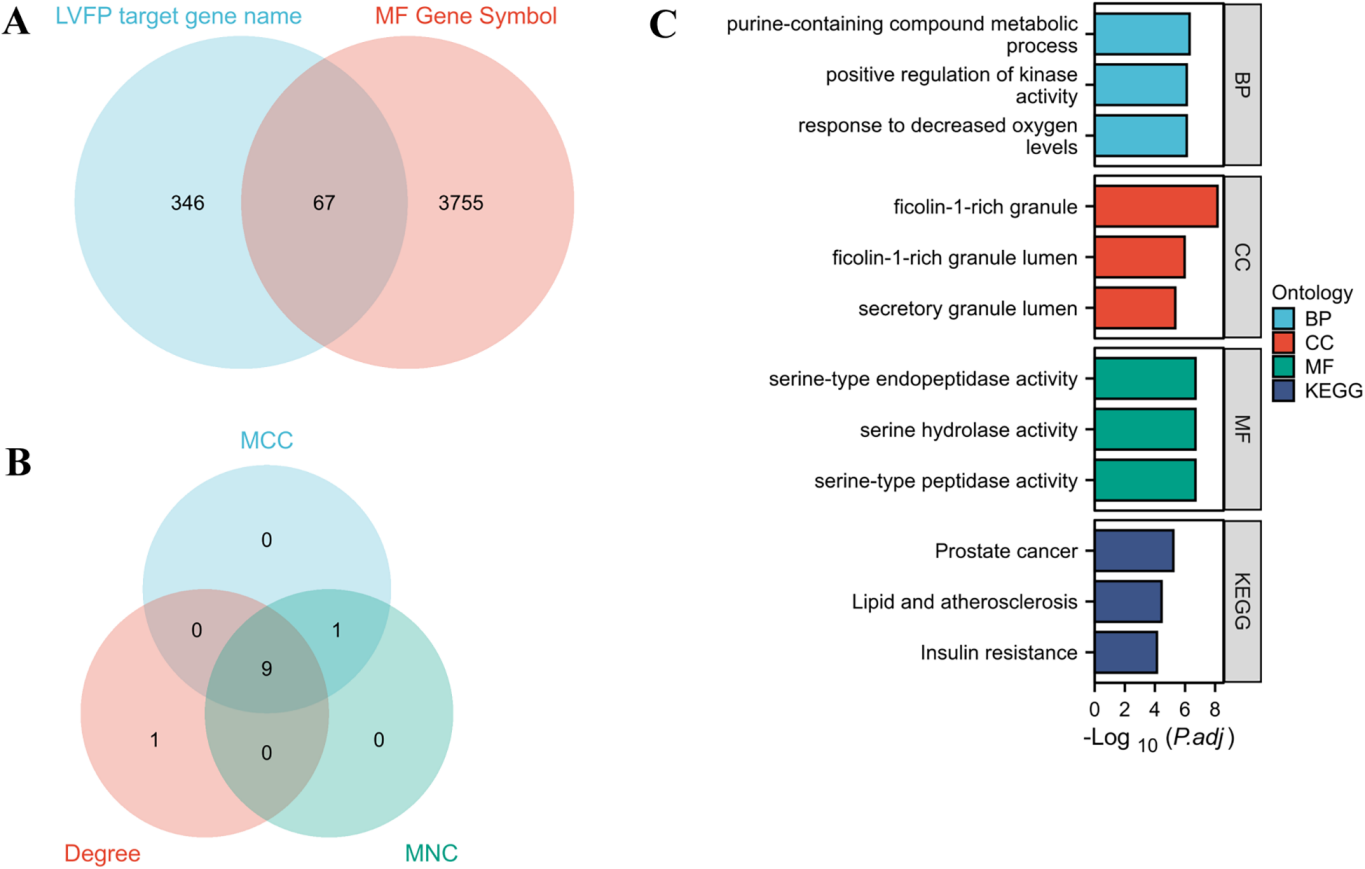
Target screening involved in LVFP treats MF. **(A)** Venn diagram of potential targets of LVFP treats myocardial fibrosis; **(B)** A venn diagram of hub genes by MCC, MNC, and degree analysis module; **(C)** the significant GO and KEGG pathways enriched by the intersected genes using Xiantao Website.

The potential targets of the monosaccharides were assembled from the TCMSP and PharmMapper databases. As a result, a total of 413 genes (Supplementary Table 2) were obtained from the PharmMapper prediction for LVFP target genes, while 67 genes were obtained from the intersection of MF and LVFP target genes (Figure 3A; Supplementary Table S2).

### 3.4 Construction of the LVFP hub genes for myocardial fibrosis

We constructed a protein-protein interaction (PPI) network of 67 intersected genes via the STRING database for *Homo sapiens* with a minimum interaction score of 0.9 the protein interaction map (Supplementary Figure S2) was saved as a TSV file and imported into Cytoscape software. 9 genes (AKT1, HSP90AA1, SRC, GSK3B, KDR, RHOA, ENO1, PKM, and IL-2) were identified as hub genes using the PPI network analysis and cytoHubba (RRID: SCR_017677) plugin (Figure 3B).

### 3.5 Enrichment analyses

We conducted biological functions and pathway enrichment analyses of 67 intersected genes for Gene Ontology (GO) and Kyoto Encyclopedia of Genes and Genomes (KEGG) via the Xiantao platform (24) (Supplementary Table S2). As a result, the KEGG enrichment analysis identified several key pathways associated with LVFP treatment of MF, including those involved in prostate cancer, lipid metabolism, atherosclerosis, and insulin resistance signaling. (Figure 3C; Supplementary Table S3).

### 3.6 Molecular docking verification

**Figure 4:**
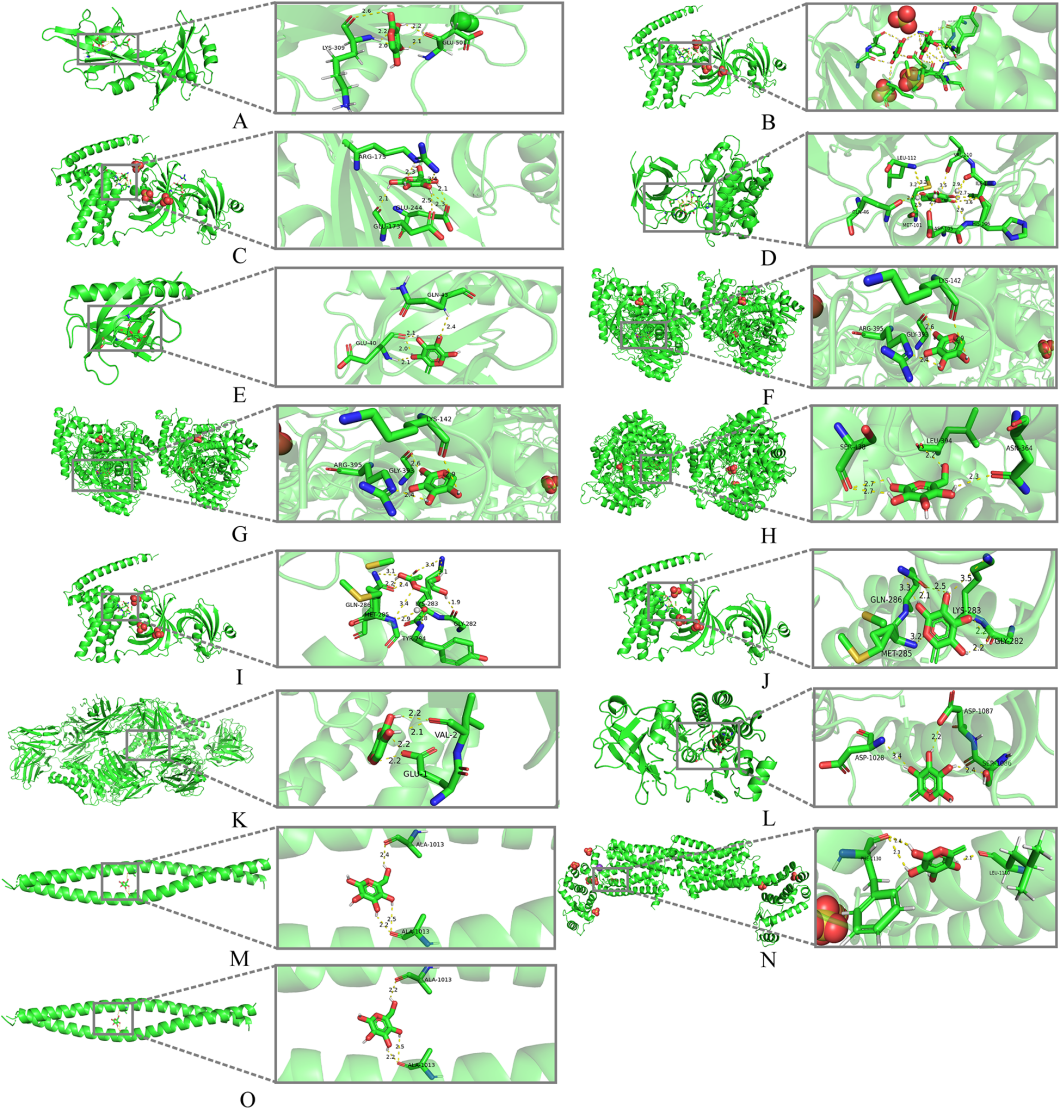
Molecular docking models of LVFP with possible core anti-MF targets. **(A)** SRC (1NZL) -Arabinose; **(B)** HSP90AA1 (5NJX) -Arabinose; **(C)** HSP90AA1 (5NJX) -Galactose; **(D)** GSK3β (2o5k) -Galactose; **(E)** AKT1 (3QKM) -Rhamnose; **(F)** ENO1 (7V67) -Arabinose; **(G)** ENO1 (7V67) -Galactose; **(H)** ENO1 (7V67) -Glucose; **(I)** HSP90AA1 (5NJX) -Glucose; **(J)** HSP90AA1 (5NJX) -Rhamnose; **(K)** IL-2 (7DR4) -Arabinose; **(L)** VEGFR2 (3WZE) -Rhamnose; **(M)** RHOA (1UIX) -Galactose; **(N)** PKM (7R6Y) -Rhamnose; **(O)** RHOA1 (1UIX) -Glucose.

We performed molecular docking between the monosaccharide components of LVFP and the 9 hub genes, and calculated the binding energies and analyzed the interactions. As a result, rhamnose exhibited a strong binding affinity for AKT1 (−7.36 kcal/mol) and VEGFR-2 (−8.65 kcal/mol). In contrast, galactose showed favorable binding interactions with GSK3β (−7.68 kcal/mol), which is an important negative modulator of the canonical Wnt pathway (25). (Figure 4; Supplementary Table S4).

## 4 Discussion

This research investigated the therapeutic effects of LVFP on MF and clarified its mechanisms and effectiveness through animal experiments, network pharmacology, and molecular docking techniques.

ISO-induced myocardial ischemia is a standard model used to evaluate the cardioprotective effects of various pharmacological interventions. Continuous stimulation of β-adrenoceptors increases the production and release of collagen-1 and collagen-3, which ultimately leads to detrimental MF (26). This model replicates the pathological scenario of MF observed in patients with acute myocardial infarction. Male C57/BL6J mice received subcutaneous isoproterenol at 5 mg/kg on day one, then 2.5 mg/kg daily for 13 days, inducing MF. This dosage and treatment period led to a higher incidence of MF with a reduced mortality rate. Compared to mice treated with ISO, LVFP treatment resulted in a significant decrease in myocardial fibrosis levels, as indicated by the HW/TL ratio and the fibrosis biomarker HYP. Masson's trichrome and Sirius red staining were employed to detect MF, primarily located in the endocardium. The ISO group exhibited increased ECM deposition and a significantly higher heart index compared to the control group, confirming the successful creation of an MF model. LVFP administration significantly reduced fibrosis levels compared to the ISO group. Furthermore, LVFP resulted in an increase in both EF and FS, indicating its myocardial protective effect.

The results of network pharmacology analysis demonstrated that LVFP has a broad range of potential targets and biological activities associated with MF. By analyzing the intersection of drug targets and disease genes, we identified key protein targets potentially involved in the anti-fibrotic effects of LVFP. In addition to the positive effects on the structural and functional aspects of the heart, LVFP treatment also exhibited beneficial effects on the molecular and cellular processes underlying MF. Through *in vivo* experiments and network pharmacology, LVFP was found to counteract ISO-triggered collagen buildup outside cells in mice via multiple pathways and targets. These findings elucidate the mechanistic basis of LVFP's anti-fibrotic effects and underscore its therapeutic potential for MF.

There are several limitations to our research that should be acknowledged. Although LVFP is crucial in treating MF, it remains uncertain whether its therapeutic benefits are specific to certain cell types. Further experiments are required to elucidate the mechanisms underlying MF inhibition by LVFP.

## 5 Conclusion

In conclusion, LVFP demonstrated potential as a therapeutic agent for heart-related disease by effectively attenuating MF. Further investigations into the mechanism of action and clinical applications of LVFP are warranted.

## Data Availability

The original contributions presented in the study are included in the article/Supplementary Material, further inquiries can be directed to the corresponding author.

## Footnotes

^1^ https://www.lilab-ecust.cn/PharmMapper/

^2^ https://www.genecards.org

^3^ http://www.uniprot.org

^4^ https://www.xiantaozi.com

^5^ https://string-db.org

^6^ https://www.xiantaozi.com/

^7^ http://www.pdb.org/
