# Supplementary material for: A comprehensive study on the impact of Ligustrum vicaryi L. fruit polysaccharide on myocardial fibrosis through animal experiments, network pharmacology and molecular docking
Source: Front Cardiovasc Med. 2025 Feb 20;12:1470761. doi: 10.3389/fcvm.2025.1470761 (PMC11882575; doi:10.3389/fcvm.2025.1470761)
Supplement: Supplementary file 1 [file Datasheet1.docx]

Supplementary Material

## Table1 Four monosaccharides of LVFP

| Molecule name | Molecule structure | Molecule weight（g/mol） | Molecule ID | Alop | OB% | DL |
| --- | --- | --- | --- | --- | --- | --- |
| Glucose (Glu) | 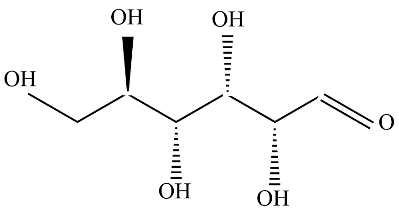 | 180.16 | Mol000734 | -2.68 | 24.44 | 0.03 |
| Galactose (Gal) | 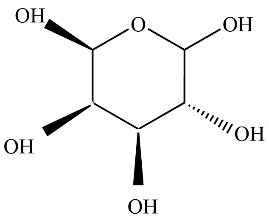 | 180.16 | Mol010203 | -1.93 | 10.22 | 0.55 |
| Arabinose (Ara) | 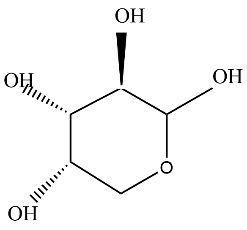 | 150.13 | Mol000382 | -2.17 | 1.87 | 0.22 |
| Rhamnose (Rha) | 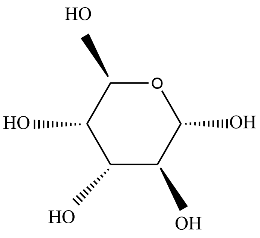 | 164.16 | Mol000424 | -1.62 | 50.50 | 0.04 |

**Table 2** Gene lists of Venn and GOKEGG analysis

| **LVFP target  gene name** | **LVFP target  gene name** | **MF Gene  Symbol** | **MF Gene  Symbol** | **MF Gene  Symbol** | **MF Gene  Symbol** | **MF Gene  Symbol** | **MF Gene  Symbol** | **LVFP target gene name  and MF Gene Symbol** |
| --- | --- | --- | --- | --- | --- | --- | --- | --- |
| MTAP | ldha | CFTR | SRP72 | UBE3A | MIR328 | COQ2 | CD79A | MTAP |
| PROK | LAP3 | TERT | EIF6 | RYR3 | PGR | XIRP1 | TERF1 | BCHE |
| acpS | ung | TGFB1 | ERCC6L2 | DDAH2 | OGG1 | ANXA11 | GJB1 | CDK2 |
| PPID | nlpI | ACE | SAMD9L | VIPR1 | TRIM72 | PPIC | AGK | ANG |
| nudE | uaZ | DSP | TSR2 | MIR660 | TNNI2 | CANT1 | TALDO1 | AKR1B1 |
| Ca5a | tgt | GCLC | MIR543 | RELB | NR1I2 | ITIH4 | THOC2 | PYGL |
| budC | DPYD | CLCN1 | PLA2G1B | NTRK1 | HELLS | ID1 | VPS33B | CRAT |
| NUDT9 | clcA | TERC | ITPR3 | PLCB2 | DNMT1 | SYNE1 | KCNE2 | CTSK |
| GRESAG 4.1 | metB | SERPINE1 | HNRNPA1P10 | ANO9 | COL14A1 | FBLN2 | MALAT1 | DPYD |
| BCHE | rbsD | TNF | PNPLA8 | DLST | CEL | CCT4 | CHGA | B4GALT1 |
| uprt | pll | MIAT | BMAL1 | SSH2 | AHRR | IPO9 | EZH2 | PYGM |
| pyrE | SOT12 | HFE | TNKS2 | MIR29B2 | SIK1 | COL7A1 | PLEC | MMP7 |
| abfA | glcB | TUBB3 | TLR10 | SYNPO | MIR133A2 | SERPINA12 | PIK3CA | PDPK1 |
| CA12 | fccA | HMOX1 | C5orf15 | BACH1 | BRD4 | MYLK2 | PRG2 | GSK3B |
| Syt1 | CTSB | SERPINA1 | FKBP10 | H6PD | PSTPIP1 | TNFRSF25 | IRS1 | PTPN1 |
| Itpr1 | gdhB | CCN2 | MPP7 | PROS1 | CAST | HTRA2 | MIR196A1 | PAH |
| CIP1 | ppgmk | IL6 | PRKACB | CGB3 | PTGES | IGFBP2 | MMP14 | SRC |
| rbsB | spoII | ABCA3 | FARP2 | MIR134 | COL6A3 | PRDX2 | SREBF1 | ENO1 |
| rbsK | ANG | CRP | FRZB | MMP28 | TRPV6 | DPT | SIRT3 | REG1A |
| glcB | zipA | CXCL8 | SGCG | MMP20 | LTBP1 | SMOC2 | KCNQ1OT1 | CTSB |
| araC | AKR1B1 | IL10 | KCNH1 | DCLRE1B | GATA1 | MAP2K4 | MIR32 | CCNA2 |
| cel6A | ptd | MMP9 | CFB | RMRP | CHRM3 | ABCD1 | MIR338 | CA2 |
| GLU1 | udp | EDNRA | WNT10B | SERPINB8 | ADIPOR2 | ATP5F1A | KLF4 | ALDOA |
| Mbl2 | kdsA | MYH7 | CCNA2 | MLF1 | ACTN4 | ABCA4 | ALG5 | NOS3 |
| CDK2 | tyrS | ITGB3 | E2F3 | VASH1 | CACNA1A | RFX3 | ZMPSTE24 | MMP3 |
| spoII | galM | TNNI3 | E2F2 | POMGNT1 | CALCRL | MIR375 | UCP2 | AHCY |
| ANG | purA | LTA | HNRNPL | UBR3 | IL18BP | MSRA | IFNA2 | RNASE3 |
| ctxB | AMY1A | FAM111B | NEK3 | ETS2 | RXFP1 | UCP3 | NGF | IL2 |
| comB | PYGM | SCN5A | HNRNPAB | AQP4 | MYBPC2 | ILF3 | MAPK10 | XDH |
| fcl | glkA | ESR1 | FCAMR | GLO1 | BCS1L | MAPKAP1 | NBEAL2 | C1S |
| AKR1B1 | MMP3 | IL1B | MIR218-1 | CXCL3 | RACK1 | LMAN1 | SKIL | RAB5A |
| lacS | MDL1 | CCL2 | TSPAN14 | HBB-LCR | NDUFB11 | FGF9 | APCS | LGALS2 |
| folB | C1R | EDN1 | KIR2DL1 | TLN1 | PITRM1 | MIRLET7B | CASQ2 | LGALS3 |
| PYGL | hemA | TNNT2 | JUND | TERF2IP | HABP2 | EHD1 | SLC25A4 | PGK1 |
| ppgmk | apx1 | MIF | CHD7 | PTMA | RDX | TREH | MIR148A | HSPA8 |
| GALK1 | PKM | STN1 | GFI1B | CUL3 | FHL1 | DUSP1 | BCL2L1 | MDH2 |
| SP_0314 | rad50 | OLR1 | SDK1 | RB1 | IVD | MAPK9 | F2RL1 | ADSS1 |
| VC_0232 | mtnN | VEGFA | SMCHD1 | SFRP5 | MIR15A | REST | KCNE1 | PARP1 |
| Mbl1 | cbh1 | TIMP1 | CD160 | PAX7 | SOX6 | ANXA3 | WT1 | HSP90B1 |
| nlpI | eltB | F7 | SCN1A | SGO1 | MSR1 | GRB2 | FOXP3 | CHI3L1 |
| cbh1 | algD | MMP2 | FEN1 | STK16 | ATG5 | NPHS2 | LAMA4 | TGFBR2 |
| ARC5A | aroD | PSMA6 | MT-TR | PLD2 | IFNGR2 | SPNS2 | LEPR | FCAR |
| MDL1 | lacZ | NPPB | COL15A1 | ATF1 | MCL1 | GCH1 | NPHS1 | PIM1 |
| DHFR | CTSK | CD36 | ARAF | TPM3 | MIR494 | DAAM2 | POLG | TPI1 |
| thiD | VC_0232 | FAM13A | TYK2 | CAVIN1 | COL18A1 | TUFM | MEN1 | BAG1 |
| YMR087W | panC | TTN | HSPB7 | TMEM70 | SCAI | ACADM | GSK3B | HSP90AA1 |
| rpiB | ILV2 | TLR4 | TRIM50 | IL18RAP | C5AR1 | TNXB | GATA2 | IGF1R |
| lsrB | ispF | APOB | TSC22D3 | MAP2K2 | NDUFA13 | CASP2 | GJD2-DT | TGM2 |
| kdsA | IMPA1 | PLAT | SCG5 | HNRNPM | SMURF2 | PLIN2 | IL23R | RHOA |
| Art2b | araF | FCGR2A | FRMD4A | CYP2R1 | FCGR3B | MID1 | FKRP | MMP8 |
| LTP1 | f17aG | STX1A | CUL7 | MIR99B | MIR101-1 | MT-TS1 | TNFSF11 | PLAU |
| cobT | HK1 | NOS3 | B3GALNT2 | HLA-DRA | C12orf43 | HSP90B1 | CPT1A | PRKACA |
| CRAT | xynA | JAK2 | LARGE1 | MCM6 | TRPS1 | PNKD | DOLK | DPP4 |
| thyA | MNS1 | F13A1 | TNFRSF9 | MASP1 | ATP1A1 | ADRA2A | FHL2 | HINT1 |
| ptd | Nos1 | ALB | ETFDH | GP5 | CYP19A1 | IRAK3 | MAP2K1 | NR1H2 |
| purA | mglB | IFNG | COX6B1 | BMP5 | TARS1 | IL17RA | HLA-C | PPIA |
| RSc3288 | yecD | MPO | MICOS13 | QKI | AARS1 | SIL1 | VEGFC | PDE4D |
| YPTB1668 | xylH | CTNNB1 | ARF1 | IL21R | UCHL1 | PEX10 | PRKCD | CFD |
| tgtA | bglT | SLC9A3 | BTRC | CYP27B1 | MIR30D | PEX13 | PTH | F2 |
| rbsD | IMPDH | MMP3 | KBTBD13 | ATL1 | MIR93 | ADRA2C | CYP27A1 | LAP3 |
| deoD | alpha-Man-IIa | LGALS2 | GIMAP5 | VLDLR | SRSF2 | PRSS23 | CDH23 | PKM |
| rad50 | fosA | MMP1 | RFXAP | RAB3GAP2 | SSB | CNTLN | MYL1 | HSD11B1 |
| pdaA | cmoJ | REN | MTHFD1L | TAGLN2 | RPS6KB1 | NEDD4 | CCN1 | CTNNA1 |
| CTSK | OTC | CAV1 | MIR532 | CYP21A2 | AKAP9 | AKT2 | MIR222 | MMP12 |
| folK | cbh2 | IL1RN | WIF1 | AKAP12 | ATF4 | IL27 | ITGB2 | AKT1 |
| HA | rntA | KCNN4 | MIR636 | ADRA1A | NOG | KDM4C | MIR130A | KDR |
| folP | Pcbd1 | ELN | FOXP1 | BID | AREG | MCU | CHIT1 | SOD2 |
| jgt | jgt | PPARG | ADRA1B | IGFBP6 | U2AF1 | LUM | VAMP8 |  |
| alpha-Man-IIa | IGF1R | ELANE | NEK4 | ZIC3 | KLK1 | MAPK7 | CYP2D6 |  |
| yicI | ykoF | LPL | RN7SL1 | SFRP4 | PNPLA2 | MYF5 | SLC8A1 |  |
| suhB | BACE1 | AGT | RGS4 | GSDMD | TOP1 | REG1A | HRAS |  |
| punA | rsuA | INS | ULK4 | ATP2B1 | SNRNP70 | PF4V1 | SOX17 |  |
| mtnN | pdaA | HGF | ALKBH8 | THADA | IL36RN | TDGF1 | MEG3 |  |
| DPYD | AHRI | APOE | GCM1 | CCAR2 | NRAS | ADCY1 | USB1 |  |
| VMA5 | HIS7 | TP53 | PGPEP1 | SYK | BMP15 | GYG1 | SST |  |
| TK | TM_0306 | MYBPC3 | DNPH1 | MIR194-1 | ADAMTS1 | HDAC1 | LAMA3 |  |
| PAP1 | fumC | IGF1 | ABTB2 | RFX2 | NBAS | LYN | MERTK |  |
| tgt | hmuO | AGTR1 | HHATL | MT-ND6 | CXCR5 | TRPC3 | SAA4 |  |
| PGRP-LB | glbN | HLA-DRB1 | MACROD1 | RAB7A | IKBKG | BMP10 | SDC1 |  |
| metB | ispDF | MEF2A | TIPRL | SMARCAL1 | COG7 | SLC19A2 | GH1 |  |
| B4GALT1 | PDPK1 | LMNA | C17orf80 | KLF2 | TRAF6 | WWP2 | CDKN1C |  |
| PYGM | fcl | SMAD3 | CTXN2 | SH2D1B | GPBAR1 | MDC1 | LIMK1 |  |
| tetX | TK | THBD | CUL4B | STAT6 | AIF1 | PRKAB1 | JAG1 |  |
| PAE0790 | ARC5A | GSTM3 | TRH | BLZF1 | SERPINF1 | CTHRC1 | MECP2 |  |
| hmuO | XOG1 | ICAM1 | CHCHD10 | BCKDHB | ARSA | HSPB8 | CAV3 |  |
| fldA | PAH | APOA1 | ASGR1 | CGAS | MORN1 | DNAH5 | CACNA1S |  |
| queA | HSD11B1 | HIF1A | SORCS2 | FANCI | SKI | YWHAE | PDPN |  |
| eltB | gpt | SCNN1A | AUTS2 | NFATC3 | DCAF8 | POMT1 | LOC107548112 |  |
| Ada | murA | MIR21 | RCC1L | CCT8 | RNASE2 | ATG7 | MYC |  |
| Gria2 | MMP8 | LRP8 | CRABP2 | DYM | TTPA | LRPAP1 | ERAP1 |  |
| MUR1 | bglA | NPPA | TRIB2 | YTHDF3 | CRHR1 | RUVBL2 | XIAP |  |
| RHEB | tgtA | PKHD1 | VAC14 | EPHX2 | KRT5 | ADAMTS5 | ATP13A3 |  |
| gaI | CA2 | F2 | SEC24D | SMAD9 | APLNR | CACNA1B | FHOD3 |  |
| HK1 | CBR1 | IL18 | COQ5 | TFAP2A | MIR324 | AKR1A1 | RBCK1 |  |
| thrB | PRSS1 | IL4 | RFFL | PIAS1 | DSC3 | SLC1A5 | AGL |  |
| Grik1 | B4GALT1 | CALR | COLEC11 | CKLF | PPP2CA | HUWE1 | SPTAN1 |  |
| icd | CTNNA1 | ADRB2 | BBOX1 | SNTA1 | IGF2R | SF3B1 | WDPCP |  |
| ppc | FBP1 | COL1A1 | DMGDH | KPNB1 | CD1C | XCL1 | CCR3 |  |
| cbh2 | purT | LDLR | CPA3 | SAA2 | PIM1 | ATP2B4 | MYOD1 |  |
| MMP7 | Prkaca | SELP | FGFR4 | SELENON | XIST | COL6A1 | CX3CL1 |  |
| cel3 | mtrB | MB | MVK | HLA-DRB9 | MT-ND4 | ATP5F1E | LBR |  |
| mutY | nprS | ENG | ATP6V1A | IGFBP4 | PDGFC | LRP2 | CYP17A1 |  |
| gpt | MUR1 | PTGS2 | CSF2RA | PLA2G3 | ACTG2 | HOTTIP | GATM |  |
| HPRT | VAOA | CCL11 | CRELD1 | ARRB1 | TNFAIP6 | SCD | MYH7B |  |
| bglA | Ahcy | NOS2 | SELENBP1 | TFEB | NEDD4L | HAS1 | ARFGEF1 |  |
| trpA | Ada | MIR210 | MIR424 | SPRY1 | NUP93 | C1QTNF6 | HBA1 |  |
| xylA | hisG | STAT3 | STK39 | CDR1-AS | CYBRD1 | TCF21 | DGUOK |  |
| XOG1 | mdlA | LGALS3 | PDLIM1 | ROR2 | TJP1 | MT-TC | ADAMTSL1 |  |
| dxr | lacA | AGER | SLC22A11 | PAFAH1B1 | AGRN | SERPINA4 | MIR30B |  |
| fmo1 | dcd | GCLM | SLC17A3 | MIR125B2 | ATP6AP2 | MIR382 | XRCC1 |  |
| CDA | PAE0790 | HPS1 | SYTL1 | CHRNA3 | LDHA | GNRH1 | PRKAA1 |  |
| LDHB | cel3 | FGF2 | AURKAIP1 | P2RX6 | ALOX15 | PDIA3 | TNFSF10 |  |
| PDPK1 | KCNMB2 | SPP1 | SERPINA2 | PNOC | ITLN1 | MAP1LC3B | IL1RAPL2 |  |
| TM_0306 | YPTB1668 | CXCL12 | EHD3 | MAP2K6 | SGCD | ELOC | KRT19 |  |
| PRSS1 | pabC | FN1 | CFAP251 | CFAP410 | MIR186 | IGFBP7 | PRKCE |  |
| cmoJ | chb | IL17A | MT-TQ | CLN3 | HULC | TPSAB1 | MT-CO3 |  |
| trpD | LGALS7 | KNG1 | MT-TS2 | DBN1 | SIRT6 | SLC22A4 | CYP2E1 |  |
| pgiA | dxr | MUC1 | MT-TW | IRX1 | LIPE | SOAT1 | TUBB4B |  |
| fucI | eda | SCNN1B | TEP1 | DLK1 | APOC2 | PIK3CB | IGF1R |  |
| rmlC | CSNK1G2 | IL13 | KAT2B | MIR106B | TRMU | ENO2 | TYR |  |
| dhbE | camC | CCL5 | RAB37 | ITGA7 | WNT5A | TP53COR1 | FABP2 |  |
| galM | MMP12 | IL1A | ALG14 | CYP24A1 | FGFR2 | SLC39A8 | HOXA1 |  |
| apx1 | IGLV2-8 | BMP6 | LARGE2 | PRKAR2A | KLK3 | MIR222HG | HLA-G |  |
| ispDF | gag-pol | MPL | COL20A1 | NLRX1 | TRPM6 | SERPINA6 | ALPK3 |  |
| CPB1 | Gria2 | PCSK9 | LAMC1 | SLC4A5 | IL25 | SPTBN1 | STAC3 |  |
| fccA | rhaD | SCNN1G | VPS13A | SMOC1 | NDUFS7 | ZFPM2 | CEBPB |  |
| GSK3B | thrB | CCT7 | TNKS | KCNN2 | UGT1A1 | FOXC1 | FOXO1 |  |
| RNASE1 | pgiA | TULP3 | FCGR1A | ACADL | ERCC6 | DMRTA1 | TRIM32 |  |
| ME2 | SORD | ACTC1 | NECTIN2 | MFN2 | MSN | MTO1 | TBX20 |  |
| accC | rraA | NLRP3 | BIRC3 | NR2C2 | PRKCB | PPP1R10 | NUP107 |  |
| panC | fucI | TNFSF4 | RARB | NCEH1 | HDAC4 | CD5L | BLOC1S1 |  |
| algD | ABD1 | ALOX5 | TRPM5 | OXT | MIR200C | AASS | YAP1 |  |
| CHI1 | queA | TGFB3 | PHEX | MTUS1 | ACVR1B | DDRGK1 | TNFAIP3 |  |
| HIS7 | blaR1 | CCR6 | NPR3 | MTUS2 | FLNC-AS1 | ATP2A3 | FGG |  |
| PTPN1 | TT_P0059 | CASP3 | TBXA2R | HLA-DPB2 | PRKN | S100A6 | MYH11 |  |
| bglT | CHI1 | CSF3 | PSEN2 | HPRT1 | TP63 | SI | SPHK1 |  |
| purF | TNK2 | F3 | CDK6 | CTNNA1 | FTO | CORIN | TIMP4 |  |
| nanH | MMP7 | MTHFR | MKRN1 | SCP2 | NEXN | TNXA | MIR409 |  |
| MC1 | buk2 | CP | PIGT | HBA2 | SURF1 | SMO | MIR483 |  |
| TNK2 | MSDC | NHP2 | CELSR1 | GNA11 | LAMB2 | MIR367 | APOA1-AS |  |
| ABD1 | tmk | ADIPOQ | PRIM2 | VAMP3 | WWTR1 | RAMP1 | RFC2 |  |
| PAH | HN | SOD1 | RAB23 | PAWR | RAMP2 | C7 | NCF1 |  |
| SRC | AKT1 | CCL3 | LSM2 | MYO18A | CFI | H2AX | TNFSF13B |  |
| lacA | GSK3B | LEP | EMSY | DIABLO | MIR451A | AIP | COL2A1 |  |
| gmhA | phyB | AKT1 | MTHFSD | TMPRSS6 | ANKRD1 | PEX14 | CYSLTR2 |  |
| NA | KDR | VCAM1 | ADSL | YWHAG | PRKDC | PEX19 | TUG1 |  |
| araF | TRI5 | MIR155 | ALG12 | CCT2 | WNT11 | PEX3 | INHBA |  |
| Aaar | EPHB4 | IL33 | DYNLT1 | OXTR | G6PC1 | PEX26 | ADORA3 |  |
| GGTA1 | PTPN1 | AGTR2 | SSPN | LAMB3 | SDC4 | PEX11B | EHHADH |  |
| B3GAT1 | PLAU | PIK3C2A | PTPN6 | MAP2K7 | TNFSF14 | PEX12 | PRL |  |
| pyrF | fmo1 | MBL2 | DIDO1 | GNAQ | CAP1 | ITGA6 | KRT7 |  |
| tvaII | LDHB | HMGB1 | PANX2 | RARRES2 | EPHA3 | KLF6 | SDHA |  |
| f17aG | coaD | VWF | DPAGT1 | PAK2 | AMBP | PGK1 | TMSB4X |  |
| Fbxo2 | pep | NPHP4 | MPDU1 | RUNX2 | MIR33A | NME2 | CRH |  |
| HN | nrdD | PTX3 | MDH1 | ANK3 | HOXD13 | HSPA12B | PPIA |  |
| glkA | PH0828 | HP | SVIP | ABCB8 | HYOU1 | WNT3A | SBDS |  |
| hxpB | CDA | LPA | SPTB | FGF4 | P2RY11 | PFN1 | HAND2 |  |
| OTC | AK | IRF5 | TMPRSS2 | SCARNA2 | NRP1 | SGCA | LOC110121486 |  |
| Fas1 | php | ACE2 | FAM20C | RGS2 | HCP5 | TAB1 | MPIG6B |  |
| ams | SRN | RPA1 | CD33 | FPR1 | CS | EIF2AK3 | RPL3L |  |
| Cftr | tetX | POSTN | POLE2 | MIR873 | LYZ | SMTN | TRPV1 |  |
| fucA | cgt | GPT | CD83 | YWHAB | NOL3 | FDFT1 | GFAP |  |
| ENO1 | Fbxo2 | TTN-AS1 | CLEC4C | IFT25 | GOSR2 | FCGR3A | NEU1 |  |
| bls | dhbE | PON1 | POTEF | HSPA9 | ZEB1 | LMNB1 | DNAJB6 |  |
| AVD | SOD2 | TINF2 | MIR149 | TPK1 | GYS1 | LAMB1 | CASP8 |  |
| REG1A | aroH | F5 | LGMN | SARS2 | GLUL | MYH1 | AR |  |
| aroQ | hxpB | HPS4 | REG3G | MIR369 | IFT81 | ACTN3 | TGIF1 |  |
| PAK6 | npr | ENPP1 | NLRP1 | COL6A5 | DUSP19 | QDPR | TRPM4 |  |
| pyrC | xylA | MIR145 | CCDC71L | KCNIP2 | AXIN1 | CACNA1D | FSTL1 |  |
| nagB | LTP1 | IL5 | SUN1 | NDUFAF3 | MT-CO1 | COL5A2 | MYBPC1 |  |
| FDPS | abfA | SERPINC1 | SUMO1 | ERCC2 | APIP | ACKR2 | IL15 |  |
| IMPA1 | phaA | PLG | PTK2B | SERPINA7 | CCL20 | LTBP2 | MDH2 |  |
| idi | yicI | TLR2 | MEST | DOCK2 | KEAP1 | CPOX | ORAI1 |  |
| ftsZ | TPI1 | CXCR4 | BNC2 | RPS6 | FOXO3 | SIRT2 | PDE4A |  |
| Grik2 | RNASE1 | BCL2 | MYL11 | PLCG2 | TPO | MIR1291 | ADSS1 |  |
| P2 | deoD | PDGFRA | CRHR2 | WNT2 | IL32 | MT-RNR1 | COL4A5 |  |
| CTSB | YMR087W | TNFRSF1A | TRIM74 | LOC119369037 | MT-TE | MIR379 | DPP6 |  |
| LOC107808322 | suhB | MYL2 | INCENP | CAPN3 | PITX2 | PTGER4 | IL17F |  |
| SRN | GGTA1 | MYH6 | CTSZ | ELK1 | CLDN5 | E2F1 | CCL26 |  |
| rntA | CEL7B | PECAM1 | SCRG1 | C1QBP | ADAR | PHB1 | SOCS1 |  |
| glbN | CFD | SH2B3 | COX17 | LRPPRC | MIR20A | TIA1 | ENO1 |  |
| aguA | F2 | PKD1 | GBF1 | HSPA1B | TRIM33 | ANK1 | NR1H3 |  |
| RAB11A | trpA | MIR499A | ENO3 | YWHAQ | FH | PPA1 | CHKB |  |
| mgsA | DHA2_112885 | DNASE1 | ZC3HC1 | WNT9B | PDPK1 | ENPP3 | CD46 |  |
| CCNA2 | GALK1 | TET2 | TTF2 | MYSM1 | IAPP | MYH3 | TFRC |  |
| CA2 | thyA | TPM1 | PRKCZ | NPEPPS | POT1 | MYH8 | MYH10 |  |
| TPI | NA | NOP10 | MAGED2 | PCMT1 | MIR199A2 | MYH13 | OSM |  |
| ALDOA | codA | NHERF2 | APRT | NSFL1C | CFHR5 | FABP4 | COL6A2 |  |
| cmk | PRXC1A | S100A8 | TACR2 | C4BPA | USP9X | PDX1 | CALM1 |  |
| mglB | SP_0314 | ITGAM | BPHL | BIRC2 | NDUFS4 | TRIM21 | FGF21 |  |
| NOS3 | rbsB | SMAD2 | BCL11A | MIR331 | RUNX1 | COMT | CAMK2G |  |
| coaBC | gaI | LOX | MYO18B | ITPR1 | LGALS4 | MIRLET7A1 | ABCC2 |  |
| MMP3 | cel6A | FAS | PDLIM3 | CCND2 | TCN2 | CDH13 | NRG1 |  |
| galE | folK | HSPA4 | TAF1A | MSLN | PROC | CTSH | POMC |  |
| L3 | estB | KCNQ1 | TRIM22 | DHCR7 | LIF | BCAM | IL16 |  |
| xynA | lsrB | CETP | PRELP | SMAD6 | IL18R1 | DHX36 | KCND3 |  |
| rhaA | araC | MMP7 | NID2 | FCGRT | DDX39B | PRDM8 | UBAC2 |  |
| chb | GLAA | SELE | TINAGL1 | SEPTIN4 | RECK | MEPE | MIR125A |  |
| At4g18930 | RSc3288 | ANXA5 | CYLD | PRKAR1B | SNRPA | S100P | MYH14 |  |
| blaR1 | GLU1 | SIRT1 | MATN2 | ITPKC | NUCB2 | RET | HADHA |  |
| phi | folB | DES | INTS4 | SLX4 | NTN1 | MAX | MTHFD1 |  |
| uao | thiE | COL3A1 | PCBP1 | KIR3DL1 | MAPT | IRAK4 | SLC5A2 |  |
| cat3 | accC | HSPA8 | FZD9 | MIR217 | SLC16A1 | ACO1 | PEX1 |  |
| AHCY | SRC | CD34 | FBN2 | KLF5 | MAT2A | MIR30E | EIF4H |  |
| SEC4 | crp | CD40 | SLC4A1 | DIO2 | ITGA1 | RPLP1 | NEAT1 |  |
| ribH | PDE4D | SRC | TFPI2 | DPM3 | FBLN5 | GYPA | AHSP |  |
| ILV2 | Crat | MIR17 | FBXL4 | HSPB2 | ALPL | CCDC93 | MIPEP |  |
| RNASE3 | rbsK | ADRB1 | NDUFAF5 | CRYAA | NRIP3 | RGS3 | XYLT1 |  |
| cca | thiD | CXCL10 | RNU4ATAC | TNFRSF12A | FBXO32 | TK2 | BTNL2 |  |
| ISG20 | HPRT | ADAMTS13 | UHRF1 | BTK | MIR98 | MAPKAPK3 | ABCC8 |  |
| IL2 | folP | INSL6 | HK2 | NOA1 | SLC2A4 | LRRC56 | SCN1B |  |
| purE | CDK2 | CCR2 | PARP2 | GSTA1 | NOS1AP | HDAC5 | DKK1 |  |
| rpiA | Syt1 | CD14 | IFI44L | ABHD5 | ALG1 | AIMP1 | MRTFA |  |
| TRI5 | axe1 | ABCA1 | WARS2 | ERBB3 | FCAR | PTHLH | SEMA7A |  |
| KCNMB2 | PYGL | EPO | CHD3 | M6PR | MIR148B | OPRK1 | TAFAZZIN |  |
| udp | AVD | DCN | PPCS | EGLN2 | DRD2 | HOPX | NT5E |  |
| ampC | ppx | SFTPD | MT-TN | TRPA1 | HSP90AB1 | AK1 | TYMP |  |
| XDH | phzF | B9D2 | NRIP1 | IL36A | C4B | CFP | IL37 |  |
| ulaD | DFR1 | MYL3 | PLA2G2D | CA9 | MMRN1 | NT5C2 | TRPM7 |  |
| C1S | CPA1 | FABP3 | MAPKAPK2 | IRF8 | ALOX12 | PHETA1 | CD63 |  |
| RAB5A | cobT | S100A9 | HNRNPA2B1 | GPR22 | ABCG2 | FAM219B | COL4A2 |  |
| bla | NR1H2 | SLC17A5 | TREML1 | FANCG | PSAP | MT-TF | CACNA1H |  |
| Grin1 | PPIA | CHI3L1 | CARMIL1 | SLC2A9 | AQP1 | ADAMTSL2 | LTBP4 |  |
| LGALS2 | CA1 | KRT18 | PLD1 | ACADS | SOS1 | LTC4S | MIR320A |  |
| PH0828 | MTAP | MYO5B | SLC9A2 | UTRN | TXNIP | SUV39H1 | AVPR2 |  |
| rraA | nudE | SFTPB | DSG1 | CYP8B1 | IMMT | MIR362 | DNMT3A |  |
| UCK2 | CA12 | PPARA | B4GALT1 | RBP1 | BDKRB1 | RFX1 | CXADR |  |
| trpF | mutY | LCN2 | KCNQ3 | CAPZA1 | CCDC65 | RABL2A | NAGLU |  |
| thiE | HSP90AA1 | ALMS1 | PTGER3 | ADAM10 | SLC10A2 | ALMS1P1 | PTK2 |  |
| AHRI | estF | IL2 | SUCLG1 | TNNI1 | GNB1 | PADI6 | BMP4 |  |
| BIRC7 | metE | FGA | HMGCL | ITGAE | ROCK2 | CYB5R3 | TJP2 |  |
| abfB | let-70 | LAMP2 | TACO1 | GLS | SREBF2 | PIGR | USP8 |  |
| luxS | xapA | JUP | PEBP1 | DIAPH1 | IDO1 | PRKCG | IL11 |  |
| VNG_1446H | ctxB | NR3C2 | ASZ1 | ATP5PF | PEX5 | CTH | EP300 |  |
| LGALS3 | ampC | NOS1 | ALDH18A1 | NDUFS2 | KIF13B | NFAT5 | MIR10B |  |
| gpmI | Mbl1 | MUC5AC | AVEN | MAPK11 | ADAM8 | DBNL | DYRK1B |  |
| Nos1 | BCHE | F2R | IDS | ADCY8 | MED23 | SHMT2 | CMH21 |  |
| MAN1B1 | PPID | IL1R1 | PHYH | TUB | SEMA3A | KLB | MYB |  |
| PGK1 | acpS | KCNJ5 | FLAD1 | RYK | APELA | FANCC | MT-TL1 |  |
| HSPA8 | dhlA | SMAD4 | MT-TK | NFATC1 | TPM2 | MAP3K20 | MICA |  |
| SRM | PROK | CDKN2B-AS1 | LCK | EIF5A2 | NUP210 | TCF3 | CELSR2 |  |
| MDH2 | HEM2 | SMAD7 | MIR377 | KCNK5 | POU5F1 | ICAM2 | ACKR3 |  |
| ispF | CIP1 | PDGFRB | CD109 | LDHAL6A | HNRNPA1 | MIR153-1 | CNR2 |  |
| cex | GRESAG 4.1 | WRN | PDK4 | CKMT1A | HTR3A | CYTOR | MIR26A1 |  |
| CPA1 | lip | SOD2 | ORM1 | NEBL | PPP1R15A | ICOS | LEPQTL1 |  |
| TK1 | gyrB | NFE2L2 | MAT1A | IL12RB2 | VEGFD | SMN2 | EMD |  |
| Nos2 | QPCT | MAPK14 | ATP6V1F | RPS14 | MCAM | ZNF699 | AKR1B1 |  |
| celS | rsbQ | GSN | KLHL3 | PLK4 | TBX3 | ERV3-1 | PTCH1 |  |
| CES1 | PNP | PDGFB | SPG11 | POMGNT2 | PEDS1 | P2RX4 | LGALS3BP |  |
| ispA | PAP1 | PKP2 | SAG | RHBDF2 | LOC110006319 | GET3 | MFGE8 |  |
| gapA | WBAI | CDKN2A | C8B | RILPL1 | C1QTNF3 | INPP5B | CLEC7A |  |
| ADSS1 | AMY1A | PRKAG2 | C8A | PPP2CB | CYSLTR1 | MARCHF2 | CYP7B1 |  |
| ushA | fccA | HMGCR | EFHC2 | GLUD1 | MT-CO2 | ECM1 | ITGAV |  |
| zwf | ALDOA | MIR208B | IGLON5 | SMN1 | CPT1B | DPM2 | CCDC115 |  |
| ndkC-1 | tgtA | DYNC2H1 | IRF3 | GMPPB | SRI | TRNT1 | CYP3A5 |  |
| gabT | udp | EGFR | CRLS1 | IDUA | ARRB2 | PSMD5 | NFKBIA |  |
| PARP1 | phzF | LOC123956257 | MLANA | CTPS1 | NR1D1 | TRAF3 | GAS5 |  |
| celM | jgt | NKX2-5 | SMC3 | PLA2R1 | FCN3 | COX4I1 | TH |  |
| HSP90B1 | LDHB | TGFB2 | MIR381 | DYRK1A | TRAP1 | MIR433 | F12 |  |
| PROK | yicI | PRTN3 | REEP5 | TIE1 | ASXL1 | CLPB | CCL7 |  |
| Mbl2 | xynA | APOC3 | NPTXR | PKP1 | NOTCH4 | ACTG1 | PRG4 |  |
| GLAA | PNPO | ADORA1 | PROKR1 | LUCAT1 | MIR197 | TRMT5 | KANK1 |  |
| cel6A | malE | TNFRSF11B | PLP1 | PTH1R | IL10RA | ACLY | KLKB1 |  |
| CIP1 | CTSK | CST3 | LRP1B | TRIM28 | BICD2 | VDAC2 | GCG |  |
| acpS | PRSS1 | ABCG5 | ARID2 | CAV2 | ACTN1 | NCAPG | SLC34A1 |  |
| PAP1 | BAG1 | CD40LG | GAP43 | FAM20A | IL17C | LIMA1 | ERBB2 |  |
| GRESAG 4.1 | ansB | S100A1 | PODXL | NDUFS3 | PLCB3 | MT-ND3 | MIR1-1 |  |
| mutY | HEXB | VIP | PCCB | WNT3 | RAB27A | ASAP1 | IKZF1 |  |
| pyrE | mglB | SERPINA3 | UBIAD1 | RAP1A | GRK5 | GFM1 | BIRC5 |  |
| gaI | BST1 | PTPN11 | POR | DGKB | C11orf65 | SPG7 | ZC3H12A |  |
| CHI3L1 | panC | CSF2 | KDM6A | TLR8 | MDK | TIMM17A | DSG2-AS1 |  |
| bop | icd | MMP8 | ARSL | RORC | MIR124-1 | CXXC5 | GPD1L |  |
| ohr | HSP90AA1 | IFT172 | FLCN | BBC3 | HMCN1 | TIMM17B | PLIN1 |  |
| BCHE | IMPA1 | JUN | SLC2A6 | CD151 | TPM4 | COL22A1 | IL4R |  |
| estF | PGK | HSPD1 | EVA1C | PIFO | CFLAR | RLN1 | ADH1B |  |
| MTAP | HPRT | SOD3 | MITF | COL5A3 | RNF220 | SFRP2 | SETD2 |  |
| serA | MNS1 | DKC1 | OAS2 | CHD4 | NQO1 | LPAR2 | KRAS |  |
| mutB | TM_0306 | MIR126 | PRXL2A | CPS1 | TCF7L2 | ANKRD26 | HDAC9 |  |
| MSDC | PH0828 | PKD2 | CDC25C | COX10 | NDUFA1 | EMG1 | HNRNPUL1 |  |
| algD | rhaD | COL1A2 | GAMT | NDUFS1 | MMACHC | ITK | C5 |  |
| WBAI | bglA | CCR5 | SLC2A3 | NDUFA9 | MYL6 | MIR885 | XRCC3 |  |
| PPID | cbh1 | NHERF1 | PRKCI | XRCC6 | GOT1 | TAPT1 | LOC106627981 |  |
| AVD | actVA 6 | STAT1 | RCL1 | RECQL4 | AQP2 | DDT | G6PD |  |
| galK | lacS | BMPR2 | REEP3 | MIR542 | TNFRSF10A | MC4R | CCN3 |  |
| rsbQ | aroQ | CALCA | NRBF2 | HTRA1 | KAT6B | PDIA2 | RO60 |  |
| dhlA | AK | THBS1 | RNF4 | SETDB1 | GAST | CMKLR1 | TNFSF12 |  |
| uaZ | dxr | MIR30A | ROMO1 | APOO | OBSCN | PDE3B | TRIM8 |  |
| budC | IGF1R | HLA-B | SSPOP | HBS1L | PPP3CA | NUB1 | MYLK |  |
| ampC | deoD | ADM | MIR615 | TYRP1 | DKK3 | TBX21 | SRF |  |
| HSPA8 | phi | ITGA2B | ABCA12 | NSUN5 | MYO6 | SAMD9 | CYP11B1 |  |
| CA12 | pdaA | ALDH2 | ERAP2 | LEF1 | PLOD2 | LATS2 | HNF4A |  |
| thyA | B4GALT1 | ADORA2A | HHAT | MIR26B | TRIM5 | IL21 | TOM1 |  |
| xapA | ispDF | ACTA2 | HNP1 | PFKFB3 | MYOT | MTERF1 | MTPN |  |
| uprt | MUR1 | TGFBR1 | ZYX | SVIL | LPAR3 | HTR1B | VANGL2 |  |
| tthHB8IM | pgl | DSG2 | CD70 | AGPAT2 | PPARD | PLXNA1 | CEBPA |  |
| araC | rntA | PRKCA | CIRBP | CLEC3B | TNFRSF4 | IL17RC | NR5A1 |  |
| RNASE1 | fumC | GJA1 | GATA3 | GPR182 | STC1 | MIR184 | GHSR |  |
| Itpr1 | dhbE | MIR199A1 | NUBPL | MT-TT | ACACA | YARS2 | ELAVL1 |  |
| Mbl1 | cyp158a2 | GREM1 | LECT2 | TPH1 | TNFRSF6B | FAAH | BRCA2 |  |
| YML079W | Grik1 | ARG1 | MCCC2 | NPC1L1 | ANGPTL3 | CREB3 | MAP3K5 |  |
| PYGM | ENO1 | RYR2 | DIPK2A | NCF2 | NUDT6 | SLC30A10 | VCAN |  |
| abfA | hmuO | HAMP | AQP8 | PDGFD | C3AR1 | BCL7A | MIR27A |  |
| kaiB1 | icl | BMP7 | ACAN | FOXA2 | AKAP6 | SPATS2L | FADS2 |  |
| HA | apx1 | RETN | LMOD3 | SLC22A3 | ZFAS1 | CD2AP | COL5A1 |  |
| Syt1 | RHEB | MIR142 | MIR429 | RRM2B | PAH | HMOX2 | MIR200A |  |
| glkA | uvrB | GATA4 | HIPK1 | MIR574 | ADAMTS4 | DBH | DNAJC30 |  |
| TGFBR2 | TGM2 | ITGA2 | TMEM106B | MIR9-2 | MAGI2 | PDHA1 | TBX5 |  |
| folK | ISG20 | LRP6 | PARP12 | C5AR2 | WNT1 | EIF4EBP1 | CDKN1B |  |
| gdhB | PAE0790 | F10 | EHBP1L1 | HSD11B2 | SLC22A5 | GFRA3 | TXNRD2 |  |
| rbsB | fosA | ALOX5AP | CMIP | DSC1 | FLT3 | HGFAC | HAX1 |  |
| suhB | gmhA | C3 | EPHB2 | SLC6A6 | TGFBI | LIMS1 | DDR2 |  |
| ctxB | purT | GGT1 | DDC | NFATC2 | RECQL5 | PHKA1 | SETBP1 |  |
| cmoJ | Pcbd1 | DNAH8 | KCNN3 | PUS1 | DNM3 | PARVB | CD19 |  |
| cbh2 | RHOA | CXCR3 | RNASEH2A | SCAP | AHR | DSG4 | PRF1 |  |
| alpha-Man-IIa | Grm1 | ABCC6 | NDUFA6 | COL8A1 | LAMA5 | HOXA-AS3 | MBOAT7 |  |
| nrdD | MMP8 | FASLG | BOLA3 | SESN2 | PIGY | COG8 | RAMP3 |  |
| RSc3288 | PLAU | FGB | RNASEH2B | SCN8A | NES | HADH | NR4A1 |  |
| CDK2 | TNK2 | EGF | ISCU | BET1L | ANGPTL2 | MGME1 | MIR151A |  |
| PYGL | GSK3B | CBS | FSTL3 | GFPT1 | FBLN1 | PTPRJ | ITGA4 |  |
| accC | Ahcy | GLA | PTPRD | MESP2 | CHRM1 | GPX4 | FANCD2 |  |
| FCAR | PRKACA | MIR29A | NCF4 | SMC1A | JAK3 | STK3 | SGCB |  |
| Crat | XOG1 | ABCB1 | CLIC2 | TUBA1B | CFL1 | SPAAR | SQSTM1 |  |
| crp | ppc | ATM | LRP12 | AZIN1 | TAF1 | VMA21 | MAP1B |  |
| PNP | Ada | NEK8 | MIR454 | FERMT3 | ATRX | MT-TH | RLN2 |  |
| GGTA1 | gpt | ADORA2B | MIRLET7C | ANXA4 | P2RY1 | MIR501 | CBL |  |
| SOT12 | DPP4 | CX3CR1 | SYNM | BEST1 | CALM2 | RPL4 | XK |  |
| axe1 | rmlC | ABCC9 | FMOD | SPHK2 | GCKR | RPS28 | CD44 |  |
| comB | lacA | GDF15 | SYNJ1 | LAP3 | ALOX12B | JMJD1C | MT-ND5 |  |
| folP | cslA | CYP2C19 | UTS2R | CEP192 | MIR15B | AK3 | PINK1 |  |
| folB | ABD1 | LBP | KLF1 | ALX4 | SLC25A11 | CHRND | HPSE |  |
| kdsA | RIB3 | PF4 | FANCE | NOD1 | FYN | TMSB15A | EXOSC10 |  |
| trpA | queA | JPH2 | D2HGDH | CYP2B6 | FAH | MIR299 | PRKD1 |  |
| nagB | trpD | CFH | FOXRED1 | ADCY6 | HBG2 | NDUFA12 | CFHR1 |  |
| ppgmk | ams | CEP19 | MTFMT | RBBP4 | DNM2 | SYNGAP1 | RXFP2 |  |
| purA | aly | LDB3 | MRAP | SEC23B | SCX | LDLR-AS1 | NF1 |  |
| GLU1 | cel3 | IGFBP5 | SDHAF1 | RPL3 | BAK1 | KARS1 | MIR92B |  |
| cobT | TRI5 | CR1 | COA8 | PROK1 | ECE1 | RAD21 | RAC1 |  |
| rbsK | ykoF | TGFBR2 | HDGF | PDHB | CASP9 | TLR6 | FNDC5 |  |
| PAH | aguA | IGFBP3 | KIR2DL2 | SLC25A20 | MYH2 | SUN2 | CHAT |  |
| Nos1 | AMY2A | PLN | STEAP4 | TBX1 | MIR18A | GAL | ZNF627 |  |
| ANG | hisG | MIR378A | PPP1R12B | CLOCK | MIR590 | ADD3 | PROM1 |  |
| rho | CHI1 | GSTM1 | TFF1 | MIR106A | ITGB4 | STAT2 | FTH1 |  |
| nlpI | glmS | GSTP1 | SPON1 | ACO2 | KANSL1 | CDK1 | TRPC6 |  |
| glcB | CPB1 | TIMP2 | BCL2L11 | MLH1 | MIR23A | BMPR1A | TRPM3 |  |
| NA | DPYD | CD4 | TOR1AIP1 | GPNMB | CXCL13 | MIR509-3 | GDF2 |  |
| metB | fucI | SERPINH1 | CCNT1 | ID2 | CIB1 | OGA | VKORC1 |  |
| HK1 | DCK | APLN | FGR | TAOK1 | RLBP1 | NARS2 | MIR92A1 |  |
| DHA2_112885 | OTC | RNASE3 | MAP2K5 | FANCA | FASN | TICAM1 | LRG1 |  |
| MDL1 | HINT1 | SLC40A1 | POLR1C | MIR191 | TXNDC5 | CSNK2B | CLCNKB |  |
| ldha | fcl | MASP2 | SLC15A1 | BLK | ASAH2 | COG5 | CXCL6 |  |
| SRC | pobA | SERPINF2 | CCM2 | LRRC10 | RPS19 | PROKR2 | KIF1B |  |
| YMR087W | PDPK1 | MAPK3 | PTAFR | PCNT | NOTCH2 | ALPI | POLG2 |  |
| CEL7B | At4g18930 | CCL17 | PLPP3 | ABCC5 | FKBP5 | GP9 | HSPB6 |  |
| PIM1 | TGM3 | MIR486-1 | XPO5 | NLRP6 | CRAT | SULT2A1 | GTF2IRD1 |  |
| fucA | GPI | CCL18 | RPL27A | PDE4B | NONO | CD58 | SLC25A3 |  |
| thiD | KCNMB2 | THBS4 | ACAD10 | DRD1 | S1PR2 | BAG1 | HMGA2 |  |
| ispF | ansB | THPO | TMSB10 | NDUFS8 | CFAP46 | HTR4 | MIR154 |  |
| spoII | AKR1B1 | CAT | ATG9B | TEAD1 | PRODH | PLCG1 | MAS1 |  |
| FCY1 | MC1 | KCNJ11 | GCN1 | NRON | CAPN1 | MAVS | NPC1 |  |
| rbsD | def | ANGPT2 | LMAN1L | SLC34A3 | CD86 | INPP5K | TLR7 |  |
| SP_0314 | gltX | RBM20 | GIMAP6 | GRM8 | CAPN2 | RAD51C | KCNMA1 |  |
| udg | AKR1C3 | CTSD | ELFN1 | VTI1A | MRPL3 | KLHL41 | PTPA |  |
| ptd | HIS7 | NR1H4 | HERPUD2 | OR2A25 | COQ4 | MIR33B | AIFM1 |  |
| lsrB | MIP | NDUFAF6 | VWDE | FPR2 | RIPK1 | PLA2G5 | ANPEP |  |
| ARC5A | cgt | VDR | SAYSD1 | ALDH9A1 | G6PC3 | PDE4DIP | RBPJ |  |
| VC_0232 | mutB | AVP | MS4A2 | POMT2 | LTB4R | FAIM | DDAH1 |  |
| AMY1.2 | HN | MT-ATP6 | PAK1 | FIG4 | MIR1246 | INTS8 | STXBP2 |  |
| TPI1 | TT_P0059 | GJA4 | AZGP1 | CD226 | HDAC3 | INTS5 | CTSL |  |
| nrdF | rsuA | MIR214 | NRXN1 | HTR2B | MT2A | ATXN2 | COG6 |  |
| DHFR | mgsA | GJA5 | ATP2B3 | ACP1 | MPP2 | TACR1 | BRAP |  |
| nudE | nprS | IL12A | MOG | ADAMTS9 | GIPC1 | PRKCH | HADHB |  |
| pll | buk2 | ACTN2 | BPGM | LRRK2 | COL12A1 | FOSL1 | MAP3K7 |  |
| Acvr2a | hgprt | DPP4 | KLK4 | TNS1 | MGAM | COL6A6 | ITGA8 |  |
| Art2b | glbN | MIR423 | U2AF1L4 | PYGM | MTAP | MIR384 | MMP10 |  |
| tgt | Fbxo2 | CASP1 | HNRNPDL | ALG3 | MIR23B | LMX1B | SNCA |  |
| chb | bop | CRYAB | MAP3K1 | SKP1 | ESR2 | INF2 | GTF2IRD2 |  |
| xylA | Mbl2 | ANGPT1 | BAG5 | LIG3 | TNFRSF10B | TOR1A | SDHB |  |
| CTSB | pyrE | SORT1 | CFD | ABCG4 | CTSS | SMG6 | EDN3 |  |
| araF | CHI3L1 | MIR34A | SEMA3E | UNC80 | ADPRH | F9 | MPV17 |  |
| eltB |  | CKM | OGDHL | ENPEP | PIEZO2 | PSMC6 | ADA2 |  |
| galM |  | TMEM43 | PPP1R3A | RPL11 | UBR1 | TBXAS1 | GNAI1 |  |
| rpiB |  | ACTB | ASPN | TCOF1 | ATAD3A | SLC2A1 | RELA |  |
| f17aG |  | CDKN2B | ITPR2 | RPL38 | MIR495 | PYCARD | KLF15 |  |
| spoIIAA |  | PIK3CG | PRMT3 | NSF | TRPV2 | RRAS | GAS6 |  |
| TK |  | DZIP1L | XPNPEP1 | RBP3 | DLD | KRT12 | HOTAIR |  |
| YPTB1668 |  | NPY | PABPN1 | CCDC51 | SLIT2 | LAT2 | ACHE |  |
| AMY1A |  | GP1BA | DUX4 | YTHDF1 | PLCB1 | DLX6 | GATAD1 |  |
| fccA |  | ITGB1 | SOCS2 | HSD11B1 | NR0B1 | ARHGEF5 | ADAMTS7 |  |
| ALDOA |  | TEK | KRIT1 | LAMA1 | FZD4 | ABHD11 | PEX6 |  |
| tgtA |  | VIM | KIR2DS2 | AZU1 | ASIC1 | AGBL3 | ABL1 |  |
| udp |  | PROCR | DANCR | EEF1A1 | UBTF | CALN1 | DAAM1 |  |
| phzF |  | CXCL1 | VPS35 | GZMA | MICALL2 | TRIM73 | LOC114827850 |  |
| jgt |  | SELL | GJB4 | CFL2 | YBX1 | CASTOR2 | PHKB |  |
| LDHB |  | CYCS | PCDHGA8 | SLC39A14 | FGFR3 | FHOD1 | DNAJC21 |  |
| yicI |  | HJV | MIR10A | ACOX1 | GPX3 | PCCA | GATA6 |  |
| xynA |  | PHACTR1 | MEF2D | USP24 | NID1 | WFS1 | HLA-DPA1 |  |
| PNPO |  | PLA2G7 | PIK3R1 | RIC8A | CD177 | SGSH | BDNF |  |
| malE |  | ADCY10 | SLC2A2 | ODF3 | GRK4 | GPC4 | IL7 |  |
| CTSK |  | NTS | AMH | SCGB1C1 | MYOG | NDUFA10 | AOC3 |  |
| PRSS1 |  | FGF7 | TGM1 | MIR138-1 | DYSF | SPRED2 | NR1H2 |  |
| BAG1 |  | FLNC | EYA1 | ADAMTS3 | SLC8A3 | WWOX | BAZ1B |  |
| ansB |  | PTGS1 | NIPBL | STX4 | OGN | MCTP2 | GTF2I |  |
| HEXB |  | HSPB1 | A4GALT | RPS3 | KRT14 | HIF1A-AS1 | BUD23 |  |
| mglB |  | KDR | HSD3B1 | ATP5F1B | OPRD1 | MBL1P | NOTCH3 |  |
| BST1 |  | CYP11B2 | ATP1A4 | CAPZA2 | TRAPPC11 | CELF4 | SLCO1B1 |  |
| panC |  | VCP | B3GNT2 | TRIP4 | PSMD9 | GCA | PRKAR1A |  |
| icd |  | ADAM17 | DHX15 | CCL24 | GDF11 | MLKL | CAMK2D |  |
| HSP90AA1 |  | MIR223 | MTMR4 | PLCE1 | MIR503 | GLRX | PGM1 |  |
| IMPA1 |  | GAPDH | GLRX3 | PDE1A | JAG2 | MT-TL2 | ANK2 |  |
| PGK |  | GRK2 | OBSL1 | SCG2 | FUT2 | RPS6KA5 | PCNA |  |
| HPRT |  | MIR140 | CEACAM8 | ARNT | IDH1 | BYSL | FABP1 |  |
| MNS1 |  | SLC9A1 | RPS4X | RPS27 | NFE2 | PPP1R2 | PXDN |  |
| TM_0306 |  | TRPV4 | SHOX | RPL22 | TLL1 | RAD51B | TBL2 |  |
| PH0828 |  | CXCR2 | SHOX2 | PSMD13 | RASA1 | NPFF | FKBP6 |  |
| rhaD |  | A2M | CCDC8 | UBA1 | IL23A | HTR1A | BCL7B |  |
| bglA |  | PAPPA | EGFLAM | PGAM2 | IL7R | PTGFR | METTL27 |  |
| cbh1 |  | PRKG1 | PPM1E | ATG14 | MSTN | SULT2B1 | VPS37D |  |
| actVA 6 |  | MHRT | RXYLT1 | MIR124-2 | EGLN3 | ULK1 | TMEM270 |  |
| lacS |  | IGFBP1 | TRIM23 | ITPA | IRS2 | BAD | BGN |  |
| aroQ |  | BGLAP | SKA2 | BSCL2 | SHC1 | PPP3R1 | CD28 |  |
| AK |  | RYR1 | TRIM17 | ATP8A1 | RPLP0 | ACP3 | IL1F10 |  |
| dxr |  | NOX4 | CNTROB | ST6GALNAC1 | CREBBP | AGO2 | RMND1 |  |
| IGF1R |  | TXN | TRAT1 | PPP1R3B | SRY | ESRRG | MIR146B |  |
| phi |  | MIR150 | PRR11 | CGA | MIR141 | P2RX1 | STAT5A |  |
| pdaA |  | GHRL | RPS4Y1 | IFITM1 | FCN2 | NUDT1 | ADM2 |  |
| B4GALT1 |  | SMARCA4 | TSPY1 | REPS1 | MIR497 | NFIC | IGES |  |
| ispDF |  | TGM2 | URB1 | NNT | RHOD | JPH4 | ATP1B1 |  |
| MUR1 |  | IL9 | MIR127 | SPTBN4 | ANGPTL4 | C1QTNF9B | LAMA2 |  |
| pgl |  | PON2 | MIR376C | MAP3K14 | MFAP4 | MIR19B2 | GPER1 |  |
| rntA |  | PRKACA | MIR487B | MIR224 | MT-ND1 | FUCA1 | BRCA1 |  |
| fumC |  | PPBP | MIR452 | PELATON | AARS2 | GNRHR | FXN |  |
| dhbE |  | CDKN3 | MIR663AHG | HRG | P2RX7 | AP2M1 | PMPCA |  |
| cyp158a2 |  | LPAR1 | CARD10 | TWNK | GHR | EXT2 | PSRC1 |  |
| Grik1 |  | FLT1 | ADAM11 | EXOSC9 | HRH2 | CYB5A | FOSL2 |  |
| ENO1 |  | ANKS6 | ADAMTS8 | TM2D3 | RAD51 | CLDN3 | CD69 |  |
| hmuO |  | TLR3 | NAXE | MIR340 | ZFHX3 | MPZL1 | LEMD2 |  |
| icl |  | GC | FRAS1 | NCOA7 | ERGIC1 | EPN1 | IL22 |  |
| apx1 |  | HBB | ADAMTS20 | HBG1 | PVT1 | RPL6 | IL1R2 |  |
| RHEB |  | MIR208A | APOL4 | SNAP29 | SYP | H3C1 | HBEGF |  |
| uvrB |  | CCL4 | CHADL | EPG5 | TRIB3 | THSD7A | AXL |  |
| TGM2 |  | ADA | EMILIN3 | SMPD2 | CYGB | ABHD10 | RIPK3 |  |
| ISG20 |  | PDGFA | FREM3 | GLP1R | GIGYF2 | TMED2 | MIR27B |  |
| PAE0790 |  | MIR204 | IFI27 | CCNB1 | UCN | LSG1 | TAGLN |  |
| fosA |  | CYP2C9 | TNFRSF10C | TG | PRDX5 | H3C14 | GNAS |  |
| gmhA |  | BPI | TP73 | MAD2L1 | NSUN2 | ZNF713 | CSF3R |  |
| purT |  | HSP90AA1 | ATE1 | ACAD9 | SRSF6 | SSC4D | LOC107133510 |  |
| Pcbd1 |  | UMOD | HEXIM1 | E2F4 | KCNMB1 | POM121C | MYMK |  |
| RHOA |  | PTPRC | ISLR | JUNB | STK11 | SPDYE1 | CTSB |  |
| Grm1 |  | APEX1 | PER1 | MAD2L2 | TPI1 | SPDYE2 | ASPH |  |
| MMP8 |  | ABCG8 | CCDC86 | UGCG | DNM1L | PRSS3P2 | CTSK |  |
| PLAU |  | PDE5A | GLB1 | RNMT | MAD1L1 | SPDYE7P | AOC1 |  |
| TNK2 |  | MAPK8 | AFF4 | NOP2 | TPMT | SPDYE8 | HSD3B7 |  |
| GSK3B |  | CXCR1 | TNFRSF11A | TRMT112 | PIK3C3 | SPDYE10 | MIR199B |  |
| Ahcy |  | P2RY2 | PANX1 | NSUN7 | VPS33A | SPDYE12 | GNAI2 |  |
| PRKACA |  | AHSG | FUT4 | HPD | WNK1 | SPDYE9 | TSC1 |  |
| XOG1 |  | CD163 | SELENOS | RTN4 | NEB | SPDYE14 | CHRM2 |  |
| ppc |  | CXCL2 | CARD9 | PIEZO1 | MIR1262 | SPDYE15 | ROS1 |  |
| Ada |  | SLC25A13 | CUL1 | IDH3A | ASIC3 | SPDYE13 | SLC12A3 |  |
| gpt |  | CLU | MLST8 | CAPZB | RXRA | WBSCR23 | MLYCD |  |
| DPP4 |  | NOD2 | SEPTIN2 | AHNAK | RIPK2 | LAT | MKI67 |  |
| rmlC |  | APOH | STIM1 | LUC7L2 | ETV6 | CKAP4 | COX5A |  |
| lacA |  | TTR | KIR2DL3 | LAIR1 | ITGA5 | MSX1 | CD68 |  |
| cslA |  | LCAT | ANG | ABCB5 | TKT | FBXO7 | IRF1 |  |
| ABD1 |  | SPARC | MYF6 | MRTFB | PEX16 | PRMT1 | MYOCD |  |
| RIB3 |  | LIPA | FRG1 | LMF1 | HRH1 | RALBP1 | ERBB4 |  |
| queA |  | MT-CYB | ABCA13 | SORL1 | ART1 | FGF14 | EDN2 |  |
| trpD |  | CYBA | FOXA1 | KCNK1 | SOST | SYTL4 | IFNB1 |  |
| ams |  | THBS2 | BNIP2 | FAM167A | PKP3 | ELF5 | ABCG1 |  |
| aly |  | EPRS1 | TSC22D4 | CFHR2 | ACVR1 | OSTN | MIR182 |  |
| cel3 |  | LTF | INSIG1 | RORA | AGFG1 | STX2 | IL13RA1 |  |
| TRI5 |  | BAG3 | HS3ST1 | IRF9 | MIRLET7I | GALNT4 | CA2 |  |
| ykoF |  | RHOA | PKN1 | PEX7 | NOTCH2NLC | COL9A3 | P2RY6 |  |
| aguA |  | CDH2 | XRCC2 | POMK | ANO5 | AP5Z1 | LGALS1 |  |
| AMY2A |  | DTNA | FOXF1 | PPP1CB | STK4 | KCNH7 | GLI3 |  |
| hisG |  | HSPG2 | TNNT3 | PFDN4 | AHCY | FMN1 | MIR16-1 |  |
| CHI1 |  | TSLP | LNCARSR | FGFBP2 | HSPA1L | MTARC1 | XRCC5 |  |
| glmS |  | CCL22 | KIR2DS1 | CTTNBP2 | GARS1 | GLCE | KCNJ18 |  |
| CPB1 |  | MIR133A1 | TYRO3 | CPM | MIR203A | NNMT | DAG1 |  |
| DPYD |  | TUBB1 | MS4A1 | CDC42 | HEY2 | KIR2DL4 | ROCK1 |  |
| fucI |  | TNNT1 | PRKAG1 | SLC5A6 | RIT1 | COL13A1 | MIR181A1 |  |
| DCK |  | MTOR | SNRK | GAB1 | TERF2 | COASY | NOX5 |  |
| OTC |  | TOLLIP | PPY | GGCX | ATP2A1 | CDK20 | FST |  |
| HINT1 |  | XBP1 | NEFH | OAT | MIR361 | PARP3 | HARS1 |  |
| fcl |  | PPARGC1A | MATR3 | MRE11 | NBN | CCDC22 | PON3 |  |
| pobA |  | CCR4 | DNM3OS | LIPF | P4HB | IK | CACNA2D1 |  |
| PDPK1 |  | EPHX1 | MIR1202 | SBF1 | JMJD6 | VPS4B | PARK7 |  |
| At4g18930 |  | IL6R | GNPTAB | KLRK1 | MIR96 | PARP10 | FGF19 |  |
| TGM3 |  | MIR122 | ATP5F1D | GALNT17 | NAGA | POPDC2 | S1PR1 |  |
| GPI |  | AP3B1 | ESRRA | CDK9 | PEX2 | PAMR1 | GPX1 |  |
| KCNMB2 |  | CYP1A2 | GADD45A | TREX1 | ATF2 | USP7 | LOC106099062 |  |
| ansB |  | PNPLA3 | HAS3 | PRDX3 | CALM3 | CREM | HGD |  |
| AKR1B1 |  | CXCL9 | BAIAP2L1 | MIR196A2 | MIR19A | VOPP1 | FLNA |  |
| MC1 |  | ACD | TMEM168 | USF2 | BLM | MAML2 | CPT2 |  |
| def |  | CHKA | CLDN4 | SGPP1 | SLC5A1 | FGL2 | APOL1 |  |
| gltX |  | MYDGF | SLC6A3 | HTT | BLOC1S4 | SYNPO2L | HAVCR1 |  |
| AKR1C3 |  | PHKG2 | SLC29A1 | BMPR1B | ATR | MEG8 | TFAM |  |
| HIS7 |  | PPA2 | GORASP1 | SEPTIN9 | CCL8 | EFEMP1 | SHROOM3 |  |
| MIP |  | HFE-AS1 | TAC3 | TRPC4 | STMN2 | KCNK2 | ALG2 |  |
| cgt |  | S100A12 | PPP2R5C | MIR488 | NCAM1 | HINT1 | MRPS22 |  |
| HN |  | PALLD | ANKH | ENHO | VEGFB | KCNA1 | ASCL1 |  |
| rsuA |  | TF | TOMM40 | TFG | MIR103A1 | CNBP | EFCAB13 |  |
| mgsA |  | S100A4 | MMUT | DLL1 | FOXP2 | B4GAT1 | PVALB |  |
| nprS |  | APOA4 | TKFC | MIR25 | ASCC1 | ACYP2 | DAXX |  |
| buk2 |  | CKB | MPZ | GFI1 | SMAD1 | SGCE | BAZ1A |  |
| hgprt |  | ANO1 | SUOX | RGS19 | EIF2AK2 | UBE2H | ADAM22 |  |
| glbN |  | ABCC1 | POGLUT1 | CKMT1B | PTPN1 | CILP | CDON |  |
| Fbxo2 |  | FGFR1 | FKBP14 | FANCM | CYP11A1 | EPHA1 | PTTG1 |  |
| PROK |  | ENPP2 | C19orf12 | MIR545 | PTN | SMANTIS | MTFR1 |  |
| Mbl2 |  | CTLA4 | PHOX2B | PYY | LGALS9 | TRPC1 | OCRL |  |
| GLAA |  | GP6 | ORMDL3 | RASSF1 | APP | GAD1 | GMPR |  |
| cel6A |  | MIR133B | GPRC5B | GLRX2 | EIF2S1 | SNAP25 | BLVRB |  |
| CIP1 |  | SCARB1 | ROR1 | LRMDA | LONP1 | HNRNPK | PI4KB |  |
| gyrB |  | MIR146A | KLK2 | PGRMC1 | HLA-E | TNPO1 | EXOSC5 |  |
| acpS |  | UTS2 | AGPAT1 | ARID3A | PTGER2 | RENBP | DOCK9 |  |
| PAP1 |  | APOA5 | MANF | ARHGEF3 | F2RL2 | MZF1 | SASH1 |  |
| GRESAG 4.1 |  | ATF6 | NEUROD1 | MIR16-2 | MIR296 | TOMM7 | SBF2 |  |
| mutY |  | HSPA5 | NHLRC1 | SUGCT | PLOD1 | RBM24 | ARID4A |  |
| pyrE |  | MMP13 | TSC22D1 | CBX5 | ALDOA | STRIP2 | HLF |  |
| gaI |  | DSC2 | HES1 | CD276 | IKBKB | ART3 | DDX41 |  |
| CHI3L1 |  | HCN4 | RHOB | HTN3 | EXT1 | SIRPA | RPS10 |  |
| bop |  | VCL | C4BPB | COQ6 | EMILIN1 | C1QA | RPL26 |  |
| ohr |  | XDH | CCRL2 | POLR1D | SRPRA | SOX4 | RPS17 |  |
| BCHE |  | SP1 | ADIPOR1 | ADAMTS2 | MFAP5 | DUSP6 | RPS20 |  |
| estF |  | TLR9 | SERPINI1 | GSDMA | RPS6KA3 | PTPRN2 | RPS24 |  |
| MTAP |  | IL12B | CCL13 | DNM1 | SCO2 | EIF2AK1 | RPS26 |  |
| serA |  | ACP5 | HOXA4 | QRSL1 | ALPP | VIPR2 | RPS29 |  |
| mutB |  | CTSG | ANGPTL8 | USF1 | WNK4 | TAF4 | MIR139 |  |
| MSDC |  | HTR2A | UCMA | CIC | SDHD | UGGT2 | KL |  |
| algD |  | CCN4 | FENDRR | SH3PXD2A | OTULIN | ITM2C | PHKA2 |  |
| WBAI |  | KRT8 | EFNB2 | SIGIRR | IRAK1 | RABGAP1L | SKIC2 |  |
| PPID |  | MMP12 | MAOB | DEAF1 | MYOM3 | EMC8 | AP3D1 |  |
| AVD |  | GPD1 | C1QTNF9 | PGGHG | GPC3 | L1CAM | TCAP |  |
| galK |  | ADD1 | LPIN1 | ZNF318 | PEPD | SNX10 | ADK |  |
| rsbQ |  | STAT4 | CDK5 | ZNF526 | ZFYVE9 | ORM2 | ABCF2 |  |
| dhlA |  | CYP3A4 | DLG1 | IFITM2 | ZEB2 | TNFSF13 | VASP |  |
| uaZ |  | FBN1 | SNTB2 | TMEM91 | USP4 | NAB2 | ST2 |  |
| budC |  | NOTCH1 | RNF213 | STRIP1 | CCL21 | INSL3 | ACTA1 |  |
| ampC |  | CD80 | ANXA8 | ZNF574 | MYL9 | MEOX2 | CCR1 |  |
| HSPA8 |  | BMP2 | ERG | CCDC97 | LCN1 | SMARCD3 | CXCL16 |  |
| CA12 |  | SLC6A4 | RTN1 | PRPF38B | SQOR | SCRN1 | PTGIR |  |
| thyA |  | APOA2 | RLN3 | SPC24 | KCND2 | ASB2 | EPAS1 |  |
| xapA |  | NPPC | GPC1 | ANKRD31 | RPL15 | OSBPL3 | PLA2G6 |  |
| uprt |  | SLPI | UFM1 | LRRC37A2 | RPL35A | TRIM44 | EGR1 |  |
| tthHB8IM |  | VDAC1 | UFL1 | TMEM80 | RPS15 | MACROD2 | ADRA2B |  |
| araC |  | MT-ND2 | PPP1R17 | TMEM61 | F2RL3 | FAM114A1 | UTP4 |  |
| RNASE1 |  | CANX | MON1B | RPRML | ETS1 | HSBP1 | PLAUR |  |
| Itpr1 |  | CTF1 | SLC22A2 | TDRD15 | HSF1 | OCM2 | EPX |  |
| Mbl1 |  | SERPINB1 | PRRT2 | CYP2G1P | SYNE2 | HSPA14 | GBE1 |  |
| YML079W |  | CDH5 | ESCO2 | MIR936 | LOC110806262 | SNX3 | AKAP1 |  |
| PYGM |  | CIITA | PLCL1 | MIR609 | SETX | ARHGEF2 | IL2RB |  |
| abfA |  | GSR | MYLK3 | RPL17P7 | MIR188 | COL28A1 | ICOSLG |  |
| LTP1 |  | MIR29B1 | PPP1R1A | ENSG00000262633 | PKM | STARD3NL | CCK |  |
| kaiB1 |  | CPB2 | DNAJB9 | ENSG00000269843 | NLRC5 | HLA-DRB4 | DEFA1 |  |
| HA |  | CSRP3 | TSPYL2 | RPL7P8 | CHRNA7 | HLA-S | DUSP5 |  |
| Syt1 |  | PGF | ST3GAL4 | ENSG00000258634 | XYLT2 | HTR1D | RAPGEF2 |  |
| glkA |  | DNAJC5 | CD99 | ENSG00000254559 | TSPO | FKBP4 | AKR7A2 |  |
| TGFBR2 |  | MYL4 | MIR135B | ENSG00000262879 | GPR161 | HTR2C | MAP4 |  |
| folK |  | S100B | ADCY9 | ENSG00000289744 | OXA1L | HNRNPD | MTDH |  |
| gdhB |  | XPNPEP3 | LTB4R2 | lnc-MYBPHL-1 | COQ8B | NPTX1 | NLN |  |
| rbsB |  | BECN1 | CASD1 | lnc-SPC24-1 | DHCR24 | HTATIP2 | USP18 |  |
| suhB |  | CMA1 | RPS16 | NONHSAG001562.2 | CCDC174 | SLC16A4 | ARHGAP35 |  |
| ctxB |  | MGP | ABCB6 | lnc-STN1-1 | PPP1R12A | USO1 | MBNL1 |  |
| cmoJ |  | STUB1 | ING2 | lnc-ZNF296-6 | LRP5 | GBP5 | OSTF1 |  |
| cbh2 |  | MYD88 | SLC7A8 | piR-52364 | CPQ | RNF145 | PPP1R14A |  |
| alpha-Man-IIa |  | BRAF | CERKL | piR-56320 | GALNTL5 | SHTN1 | RBBP5 |  |
| nrdD |  | PDP1 | TMEM39A | piR-60314 | DIS3L | ENDOD1 | TOMM70 |  |
| RSc3288 |  | BDKRB2 | GGPS1 | CM034960-323 | NR4A3 | MXRA7 | TPSB2 |  |
| CDK2 |  | MET | DMPK | piR-52932 | EEF2 | MIR502 | TMX1 |  |
| PYGL |  | MAPK1 | DKK2 | NONHSAG026010.2 | LMNB2 | GALNT2 | TNFSF9 |  |
| accC |  | CAMP | FGF8 | SLC20A2 | SFRP1 | HSPA1A | YTHDC2 |  |
| FCAR |  | F8 | SASH3 | ITPK1 | ATF3 | SLC26A4 | LSM14A |  |
| Crat |  | IL2RA | HOXA5 | METTL3 | LAMP3 | IFIH1 | PTDSS2 |  |
| crp |  | PRKAA2 | BAG6 | FMR1 | MLC1 | KITLG | TM9SF4 |  |
| mtnN |  | ANXA2 | MTA1 | MIR215 | PZP | PLA2G2A | TFPT |  |
| PNP |  | RBP4 | FKTN | PSMC3 | SERPING1 | PSMB4 | SPP2 |  |
| GGTA1 |  | TLR5 | BSND | CD8A | MT-ATP8 | SPRY3 | KIAA0232 |  |
| SOT12 |  | RAB11B | ADH1C | THY1 | IFNGR1 | SHH | SBSPON |  |
| axe1 |  | IFNA1 | FHIT | PSMC4 | DMBT1 | MIR195 | RETREG2 |  |
| comB |  | MIR185 | SMARCAD1 | PSMA7 | GAA | ITGA3 | DEFA1B |  |
| folP |  | ENTPD1 | STING1 | PSMB8 | ITGAL | TRIM37 | LOC111365141 |  |
| folB |  | SHOC2 | ARSB | CACNA1C | FGF1 | MIR342 | ST13 |  |
| kdsA |  | ABO | KIF20A | PRSS8 | MIR1-2 | UBC | SLC30A5 |  |
| trpA |  | GUSB | RPL5 | GZMB | PRKG2 | MIR455 | KMT2C |  |
| nagB |  | PRDM16 | CCR7 | C1S | TGFA | COL4A1 | WDR83OS |  |
| ppgmk |  | MYH9 | SLC26A8 | CSK | LPO | B2M | CARD8 |  |
| purA |  | FURIN | EPCAM | JAK1 | GBA1 | FAP | SIGLEC1 |  |
| GLU1 |  | MED12 | TFR2 | FIP1L1 | SCN4A | CCND1 | ENSG00000195401 |  |
| cobT |  | SOCS3 | LAMP1 | HLA-A | MST1 | IL12RB1 | CDK5RAP3 |  |
| rbsK |  | PMM2 | FGF23 | PSMA3 | LOC102723566 | IGF2 | NAA10 |  |
| PAH |  | KIAA0586 | VHL | PI3 | TWIST1 | IGHE | TAT |  |
| Nos1 |  | IL6ST | CHUK | CASR | MEF2C | GPR35 | GHRH |  |
| ANG |  | TNNC1 | C4A | MDM2 | IL1RL1 | RAF1 | SLC17A1 |  |
| rho |  | ARL13B | PARP1 | SELPLG | PDCD1 | SP3 | NT5C1B |  |
| nlpI |  | NF2 | EPOR | PDE3A | FKBP1A | IL3 | HMGA1 |  |
| glcB |  | GSTT1 | ATP12A | HLA-DPB1 | FLT4 | CD274 | MAP3K11 |  |
| NA |  | IFT74 | OGDH | MIR144 | FOXE3 | RAB5A | ELOVL5 |  |
| metB |  | ACADVL | IL20 | HDAC2 | BCL2A1 | MYPN | PDLIM7 |  |
| HK1 |  | REG3A | STAP1 | PDZK1 | HIF3A | H19 | SH2B1 |  |
| DHA2_112885 |  | HLA-DQA1 | SLC5A5 | RAPGEF3 | KAT5 | TIMP3 | RND3 |  |
| MDL1 |  | INSR | MUC16 | KCNJ1 | ADAM9 | BCHE | ATG12 |  |
| ldha |  | PLAU | UQCRC1 | CH25H | PTPN12 | IDH2 | DRAM2 |  |
| SRC |  | EZR | ATP1A2 | S1PR3 | GSS | STAT5B | FASTK |  |
| YMR087W |  | EMC10 | FADS1 | PIK3R4 | BCAT1 | MLXIPL | GSTCD |  |
| CEL7B |  | ATP2A2 | FOXL1 | ELOB | ETV1 | EDNRB | PML |  |
| PIM1 |  | HLA-DQB1 | KCTD1 | CALB2 | MMAB | FGF10 | RNASEH2C |  |
| fucA |  | CREB1 | LIN28A | KANTR | EIF2B5 | PSMA2 | SPI1 |  |
| thiD |  | NPR1 | LINC00636 | BCAR1 | NRF1 | TAC1 | DACT1 |  |
| ispF |  | ERN1 | MIR450A2 | MIR124-3 | RABGEF1 | APC | VTI1B |  |
| spoII |  | MIR132 | EEF2K | CYP1B1 | LAG3 | KRT13 | STAR |  |
| FCY1 |  | IARS1 | MYBL2 | SCAMP2 | DOCK4 | CLIP2 | SUCLA2 |  |
| rbsD |  | CYBB | ANGEL1 | CDK2 | PPP2R5E | DPM1 | TMEM165 |  |
| SP_0314 |  | MTR | SDHC | SAMHD1 | NRG4 | CYP2J2 | EIF2AK4 |  |
| udg |  | PTEN | SDHAF2 | BNIP3 | A1BG | MIR221 | PYGL |  |
| ptd |  | GJC1 | TMEM127 | PTGDS | ABI3BP | CERS2 | FOS |  |
| lsrB |  | CLCN3 | SLC22A12 | S100A11 | PDAP1 | SIX1 | SERPINB2 |  |
| ARC5A |  | SAA1 | VSIG4 | PCBP2 | ANKIB1 | CLASP1 | ILK |  |
| VC_0232 |  | SGK1 | ANXA6 | LYVE1 | CD55 | WNK2 | MIR34C |  |
| AMY1.2 |  | KCNJ2 | CD59 | MYH4 | BSG | FBXO8 | ADRB3 |  |
| TPI1 |  | CNR1 | LMOD2 | CLEC4A | MAPK13 | TIMM23 | KCNE3 |  |
| nrdF |  | CLCN2 | ADCYAP1 | ABCF1 | KDM4B | CRPPA | LAMC2 |  |
| DHFR |  | PYGB | LRP1 | MYOZ2 | ADCYAP1R1 | SLC12A1 | KIT |  |
| nudE |  | ANXA1 | DMD | MYL12A | MARK2 | LITAF | TM6SF2 |  |
| pll |  | PSMC5 | HSPE1 | BHLHE40 | NDRG1 | DSTYK | NAMPT |  |
| Acvr2a |  | GRP | MTTP | ITCH | OSMR | CABLES1 | TBX4 |  |
| Art2b |  | SHBG | MIR19B1 | HDAC6 | RBL2 | RBX1 | MIR335 |  |
| tgt |  | BBS2 | NPR2 | PROK2 | TRAF3IP2 | EGLN1 | TSC2 |  |
| chb |  | MIR143 | TCF4 | LTB | CTTN | TRIM31 | PTPN22 |  |
| xylA |  | SLC4A4 | ABCB7 | PLA2G4A | GNAL | DRD4 | BAX |  |
| CTSB |  | LTA4H | CYP7A1 | TNC | TIRAP | BRIP1 | IFT27 |  |
| araF |  | KCNH2 | CYP1A1 | PDE4D | BNIP3L | FANCB | TUBB |  |
| eltB |  | SMPD1 | TNFRSF1B | DDIT3 | SOX9 | BCR | SULT1A3 |  |
| galM |  | NFKB1 | KCNK3 | H2AC18 | SUFU | PTGES3 | PPIG |  |
| rpiB |  | RPS27A | TRDN | ACVRL1 | MIR22 | ERBIN | CDKN1A |  |
| f17aG |  | CTNNA3 | NR3C1 | VTN | PNLIP | PCSK2 | COG2 |  |
| spoIIAA |  | MME | SEMA4D | HNF1A | FABP12 | PSMA4 | CSF1 |  |
| TK |  | RPL36A-HNRNPH2 | MIRLET7G | BMP1 | NAT2 | PSMD4 | MIR192 |  |
| YPTB1668 |  | MEFV | DPYD | MIR212 | AKAP13 | MIR125B1 | TREM1 |  |

**Table 3** Results of Gene Ontology (GO) and Kyoto Encyclopedia of Genes and Genomes (KEGG) Enrichment analysis

| **Ontology** | **ID** | **Description** | **P value** | **p. adjust** | **Q value** |
| --- | --- | --- | --- | --- | --- |
| BP | GO:0072521 | purine-containing compound metabolic process | 1.83E-10 | 5.00E-07 | 3.10E-07 |
| BP | GO:0036293 | response to decreased oxygen levels | 5.78E-10 | 7.70E-07 | 4.77E-07 |
| BP | GO:0033674 | positive regulation of kinase activity | 1.06E-09 | 7.70E-07 | 4.77E-07 |
| BP | GO:0019693 | ribose phosphate metabolic process | 1.13E-09 | 7.70E-07 | 4.77E-07 |
| BP | GO:0070482 | response to oxygen levels | 1.44E-09 | 7.87E-07 | 4.88E-07 |
| BP | GO:0001666 | response to hypoxia | 5.03E-09 | 2.30E-06 | 1.42E-06 |
| BP | GO:0030574 | collagen catabolic process | 7.73E-09 | 2.85E-06 | 1.76E-06 |
| BP | GO:2001234 | negative regulation of apoptotic signaling pathway | 8.33E-09 | 2.85E-06 | 1.76E-06 |
| BP | GO:0009259 | ribonucleotide metabolic process | 9.69E-09 | 2.95E-06 | 1.83E-06 |
| BP | GO:0006163 | purine nucleotide metabolic process | 1.29E-08 | 3.39E-06 | 2.10E-06 |
| BP | GO:0009117 | nucleotide metabolic process | 1.43E-08 | 3.39E-06 | 2.10E-06 |
| BP | GO:1901652 | response to peptide | 1.57E-08 | 3.39E-06 | 2.10E-06 |
| BP | GO:0006091 | generation of precursor metabolites and energy | 1.69E-08 | 3.39E-06 | 2.10E-06 |
| BP | GO:0006753 | nucleoside phosphate metabolic process | 1.73E-08 | 3.39E-06 | 2.10E-06 |
| BP | GO:0009150 | purine ribonucleotide metabolic process | 6.35E-08 | 1.14E-05 | 7.07E-06 |
| BP | GO:0032963 | collagen metabolic process | 6.99E-08 | 1.14E-05 | 7.07E-06 |
| BP | GO:2001233 | regulation of apoptotic signaling pathway | 7.09E-08 | 1.14E-05 | 7.07E-06 |
| BP | GO:0071375 | cellular response to peptide hormone stimulus | 8.48E-08 | 1.23E-05 | 7.65E-06 |
| BP | GO:0022617 | extracellular matrix disassembly | 8.57E-08 | 1.23E-05 | 7.65E-06 |
| BP | GO:0043434 | response to peptide hormone | 1.72E-07 | 2.35E-05 | 1.46E-05 |
| BP | GO:0019318 | hexose metabolic process | 1.87E-07 | 2.43E-05 | 1.51E-05 |
| BP | GO:0018209 | peptidyl-serine modification | 3.09E-07 | 3.84E-05 | 2.38E-05 |
| BP | GO:0005996 | monosaccharide metabolic process | 3.54E-07 | 4.21E-05 | 2.61E-05 |
| BP | GO:1903829 | positive regulation of protein localization | 4.60E-07 | 5.25E-05 | 3.25E-05 |
| BP | GO:1901653 | cellular response to peptide | 5.64E-07 | 6.18E-05 | 3.83E-05 |
| BP | GO:0032869 | cellular response to insulin stimulus | 6.55E-07 | 6.90E-05 | 4.27E-05 |
| BP | GO:2000377 | regulation of reactive oxygen species metabolic process | 9.10E-07 | 9.22E-05 | 5.71E-05 |
| BP | GO:0016052 | carbohydrate catabolic process | 1.14E-06 | 1.09E-04 | 6.75E-05 |
| BP | GO:0097191 | extrinsic apoptotic signaling pathway | 1.15E-06 | 1.09E-04 | 6.75E-05 |
| BP | GO:2001237 | negative regulation of extrinsic apoptotic signaling pathway | 1.25E-06 | 1.12E-04 | 6.92E-05 |
| BP | GO:0046777 | protein autophosphorylation | 1.28E-06 | 1.12E-04 | 6.92E-05 |
| BP | GO:0045860 | positive regulation of protein kinase activity | 1.31E-06 | 1.12E-04 | 6.92E-05 |
| BP | GO:1904645 | response to amyloid-beta | 1.50E-06 | 1.24E-04 | 7.69E-05 |
| BP | GO:0072593 | reactive oxygen species metabolic process | 1.61E-06 | 1.27E-04 | 7.86E-05 |
| BP | GO:2001243 | negative regulation of intrinsic apoptotic signaling pathway | 1.68E-06 | 1.27E-04 | 7.86E-05 |
| BP | GO:0018105 | peptidyl-serine phosphorylation | 1.69E-06 | 1.27E-04 | 7.86E-05 |
| BP | GO:0033002 | muscle cell proliferation | 1.72E-06 | 1.27E-04 | 7.86E-05 |
| BP | GO:0097305 | response to alcohol | 1.83E-06 | 1.32E-04 | 8.16E-05 |
| BP | GO:0016485 | protein processing | 2.35E-06 | 1.65E-04 | 1.02E-04 |
| BP | GO:0048660 | regulation of smooth muscle cell proliferation | 2.41E-06 | 1.65E-04 | 1.02E-04 |
| BP | GO:2001242 | regulation of intrinsic apoptotic signaling pathway | 2.50E-06 | 1.67E-04 | 1.04E-04 |
| BP | GO:0042060 | wound healing | 2.68E-06 | 1.72E-04 | 1.07E-04 |
| BP | GO:0048659 | smooth muscle cell proliferation | 2.70E-06 | 1.72E-04 | 1.07E-04 |
| BP | GO:1902175 | regulation of oxidative stress-induced intrinsic apoptotic  signaling pathway | 3.27E-06 | 1.99E-04 | 1.23E-04 |
| BP | GO:0045936 | negative regulation of phosphate metabolic process | 3.35E-06 | 1.99E-04 | 1.23E-04 |
| BP | GO:0018107 | peptidyl-threonine phosphorylation | 3.38E-06 | 1.99E-04 | 1.23E-04 |
| BP | GO:0010563 | negative regulation of phosphorus metabolic process | 3.42E-06 | 1.99E-04 | 1.23E-04 |
| BP | GO:0032868 | response to insulin | 3.76E-06 | 2.15E-04 | 1.33E-04 |
| BP | GO:0032148 | activation of protein kinase B activity | 4.31E-06 | 2.41E-04 | 1.49E-04 |
| BP | GO:0001952 | regulation of cell-matrix adhesion | 5.00E-06 | 2.73E-04 | 1.69E-04 |
| BP | GO:0018210 | peptidyl-threonine modification | 5.24E-06 | 2.77E-04 | 1.72E-04 |
| BP | GO:0051881 | regulation of mitochondrial membrane potential | 5.36E-06 | 2.77E-04 | 1.72E-04 |
| BP | GO:2000379 | positive regulation of reactive oxygen species metabolic process | 5.36E-06 | 2.77E-04 | 1.72E-04 |
| BP | GO:0046034 | ATP metabolic process | 5.55E-06 | 2.81E-04 | 1.74E-04 |
| BP | GO:0031334 | positive regulation of protein-containing complex assembly | 5.76E-06 | 2.87E-04 | 1.78E-04 |
| BP | GO:0006809 | nitric oxide biosynthetic process | 7.03E-06 | 3.43E-04 | 2.13E-04 |
| BP | GO:1900180 | regulation of protein localization to nucleus | 7.51E-06 | 3.60E-04 | 2.23E-04 |
| BP | GO:0002064 | epithelial cell development | 7.76E-06 | 3.66E-04 | 2.27E-04 |
| BP | GO:1903201 | regulation of oxidative stress-induced cell death | 8.00E-06 | 3.70E-04 | 2.30E-04 |
| BP | GO:0050878 | regulation of body fluid levels | 8.12E-06 | 3.70E-04 | 2.30E-04 |
| BP | GO:0097193 | intrinsic apoptotic signaling pathway | 9.77E-06 | 4.38E-04 | 2.72E-04 |
| BP | GO:0006096 | glycolytic process | 1.03E-05 | 4.46E-04 | 2.76E-04 |
| BP | GO:0046209 | nitric oxide metabolic process | 1.03E-05 | 4.46E-04 | 2.76E-04 |
| BP | GO:0006757 | ATP generation from ADP | 1.09E-05 | 4.59E-04 | 2.84E-04 |
| BP | GO:2001057 | reactive nitrogen species metabolic process | 1.09E-05 | 4.59E-04 | 2.84E-04 |
| BP | GO:0032147 | activation of protein kinase activity | 1.14E-05 | 4.73E-04 | 2.93E-04 |
| BP | GO:0043491 | protein kinase B signaling | 1.16E-05 | 4.75E-04 | 2.94E-04 |
| BP | GO:0043254 | regulation of protein-containing complex assembly | 1.20E-05 | 4.82E-04 | 2.99E-04 |
| BP | GO:0045429 | positive regulation of nitric oxide biosynthetic process | 1.35E-05 | 5.36E-04 | 3.32E-04 |
| BP | GO:0051604 | protein maturation | 1.54E-05 | 6.00E-04 | 3.72E-04 |
| BP | GO:1904407 | positive regulation of nitric oxide metabolic process | 1.64E-05 | 6.31E-04 | 3.91E-04 |
| BP | GO:0046031 | ADP metabolic process | 1.72E-05 | 6.53E-04 | 4.05E-04 |
| BP | GO:2001236 | regulation of extrinsic apoptotic signaling pathway | 1.75E-05 | 6.54E-04 | 4.05E-04 |
| BP | GO:0002066 | columnar/cuboidal epithelial cell development | 1.80E-05 | 6.64E-04 | 4.12E-04 |
| BP | GO:1900407 | regulation of cellular response to oxidative stress | 1.91E-05 | 6.97E-04 | 4.32E-04 |
| BP | GO:0008631 | intrinsic apoptotic signaling pathway in response to oxidative stress | 1.97E-05 | 7.08E-04 | 4.39E-04 |
| BP | GO:0009261 | ribonucleotide catabolic process | 2.15E-05 | 7.63E-04 | 4.73E-04 |
| BP | GO:0062197 | cellular response to chemical stress | 2.29E-05 | 8.04E-04 | 4.98E-04 |
| BP | GO:0003044 | regulation of systemic arterial blood pressure mediated by  a chemical signal | 2.55E-05 | 8.82E-04 | 5.47E-04 |
| BP | GO:0036473 | cell death in response to oxidative stress | 2.60E-05 | 8.88E-04 | 5.50E-04 |
| BP | GO:0006165 | nucleoside diphosphate phosphorylation | 2.73E-05 | 9.14E-04 | 5.67E-04 |
| BP | GO:0031667 | response to nutrient levels | 2.77E-05 | 9.14E-04 | 5.67E-04 |
| BP | GO:0045785 | positive regulation of cell adhesion | 2.77E-05 | 9.14E-04 | 5.67E-04 |
| BP | GO:0071732 | cellular response to nitric oxide | 2.84E-05 | 9.22E-04 | 5.71E-04 |
| BP | GO:0046939 | nucleotide phosphorylation | 2.86E-05 | 9.22E-04 | 5.71E-04 |
| BP | GO:0072523 | purine-containing compound catabolic process | 3.00E-05 | 9.41E-04 | 5.83E-04 |
| BP | GO:1902882 | regulation of response to oxidative stress | 3.00E-05 | 9.41E-04 | 5.83E-04 |
| BP | GO:0051924 | regulation of calcium ion transport | 3.06E-05 | 9.41E-04 | 5.83E-04 |
| BP | GO:1901136 | carbohydrate derivative catabolic process | 3.06E-05 | 9.41E-04 | 5.83E-04 |
| BP | GO:0009135 | purine nucleoside diphosphate metabolic process | 3.30E-05 | 9.93E-04 | 6.15E-04 |
| BP | GO:0009179 | purine ribonucleoside diphosphate metabolic process | 3.30E-05 | 9.93E-04 | 6.15E-04 |
| BP | GO:0022411 | cellular component disassembly | 3.53E-05 | 1.05E-03 | 6.51E-04 |
| BP | GO:0010634 | positive regulation of epithelial cell migration | 3.61E-05 | 1.05E-03 | 6.53E-04 |
| BP | GO:0008585 | female gonad development | 3.62E-05 | 1.05E-03 | 6.53E-04 |
| BP | GO:0006090 | pyruvate metabolic process | 3.79E-05 | 1.09E-03 | 6.77E-04 |
| BP | GO:0010631 | epithelial cell migration | 3.92E-05 | 1.12E-03 | 6.92E-04 |
| BP | GO:0051900 | regulation of mitochondrial depolarization | 4.02E-05 | 1.12E-03 | 6.96E-04 |
| BP | GO:1902170 | cellular response to reactive nitrogen species | 4.02E-05 | 1.12E-03 | 6.96E-04 |
| BP | GO:0051054 | positive regulation of DNA metabolic process | 4.12E-05 | 1.14E-03 | 7.05E-04 |
| BP | GO:0090132 | epithelium migration | 4.16E-05 | 1.14E-03 | 7.05E-04 |
| BP | GO:0030324 | lung development | 4.22E-05 | 1.14E-03 | 7.09E-04 |
| BP | GO:0009408 | response to heat | 4.33E-05 | 1.15E-03 | 7.11E-04 |
| BP | GO:0046545 | development of primary female sexual characteristics | 4.33E-05 | 1.15E-03 | 7.11E-04 |
| BP | GO:0009266 | response to temperature stimulus | 4.36E-05 | 1.15E-03 | 7.11E-04 |
| BP | GO:0090130 | tissue migration | 4.58E-05 | 1.19E-03 | 7.40E-04 |
| BP | GO:1902176 | negative regulation of oxidative stress-induced intrinsic  apoptotic signaling pathway | 4.72E-05 | 1.20E-03 | 7.44E-04 |
| BP | GO:0030323 | respiratory tube development | 4.78E-05 | 1.20E-03 | 7.44E-04 |
| BP | GO:0045766 | positive regulation of angiogenesis | 4.78E-05 | 1.20E-03 | 7.44E-04 |
| BP | GO:1904018 | positive regulation of vasculature development | 4.78E-05 | 1.20E-03 | 7.44E-04 |
| BP | GO:0009991 | response to extracellular stimulus | 4.84E-05 | 1.20E-03 | 7.45E-04 |
| BP | GO:0009185 | ribonucleoside diphosphate metabolic process | 4.94E-05 | 1.22E-03 | 7.54E-04 |
| BP | GO:0001541 | ovarian follicle development | 5.05E-05 | 1.23E-03 | 7.64E-04 |
| BP | GO:0038083 | peptidyl-tyrosine autophosphorylation | 5.50E-05 | 1.29E-03 | 8.02E-04 |
| BP | GO:0045056 | transcytosis | 5.50E-05 | 1.29E-03 | 8.02E-04 |
| BP | GO:0051882 | mitochondrial depolarization | 5.50E-05 | 1.29E-03 | 8.02E-04 |
| BP | GO:0071731 | response to nitric oxide | 5.50E-05 | 1.29E-03 | 8.02E-04 |
| BP | GO:0044282 | small molecule catabolic process | 5.54E-05 | 1.29E-03 | 8.02E-04 |
| BP | GO:0044089 | positive regulation of cellular component biogenesis | 6.13E-05 | 1.41E-03 | 8.74E-04 |
| BP | GO:1901361 | organic cyclic compound catabolic process | 6.13E-05 | 1.41E-03 | 8.74E-04 |
| BP | GO:0008286 | insulin receptor signaling pathway | 6.33E-05 | 1.44E-03 | 8.95E-04 |
| BP | GO:0045428 | regulation of nitric oxide biosynthetic process | 6.60E-05 | 1.49E-03 | 9.25E-04 |
| BP | GO:0034599 | cellular response to oxidative stress | 6.68E-05 | 1.50E-03 | 9.29E-04 |
| BP | GO:0044262 | cellular carbohydrate metabolic process | 7.14E-05 | 1.59E-03 | 9.84E-04 |
| BP | GO:0003323 | type B pancreatic cell development | 7.28E-05 | 1.61E-03 | 9.96E-04 |
| BP | GO:0010632 | regulation of epithelial cell migration | 7.62E-05 | 1.67E-03 | 1.03E-03 |
| BP | GO:0080164 | regulation of nitric oxide metabolic process | 7.97E-05 | 1.72E-03 | 1.07E-03 |
| BP | GO:0046660 | female sex differentiation | 8.02E-05 | 1.72E-03 | 1.07E-03 |
| BP | GO:0006006 | glucose metabolic process | 8.04E-05 | 1.72E-03 | 1.07E-03 |
| BP | GO:0072522 | purine-containing compound biosynthetic process | 8.27E-05 | 1.75E-03 | 1.08E-03 |
| BP | GO:0034504 | protein localization to nucleus | 8.30E-05 | 1.75E-03 | 1.08E-03 |
| BP | GO:0060541 | respiratory system development | 8.49E-05 | 1.77E-03 | 1.10E-03 |
| BP | GO:0010959 | regulation of metal ion transport | 8.98E-05 | 1.84E-03 | 1.14E-03 |
| BP | GO:0038034 | signal transduction in absence of ligand | 8.99E-05 | 1.84E-03 | 1.14E-03 |
| BP | GO:0097192 | extrinsic apoptotic signaling pathway in absence of ligand | 8.99E-05 | 1.84E-03 | 1.14E-03 |
| BP | GO:0051208 | sequestering of calcium ion | 9.32E-05 | 1.89E-03 | 1.17E-03 |
| BP | GO:0031639 | plasminogen activation | 9.41E-05 | 1.89E-03 | 1.17E-03 |
| BP | GO:0016051 | carbohydrate biosynthetic process | 9.97E-05 | 1.96E-03 | 1.21E-03 |
| BP | GO:0019932 | second-messenger-mediated signaling | 1.00E-04 | 1.96E-03 | 1.21E-03 |
| BP | GO:0009132 | nucleoside diphosphate metabolic process | 1.00E-04 | 1.96E-03 | 1.21E-03 |
| BP | GO:0035303 | regulation of dephosphorylation | 1.00E-04 | 1.96E-03 | 1.21E-03 |
| BP | GO:0060193 | positive regulation of lipase activity | 1.01E-04 | 1.96E-03 | 1.22E-03 |
| BP | GO:0050679 | positive regulation of epithelial cell proliferation | 1.05E-04 | 2.02E-03 | 1.25E-03 |
| BP | GO:1904385 | cellular response to angiotensin | 1.06E-04 | 2.03E-03 | 1.26E-03 |
| BP | GO:0030198 | extracellular matrix organization | 1.09E-04 | 2.07E-03 | 1.28E-03 |
| BP | GO:0043062 | extracellular structure organization | 1.11E-04 | 2.09E-03 | 1.30E-03 |
| BP | GO:0045229 | external encapsulating structure organization | 1.15E-04 | 2.16E-03 | 1.34E-03 |
| BP | GO:0010810 | regulation of cell-substrate adhesion | 1.22E-04 | 2.28E-03 | 1.41E-03 |
| BP | GO:0009166 | nucleotide catabolic process | 1.26E-04 | 2.34E-03 | 1.45E-03 |
| BP | GO:0051651 | maintenance of location in cell | 1.29E-04 | 2.36E-03 | 1.47E-03 |
| BP | GO:0005977 | glycogen metabolic process | 1.33E-04 | 2.41E-03 | 1.50E-03 |
| BP | GO:0048662 | negative regulation of smooth muscle cell proliferation | 1.33E-04 | 2.41E-03 | 1.50E-03 |
| BP | GO:0032102 | negative regulation of response to external stimulus | 1.38E-04 | 2.49E-03 | 1.54E-03 |
| BP | GO:0006073 | cellular glucan metabolic process | 1.41E-04 | 2.50E-03 | 1.55E-03 |
| BP | GO:0044042 | glucan metabolic process | 1.41E-04 | 2.50E-03 | 1.55E-03 |
| BP | GO:0050870 | positive regulation of T cell activation | 1.42E-04 | 2.51E-03 | 1.56E-03 |
| BP | GO:0015980 | energy derivation by oxidation of organic compounds | 1.43E-04 | 2.51E-03 | 1.56E-03 |
| BP | GO:0002068 | glandular epithelial cell development | 1.48E-04 | 2.55E-03 | 1.58E-03 |
| BP | GO:0003309 | type B pancreatic cell differentiation | 1.48E-04 | 2.55E-03 | 1.58E-03 |
| BP | GO:0043536 | positive regulation of blood vessel endothelial cell migration | 1.48E-04 | 2.55E-03 | 1.58E-03 |
| BP | GO:0014065 | phosphatidylinositol 3-kinase signaling | 1.62E-04 | 2.77E-03 | 1.72E-03 |
| BP | GO:1990776 | response to angiotensin | 1.64E-04 | 2.79E-03 | 1.73E-03 |
| BP | GO:0042445 | hormone metabolic process | 1.68E-04 | 2.83E-03 | 1.76E-03 |
| BP | GO:0032103 | positive regulation of response to external stimulus | 1.70E-04 | 2.83E-03 | 1.76E-03 |
| BP | GO:0048545 | response to steroid hormone | 1.70E-04 | 2.83E-03 | 1.76E-03 |
| BP | GO:0062013 | positive regulation of small molecule metabolic process | 1.73E-04 | 2.85E-03 | 1.77E-03 |
| BP | GO:0051235 | maintenance of location | 1.73E-04 | 2.85E-03 | 1.77E-03 |
| BP | GO:0014068 | positive regulation of phosphatidylinositol 3-kinase signaling | 1.81E-04 | 2.95E-03 | 1.83E-03 |
| BP | GO:0061844 | antimicrobial humoral immune response mediated by  antimicrobial peptide | 1.81E-04 | 2.95E-03 | 1.83E-03 |
| BP | GO:0007160 | cell-matrix adhesion | 1.89E-04 | 3.06E-03 | 1.90E-03 |
| BP | GO:0051279 | regulation of release of sequestered calcium ion into cytosol | 1.90E-04 | 3.06E-03 | 1.90E-03 |
| BP | GO:0007584 | response to nutrient | 1.96E-04 | 3.12E-03 | 1.93E-03 |
| BP | GO:0043535 | regulation of blood vessel endothelial cell migration | 1.96E-04 | 3.12E-03 | 1.93E-03 |
| BP | GO:0035883 | enteroendocrine cell differentiation | 1.99E-04 | 3.13E-03 | 1.94E-03 |
| BP | GO:0051385 | response to mineralocorticoid | 1.99E-04 | 3.13E-03 | 1.94E-03 |
| BP | GO:0036294 | cellular response to decreased oxygen levels | 2.02E-04 | 3.16E-03 | 1.96E-03 |
| BP | GO:0001933 | negative regulation of protein phosphorylation | 2.08E-04 | 3.21E-03 | 1.99E-03 |
| BP | GO:0009205 | purine ribonucleoside triphosphate metabolic process | 2.09E-04 | 3.21E-03 | 1.99E-03 |
| BP | GO:1901292 | nucleoside phosphate catabolic process | 2.09E-04 | 3.21E-03 | 1.99E-03 |
| BP | GO:0050863 | regulation of T cell activation | 2.11E-04 | 3.21E-03 | 1.99E-03 |
| BP | GO:0062012 | regulation of small molecule metabolic process | 2.11E-04 | 3.21E-03 | 1.99E-03 |
| BP | GO:0045834 | positive regulation of lipid metabolic process | 2.15E-04 | 3.25E-03 | 2.02E-03 |
| BP | GO:1903037 | regulation of leukocyte cell-cell adhesion | 2.19E-04 | 3.28E-03 | 2.03E-03 |
| BP | GO:0033273 | response to vitamin | 2.19E-04 | 3.28E-03 | 2.03E-03 |
| BP | GO:0045765 | regulation of angiogenesis | 2.23E-04 | 3.32E-03 | 2.05E-03 |
| BP | GO:1900182 | positive regulation of protein localization to nucleus | 2.29E-04 | 3.39E-03 | 2.10E-03 |
| BP | GO:1903039 | positive regulation of leukocyte cell-cell adhesion | 2.37E-04 | 3.48E-03 | 2.16E-03 |
| BP | GO:0034405 | response to fluid shear stress | 2.39E-04 | 3.50E-03 | 2.17E-03 |
| BP | GO:0031960 | response to corticosteroid | 2.42E-04 | 3.52E-03 | 2.18E-03 |
| BP | GO:0019439 | aromatic compound catabolic process | 2.43E-04 | 3.52E-03 | 2.18E-03 |
| BP | GO:1901342 | regulation of vasculature development | 2.48E-04 | 3.57E-03 | 2.21E-03 |
| BP | GO:0055074 | calcium ion homeostasis | 2.50E-04 | 3.58E-03 | 2.22E-03 |
| BP | GO:0006112 | energy reserve metabolic process | 2.51E-04 | 3.58E-03 | 2.22E-03 |
| BP | GO:0009144 | purine nucleoside triphosphate metabolic process | 2.63E-04 | 3.72E-03 | 2.30E-03 |
| BP | GO:0090257 | regulation of muscle system process | 2.64E-04 | 3.72E-03 | 2.30E-03 |
| BP | GO:0051052 | regulation of DNA metabolic process | 2.65E-04 | 3.72E-03 | 2.30E-03 |
| BP | GO:0009199 | ribonucleoside triphosphate metabolic process | 2.86E-04 | 4.00E-03 | 2.48E-03 |
| BP | GO:0001667 | ameboidal-type cell migration | 2.97E-04 | 4.10E-03 | 2.54E-03 |
| BP | GO:0060191 | regulation of lipase activity | 2.99E-04 | 4.10E-03 | 2.54E-03 |
| BP | GO:1901657 | glycosyl compound metabolic process | 2.99E-04 | 4.10E-03 | 2.54E-03 |
| BP | GO:0045927 | positive regulation of growth | 3.00E-04 | 4.10E-03 | 2.54E-03 |
| BP | GO:0035304 | regulation of protein dephosphorylation | 3.12E-04 | 4.21E-03 | 2.61E-03 |
| BP | GO:0097306 | cellular response to alcohol | 3.12E-04 | 4.21E-03 | 2.61E-03 |
| BP | GO:0016049 | cell growth | 3.14E-04 | 4.21E-03 | 2.61E-03 |
| BP | GO:0071453 | cellular response to oxygen levels | 3.14E-04 | 4.21E-03 | 2.61E-03 |
| BP | GO:0002065 | columnar/cuboidal epithelial cell differentiation | 3.25E-04 | 4.34E-03 | 2.69E-03 |
| BP | GO:0043410 | positive regulation of MAPK cascade | 3.45E-04 | 4.58E-03 | 2.84E-03 |
| BP | GO:0043029 | T cell homeostasis | 3.61E-04 | 4.77E-03 | 2.95E-03 |
| BP | GO:0051223 | regulation of protein transport | 3.64E-04 | 4.79E-03 | 2.97E-03 |
| BP | GO:0003073 | regulation of systemic arterial blood pressure | 3.68E-04 | 4.81E-03 | 2.98E-03 |
| BP | GO:0044275 | cellular carbohydrate catabolic process | 3.89E-04 | 5.07E-03 | 3.14E-03 |
| BP | GO:0051348 | negative regulation of transferase activity | 3.98E-04 | 5.14E-03 | 3.18E-03 |
| BP | GO:0044264 | cellular polysaccharide metabolic process | 3.98E-04 | 5.14E-03 | 3.18E-03 |
| BP | GO:0043534 | blood vessel endothelial cell migration | 4.00E-04 | 5.14E-03 | 3.18E-03 |
| BP | GO:0007159 | leukocyte cell-cell adhesion | 4.06E-04 | 5.19E-03 | 3.22E-03 |
| BP | GO:0042326 | negative regulation of phosphorylation | 4.13E-04 | 5.23E-03 | 3.24E-03 |
| BP | GO:0050678 | regulation of epithelial cell proliferation | 4.13E-04 | 5.23E-03 | 3.24E-03 |
| BP | GO:0009154 | purine ribonucleotide catabolic process | 4.19E-04 | 5.28E-03 | 3.27E-03 |
| BP | GO:0048015 | phosphatidylinositol-mediated signaling | 4.21E-04 | 5.29E-03 | 3.28E-03 |
| BP | GO:0050900 | leukocyte migration | 4.26E-04 | 5.32E-03 | 3.30E-03 |
| BP | GO:0001936 | regulation of endothelial cell proliferation | 4.32E-04 | 5.37E-03 | 3.33E-03 |
| BP | GO:0050777 | negative regulation of immune response | 4.43E-04 | 5.49E-03 | 3.40E-03 |
| BP | GO:1900371 | regulation of purine nucleotide biosynthetic process | 4.50E-04 | 5.54E-03 | 3.43E-03 |
| BP | GO:0048017 | inositol lipid-mediated signaling | 4.66E-04 | 5.72E-03 | 3.55E-03 |
| BP | GO:0043542 | endothelial cell migration | 4.73E-04 | 5.78E-03 | 3.58E-03 |
| BP | GO:0007219 | Notch signaling pathway | 4.78E-04 | 5.82E-03 | 3.60E-03 |
| BP | GO:0030808 | regulation of nucleotide biosynthetic process | 4.82E-04 | 5.84E-03 | 3.62E-03 |
| BP | GO:0010522 | regulation of calcium ion transport into cytosol | 5.00E-04 | 6.02E-03 | 3.73E-03 |
| BP | GO:0002040 | sprouting angiogenesis | 5.28E-04 | 6.34E-03 | 3.93E-03 |
| BP | GO:0051896 | regulation of protein kinase B signaling | 5.41E-04 | 6.46E-03 | 4.01E-03 |
| BP | GO:0006195 | purine nucleotide catabolic process | 5.52E-04 | 6.49E-03 | 4.02E-03 |
| BP | GO:0031295 | T cell costimulation | 5.52E-04 | 6.49E-03 | 4.02E-03 |
| BP | GO:1904646 | cellular response to amyloid-beta | 5.52E-04 | 6.49E-03 | 4.02E-03 |
| BP | GO:1904181 | positive regulation of membrane depolarization | 5.53E-04 | 6.49E-03 | 4.02E-03 |
| BP | GO:1901654 | response to ketone | 5.82E-04 | 6.80E-03 | 4.21E-03 |
| BP | GO:0034655 | nucleobase-containing compound catabolic process | 5.85E-04 | 6.81E-03 | 4.22E-03 |
| BP | GO:0031018 | endocrine pancreas development | 5.89E-04 | 6.82E-03 | 4.23E-03 |
| BP | GO:0022409 | positive regulation of cell-cell adhesion | 5.91E-04 | 6.82E-03 | 4.23E-03 |
| BP | GO:0046390 | ribose phosphate biosynthetic process | 5.96E-04 | 6.84E-03 | 4.24E-03 |
| BP | GO:0005976 | polysaccharide metabolic process | 5.98E-04 | 6.84E-03 | 4.24E-03 |
| BP | GO:0006164 | purine nucleotide biosynthetic process | 6.10E-04 | 6.95E-03 | 4.31E-03 |
| BP | GO:0051222 | positive regulation of protein transport | 6.12E-04 | 6.95E-03 | 4.31E-03 |
| BP | GO:0003254 | regulation of membrane depolarization | 6.27E-04 | 7.06E-03 | 4.38E-03 |
| BP | GO:0031294 | lymphocyte costimulation | 6.27E-04 | 7.06E-03 | 4.38E-03 |
| BP | GO:0001935 | endothelial cell proliferation | 6.39E-04 | 7.17E-03 | 4.44E-03 |
| BP | GO:0001938 | positive regulation of endothelial cell proliferation | 6.63E-04 | 7.33E-03 | 4.54E-03 |
| BP | GO:0009141 | nucleoside triphosphate metabolic process | 6.63E-04 | 7.33E-03 | 4.54E-03 |
| BP | GO:0014066 | regulation of phosphatidylinositol 3-kinase signaling | 6.63E-04 | 7.33E-03 | 4.54E-03 |
| BP | GO:0030195 | negative regulation of blood coagulation | 6.67E-04 | 7.33E-03 | 4.54E-03 |
| BP | GO:1903426 | regulation of reactive oxygen species biosynthetic process | 6.67E-04 | 7.33E-03 | 4.54E-03 |
| BP | GO:0009120 | deoxyribonucleoside metabolic process | 6.74E-04 | 7.35E-03 | 4.55E-03 |
| BP | GO:0071803 | positive regulation of podosome assembly | 6.74E-04 | 7.35E-03 | 4.55E-03 |
| BP | GO:1900047 | negative regulation of hemostasis | 7.09E-04 | 7.70E-03 | 4.77E-03 |
| BP | GO:0034284 | response to monosaccharide | 7.33E-04 | 7.91E-03 | 4.90E-03 |
| BP | GO:0002696 | positive regulation of leukocyte activation | 7.35E-04 | 7.91E-03 | 4.90E-03 |
| BP | GO:0009612 | response to mechanical stimulus | 7.50E-04 | 8.04E-03 | 4.98E-03 |
| BP | GO:1902041 | regulation of extrinsic apoptotic signaling pathway via death domain receptors | 7.52E-04 | 8.04E-03 | 4.98E-03 |
| BP | GO:0007162 | negative regulation of cell adhesion | 7.55E-04 | 8.04E-03 | 4.98E-03 |
| BP | GO:0006816 | calcium ion transport | 7.66E-04 | 8.10E-03 | 5.02E-03 |
| BP | GO:0019722 | calcium-mediated signaling | 7.67E-04 | 8.10E-03 | 5.02E-03 |
| BP | GO:0002683 | negative regulation of immune system process | 7.77E-04 | 8.17E-03 | 5.07E-03 |
| BP | GO:0071383 | cellular response to steroid hormone stimulus | 7.84E-04 | 8.22E-03 | 5.09E-03 |
| BP | GO:0009409 | response to cold | 7.97E-04 | 8.33E-03 | 5.16E-03 |
| BP | GO:1904951 | positive regulation of establishment of protein localization | 8.08E-04 | 8.40E-03 | 5.21E-03 |
| BP | GO:1904019 | epithelial cell apoptotic process | 8.33E-04 | 8.64E-03 | 5.35E-03 |
| BP | GO:0050819 | negative regulation of coagulation | 8.44E-04 | 8.68E-03 | 5.38E-03 |
| BP | GO:1904036 | negative regulation of epithelial cell apoptotic process | 8.44E-04 | 8.68E-03 | 5.38E-03 |
| BP | GO:0045471 | response to ethanol | 8.60E-04 | 8.78E-03 | 5.44E-03 |
| BP | GO:0051897 | positive regulation of protein kinase B signaling | 8.60E-04 | 8.78E-03 | 5.44E-03 |
| BP | GO:0006979 | response to oxidative stress | 8.66E-04 | 8.78E-03 | 5.44E-03 |
| BP | GO:0048608 | reproductive structure development | 8.66E-04 | 8.78E-03 | 5.44E-03 |
| BP | GO:0030100 | regulation of endocytosis | 8.75E-04 | 8.83E-03 | 5.47E-03 |
| BP | GO:0050867 | positive regulation of cell activation | 9.02E-04 | 9.04E-03 | 5.60E-03 |
| BP | GO:0061458 | reproductive system development | 9.02E-04 | 9.04E-03 | 5.60E-03 |
| BP | GO:0051209 | release of sequestered calcium ion into cytosol | 9.15E-04 | 9.14E-03 | 5.66E-03 |
| BP | GO:0006959 | humoral immune response | 9.22E-04 | 9.18E-03 | 5.69E-03 |
| BP | GO:0002067 | glandular epithelial cell differentiation | 9.42E-04 | 9.25E-03 | 5.74E-03 |
| BP | GO:1903202 | negative regulation of oxidative stress-induced cell death | 9.42E-04 | 9.25E-03 | 5.74E-03 |
| BP | GO:0019730 | antimicrobial humoral response | 9.44E-04 | 9.25E-03 | 5.74E-03 |
| BP | GO:0051283 | negative regulation of sequestering of calcium ion | 9.44E-04 | 9.25E-03 | 5.74E-03 |
| BP | GO:0006000 | fructose metabolic process | 9.52E-04 | 9.27E-03 | 5.75E-03 |
| BP | GO:0006457 | protein folding | 9.52E-04 | 9.27E-03 | 5.75E-03 |
| BP | GO:0046700 | heterocycle catabolic process | 9.89E-04 | 9.56E-03 | 5.93E-03 |
| BP | GO:0050673 | epithelial cell proliferation | 9.89E-04 | 9.56E-03 | 5.93E-03 |
| BP | GO:0051282 | regulation of sequestering of calcium ion | 1.00E-03 | 9.66E-03 | 5.99E-03 |
| BP | GO:0003012 | muscle system process | 1.07E-03 | 1.02E-02 | 6.34E-03 |
| BP | GO:0044270 | cellular nitrogen compound catabolic process | 1.07E-03 | 1.02E-02 | 6.34E-03 |
| BP | GO:0007163 | establishment or maintenance of cell polarity | 1.10E-03 | 1.03E-02 | 6.39E-03 |
| BP | GO:0006754 | ATP biosynthetic process | 1.10E-03 | 1.03E-02 | 6.39E-03 |
| BP | GO:0009158 | ribonucleoside monophosphate catabolic process | 1.11E-03 | 1.03E-02 | 6.39E-03 |
| BP | GO:0009159 | deoxyribonucleoside monophosphate catabolic process | 1.11E-03 | 1.03E-02 | 6.39E-03 |
| BP | GO:0035791 | platelet-derived growth factor receptor-beta signaling pathway | 1.11E-03 | 1.03E-02 | 6.39E-03 |
| BP | GO:0038166 | angiotensin-activated signaling pathway | 1.11E-03 | 1.03E-02 | 6.39E-03 |
| BP | GO:0070141 | response to UV-A | 1.11E-03 | 1.03E-02 | 6.39E-03 |
| BP | GO:0071801 | regulation of podosome assembly | 1.11E-03 | 1.03E-02 | 6.39E-03 |
| BP | GO:0007596 | blood coagulation | 1.15E-03 | 1.06E-02 | 6.57E-03 |
| BP | GO:0071241 | cellular response to inorganic substance | 1.15E-03 | 1.06E-02 | 6.57E-03 |
| BP | GO:0048010 | vascular endothelial growth factor receptor signaling pathway | 1.16E-03 | 1.07E-02 | 6.63E-03 |
| BP | GO:0006874 | cellular calcium ion homeostasis | 1.17E-03 | 1.07E-02 | 6.63E-03 |
| BP | GO:0022407 | regulation of cell-cell adhesion | 1.17E-03 | 1.07E-02 | 6.63E-03 |
| BP | GO:0030168 | platelet activation | 1.19E-03 | 1.09E-02 | 6.75E-03 |
| BP | GO:0009161 | ribonucleoside monophosphate metabolic process | 1.22E-03 | 1.10E-02 | 6.85E-03 |
| BP | GO:0043666 | regulation of phosphoprotein phosphatase activity | 1.22E-03 | 1.10E-02 | 6.85E-03 |
| BP | GO:0010595 | positive regulation of endothelial cell migration | 1.23E-03 | 1.11E-02 | 6.88E-03 |
| BP | GO:0060348 | bone development | 1.24E-03 | 1.12E-02 | 6.92E-03 |
| BP | GO:0008406 | gonad development | 1.27E-03 | 1.13E-02 | 6.99E-03 |
| BP | GO:0009743 | response to carbohydrate | 1.27E-03 | 1.13E-02 | 6.99E-03 |
| BP | GO:0050817 | coagulation | 1.27E-03 | 1.13E-02 | 6.99E-03 |
| BP | GO:0005980 | glycogen catabolic process | 1.27E-03 | 1.13E-02 | 7.00E-03 |
| BP | GO:0034380 | high-density lipoprotein particle assembly | 1.27E-03 | 1.13E-02 | 7.00E-03 |
| BP | GO:1903078 | positive regulation of protein localization to plasma membrane | 1.28E-03 | 1.13E-02 | 7.00E-03 |
| BP | GO:0007599 | hemostasis | 1.29E-03 | 1.14E-02 | 7.04E-03 |
| BP | GO:0003158 | endothelium development | 1.30E-03 | 1.14E-02 | 7.07E-03 |
| BP | GO:1903409 | reactive oxygen species biosynthetic process | 1.34E-03 | 1.17E-02 | 7.28E-03 |
| BP | GO:0010594 | regulation of endothelial cell migration | 1.37E-03 | 1.19E-02 | 7.39E-03 |
| BP | GO:0030879 | mammary gland development | 1.37E-03 | 1.19E-02 | 7.39E-03 |
| BP | GO:0045088 | regulation of innate immune response | 1.39E-03 | 1.20E-02 | 7.46E-03 |
| BP | GO:0045137 | development of primary sexual characteristics | 1.39E-03 | 1.20E-02 | 7.46E-03 |
| BP | GO:0009251 | glucan catabolic process | 1.45E-03 | 1.24E-02 | 7.70E-03 |
| BP | GO:0061684 | chaperone-mediated autophagy | 1.45E-03 | 1.24E-02 | 7.70E-03 |
| BP | GO:2001212 | regulation of vasculogenesis | 1.45E-03 | 1.24E-02 | 7.70E-03 |
| BP | GO:0051893 | regulation of focal adhesion assembly | 1.47E-03 | 1.25E-02 | 7.76E-03 |
| BP | GO:0090109 | regulation of cell-substrate junction assembly | 1.47E-03 | 1.25E-02 | 7.76E-03 |
| BP | GO:0001819 | positive regulation of cytokine production | 1.48E-03 | 1.25E-02 | 7.76E-03 |
| BP | GO:0009116 | nucleoside metabolic process | 1.54E-03 | 1.30E-02 | 8.05E-03 |
| BP | GO:0031638 | zymogen activation | 1.54E-03 | 1.30E-02 | 8.05E-03 |
| BP | GO:0038061 | NIK/NF-kappaB signaling | 1.61E-03 | 1.35E-02 | 8.34E-03 |
| BP | GO:0002260 | lymphocyte homeostasis | 1.61E-03 | 1.35E-02 | 8.34E-03 |
| BP | GO:0070613 | regulation of protein processing | 1.61E-03 | 1.35E-02 | 8.34E-03 |
| BP | GO:0044247 | cellular polysaccharide catabolic process | 1.64E-03 | 1.36E-02 | 8.42E-03 |
| BP | GO:0046827 | positive regulation of protein export from nucleus | 1.64E-03 | 1.36E-02 | 8.42E-03 |
| BP | GO:0086103 | G protein-coupled receptor signaling pathway involved in heart process | 1.64E-03 | 1.36E-02 | 8.42E-03 |
| BP | GO:0010812 | negative regulation of cell-substrate adhesion | 1.69E-03 | 1.38E-02 | 8.56E-03 |
| BP | GO:0030193 | regulation of blood coagulation | 1.69E-03 | 1.38E-02 | 8.56E-03 |
| BP | GO:0034605 | cellular response to heat | 1.69E-03 | 1.38E-02 | 8.56E-03 |
| BP | GO:0030010 | establishment of cell polarity | 1.70E-03 | 1.38E-02 | 8.57E-03 |
| BP | GO:0071456 | cellular response to hypoxia | 1.70E-03 | 1.38E-02 | 8.57E-03 |
| BP | GO:0046637 | regulation of alpha-beta T cell differentiation | 1.76E-03 | 1.43E-02 | 8.83E-03 |
| BP | GO:1904377 | positive regulation of protein localization to cell periphery | 1.76E-03 | 1.43E-02 | 8.83E-03 |
| BP | GO:0009206 | purine ribonucleoside triphosphate biosynthetic process | 1.84E-03 | 1.46E-02 | 9.06E-03 |
| BP | GO:0150116 | regulation of cell-substrate junction organization | 1.84E-03 | 1.46E-02 | 9.06E-03 |
| BP | GO:1900046 | regulation of hemostasis | 1.84E-03 | 1.46E-02 | 9.06E-03 |
| BP | GO:1903317 | regulation of protein maturation | 1.84E-03 | 1.46E-02 | 9.06E-03 |
| BP | GO:0071695 | anatomical structure maturation | 1.84E-03 | 1.46E-02 | 9.06E-03 |
| BP | GO:0009125 | nucleoside monophosphate catabolic process | 1.85E-03 | 1.46E-02 | 9.06E-03 |
| BP | GO:0046040 | IMP metabolic process | 1.85E-03 | 1.46E-02 | 9.06E-03 |
| BP | GO:0072503 | cellular divalent inorganic cation homeostasis | 1.85E-03 | 1.46E-02 | 9.06E-03 |
| BP | GO:0031589 | cell-substrate adhesion | 1.87E-03 | 1.47E-02 | 9.13E-03 |
| BP | GO:0034614 | cellular response to reactive oxygen species | 1.88E-03 | 1.48E-02 | 9.15E-03 |
| BP | GO:0009145 | purine nucleoside triphosphate biosynthetic process | 1.92E-03 | 1.50E-02 | 9.28E-03 |
| BP | GO:0009988 | cell-cell recognition | 1.92E-03 | 1.50E-02 | 9.28E-03 |
| BP | GO:0051098 | regulation of binding | 1.92E-03 | 1.50E-02 | 9.28E-03 |
| BP | GO:0042698 | ovulation cycle | 2.00E-03 | 1.55E-02 | 9.61E-03 |
| BP | GO:0045862 | positive regulation of proteolysis | 2.00E-03 | 1.55E-02 | 9.61E-03 |
| BP | GO:0031098 | stress-activated protein kinase signaling cascade | 2.04E-03 | 1.56E-02 | 9.66E-03 |
| BP | GO:0071560 | cellular response to transforming growth factor beta stimulus | 2.04E-03 | 1.56E-02 | 9.66E-03 |
| BP | GO:0051251 | positive regulation of lymphocyte activation | 2.06E-03 | 1.56E-02 | 9.66E-03 |
| BP | GO:0051346 | negative regulation of hydrolase activity | 2.06E-03 | 1.56E-02 | 9.66E-03 |
| BP | GO:0000272 | polysaccharide catabolic process | 2.06E-03 | 1.56E-02 | 9.66E-03 |
| BP | GO:0006144 | purine nucleobase metabolic process | 2.06E-03 | 1.56E-02 | 9.66E-03 |
| BP | GO:0010544 | negative regulation of platelet activation | 2.06E-03 | 1.56E-02 | 9.66E-03 |
| BP | GO:0071800 | podosome assembly | 2.06E-03 | 1.56E-02 | 9.66E-03 |
| BP | GO:0008360 | regulation of cell shape | 2.07E-03 | 1.56E-02 | 9.70E-03 |
| BP | GO:0050818 | regulation of coagulation | 2.08E-03 | 1.57E-02 | 9.72E-03 |
| BP | GO:0009165 | nucleotide biosynthetic process | 2.15E-03 | 1.62E-02 | 1.00E-02 |
| BP | GO:0033143 | regulation of intracellular steroid hormone receptor signaling pathway | 2.16E-03 | 1.62E-02 | 1.01E-02 |
| BP | GO:0071216 | cellular response to biotic stimulus | 2.19E-03 | 1.63E-02 | 1.01E-02 |
| BP | GO:0097553 | calcium ion transmembrane import into cytosol | 2.22E-03 | 1.65E-02 | 1.02E-02 |
| BP | GO:0003015 | heart process | 2.22E-03 | 1.65E-02 | 1.02E-02 |
| BP | GO:1901293 | nucleoside phosphate biosynthetic process | 2.22E-03 | 1.65E-02 | 1.02E-02 |
| BP | GO:0071559 | response to transforming growth factor beta | 2.26E-03 | 1.67E-02 | 1.03E-02 |
| BP | GO:1903362 | regulation of cellular protein catabolic process | 2.26E-03 | 1.67E-02 | 1.03E-02 |
| BP | GO:0010310 | regulation of hydrogen peroxide metabolic process | 2.28E-03 | 1.68E-02 | 1.04E-02 |
| BP | GO:1903169 | regulation of calcium ion transmembrane transport | 2.33E-03 | 1.70E-02 | 1.06E-02 |
| BP | GO:1903522 | regulation of blood circulation | 2.34E-03 | 1.70E-02 | 1.06E-02 |
| BP | GO:0009201 | ribonucleoside triphosphate biosynthetic process | 2.34E-03 | 1.70E-02 | 1.06E-02 |
| BP | GO:0035924 | cellular response to vascular endothelial growth factor stimulus | 2.34E-03 | 1.70E-02 | 1.06E-02 |
| BP | GO:0046434 | organophosphate catabolic process | 2.38E-03 | 1.73E-02 | 1.07E-02 |
| BP | GO:0070227 | lymphocyte apoptotic process | 2.43E-03 | 1.76E-02 | 1.09E-02 |
| BP | GO:0030522 | intracellular receptor signaling pathway | 2.50E-03 | 1.80E-02 | 1.12E-02 |
| BP | GO:0002253 | activation of immune response | 2.50E-03 | 1.80E-02 | 1.12E-02 |
| BP | GO:0003091 | renal water homeostasis | 2.52E-03 | 1.80E-02 | 1.12E-02 |
| BP | GO:0006590 | thyroid hormone generation | 2.52E-03 | 1.80E-02 | 1.12E-02 |
| BP | GO:0008652 | cellular amino acid biosynthetic process | 2.53E-03 | 1.80E-02 | 1.12E-02 |
| BP | GO:0061045 | negative regulation of wound healing | 2.53E-03 | 1.80E-02 | 1.12E-02 |
| BP | GO:0043297 | apical junction assembly | 2.62E-03 | 1.86E-02 | 1.15E-02 |
| BP | GO:0009123 | nucleoside monophosphate metabolic process | 2.72E-03 | 1.93E-02 | 1.19E-02 |
| BP | GO:0030307 | positive regulation of cell growth | 2.73E-03 | 1.93E-02 | 1.20E-02 |
| BP | GO:0035584 | calcium-mediated signaling using intracellular calcium source | 2.76E-03 | 1.94E-02 | 1.20E-02 |
| BP | GO:1903428 | positive regulation of reactive oxygen species biosynthetic process | 2.76E-03 | 1.94E-02 | 1.20E-02 |
| BP | GO:0034637 | cellular carbohydrate biosynthetic process | 2.82E-03 | 1.98E-02 | 1.23E-02 |
| BP | GO:1903034 | regulation of response to wounding | 2.92E-03 | 2.03E-02 | 1.26E-02 |
| BP | GO:0001570 | vasculogenesis | 2.92E-03 | 2.03E-02 | 1.26E-02 |
| BP | GO:1902117 | positive regulation of organelle assembly | 2.92E-03 | 2.03E-02 | 1.26E-02 |
| BP | GO:0002053 | positive regulation of mesenchymal cell proliferation | 3.02E-03 | 2.07E-02 | 1.28E-02 |
| BP | GO:0019430 | removal of superoxide radicals | 3.02E-03 | 2.07E-02 | 1.28E-02 |
| BP | GO:0046128 | purine ribonucleoside metabolic process | 3.02E-03 | 2.07E-02 | 1.28E-02 |
| BP | GO:2001169 | regulation of ATP biosynthetic process | 3.02E-03 | 2.07E-02 | 1.28E-02 |
| BP | GO:0010660 | regulation of muscle cell apoptotic process | 3.03E-03 | 2.07E-02 | 1.28E-02 |
| BP | GO:0010833 | telomere maintenance via telomere lengthening | 3.03E-03 | 2.07E-02 | 1.28E-02 |
| BP | GO:0031016 | pancreas development | 3.03E-03 | 2.07E-02 | 1.28E-02 |
| BP | GO:0019319 | hexose biosynthetic process | 3.13E-03 | 2.14E-02 | 1.33E-02 |
| BP | GO:0006937 | regulation of muscle contraction | 3.18E-03 | 2.16E-02 | 1.34E-02 |
| BP | GO:0009152 | purine ribonucleotide biosynthetic process | 3.18E-03 | 2.16E-02 | 1.34E-02 |
| BP | GO:0014910 | regulation of smooth muscle cell migration | 3.24E-03 | 2.17E-02 | 1.35E-02 |
| BP | GO:0048041 | focal adhesion assembly | 3.24E-03 | 2.17E-02 | 1.35E-02 |
| BP | GO:0048678 | response to axon injury | 3.24E-03 | 2.17E-02 | 1.35E-02 |
| BP | GO:0060402 | calcium ion transport into cytosol | 3.24E-03 | 2.17E-02 | 1.35E-02 |
| BP | GO:0007548 | sex differentiation | 3.27E-03 | 2.17E-02 | 1.35E-02 |
| BP | GO:0009162 | deoxyribonucleoside monophosphate metabolic process | 3.28E-03 | 2.17E-02 | 1.35E-02 |
| BP | GO:0035162 | embryonic hemopoiesis | 3.28E-03 | 2.17E-02 | 1.35E-02 |
| BP | GO:0036120 | cellular response to platelet-derived growth factor stimulus | 3.28E-03 | 2.17E-02 | 1.35E-02 |
| BP | GO:0042026 | protein refolding | 3.28E-03 | 2.17E-02 | 1.35E-02 |
| BP | GO:0042730 | fibrinolysis | 3.28E-03 | 2.17E-02 | 1.35E-02 |
| BP | GO:0010921 | regulation of phosphatase activity | 3.35E-03 | 2.21E-02 | 1.37E-02 |
| BP | GO:0051899 | membrane depolarization | 3.35E-03 | 2.21E-02 | 1.37E-02 |
| BP | GO:0048771 | tissue remodeling | 3.45E-03 | 2.26E-02 | 1.40E-02 |
| BP | GO:0008625 | extrinsic apoptotic signaling pathway via death domain receptors | 3.47E-03 | 2.26E-02 | 1.40E-02 |
| BP | GO:0009142 | nucleoside triphosphate biosynthetic process | 3.47E-03 | 2.26E-02 | 1.40E-02 |
| BP | GO:0010657 | muscle cell apoptotic process | 3.47E-03 | 2.26E-02 | 1.40E-02 |
| BP | GO:0046889 | positive regulation of lipid biosynthetic process | 3.47E-03 | 2.26E-02 | 1.40E-02 |
| BP | GO:0044403 | biological process involved in symbiotic interaction | 3.52E-03 | 2.27E-02 | 1.41E-02 |
| BP | GO:0009164 | nucleoside catabolic process | 3.56E-03 | 2.27E-02 | 1.41E-02 |
| BP | GO:0030810 | positive regulation of nucleotide biosynthetic process | 3.56E-03 | 2.27E-02 | 1.41E-02 |
| BP | GO:0036119 | response to platelet-derived growth factor | 3.56E-03 | 2.27E-02 | 1.41E-02 |
| BP | GO:0046033 | AMP metabolic process | 3.56E-03 | 2.27E-02 | 1.41E-02 |
| BP | GO:0071450 | cellular response to oxygen radical | 3.56E-03 | 2.27E-02 | 1.41E-02 |
| BP | GO:0071451 | cellular response to superoxide | 3.56E-03 | 2.27E-02 | 1.41E-02 |
| BP | GO:0086064 | cell communication by electrical coupling involved in cardiac conduction | 3.56E-03 | 2.27E-02 | 1.41E-02 |
| BP | GO:1900373 | positive regulation of purine nucleotide biosynthetic process | 3.56E-03 | 2.27E-02 | 1.41E-02 |
| BP | GO:0001558 | regulation of cell growth | 3.58E-03 | 2.27E-02 | 1.41E-02 |
| BP | GO:0046364 | monosaccharide biosynthetic process | 3.59E-03 | 2.27E-02 | 1.41E-02 |
| BP | GO:1900542 | regulation of purine nucleotide metabolic process | 3.59E-03 | 2.27E-02 | 1.41E-02 |
| BP | GO:0031349 | positive regulation of defense response | 3.68E-03 | 2.33E-02 | 1.44E-02 |
| BP | GO:1901216 | positive regulation of neuron death | 3.70E-03 | 2.33E-02 | 1.45E-02 |
| BP | GO:0006470 | protein dephosphorylation | 3.79E-03 | 2.38E-02 | 1.48E-02 |
| BP | GO:0006140 | regulation of nucleotide metabolic process | 3.82E-03 | 2.38E-02 | 1.48E-02 |
| BP | GO:0006972 | hyperosmotic response | 3.85E-03 | 2.38E-02 | 1.48E-02 |
| BP | GO:0010954 | positive regulation of protein processing | 3.85E-03 | 2.38E-02 | 1.48E-02 |
| BP | GO:0032515 | negative regulation of phosphoprotein phosphatase activity | 3.85E-03 | 2.38E-02 | 1.48E-02 |
| BP | GO:0034377 | plasma lipoprotein particle assembly | 3.85E-03 | 2.38E-02 | 1.48E-02 |
| BP | GO:0045822 | negative regulation of heart contraction | 3.85E-03 | 2.38E-02 | 1.48E-02 |
| BP | GO:0060314 | regulation of ryanodine-sensitive calcium-release channel activity | 3.85E-03 | 2.38E-02 | 1.48E-02 |
| BP | GO:0016311 | dephosphorylation | 3.89E-03 | 2.40E-02 | 1.49E-02 |
| BP | GO:0001776 | leukocyte homeostasis | 3.95E-03 | 2.43E-02 | 1.50E-02 |
| BP | GO:1903578 | regulation of ATP metabolic process | 3.95E-03 | 2.43E-02 | 1.50E-02 |
| BP | GO:0042391 | regulation of membrane potential | 4.02E-03 | 2.47E-02 | 1.53E-02 |
| BP | GO:0021700 | developmental maturation | 4.07E-03 | 2.49E-02 | 1.55E-02 |
| BP | GO:0008217 | regulation of blood pressure | 4.13E-03 | 2.51E-02 | 1.56E-02 |
| BP | GO:0048469 | cell maturation | 4.13E-03 | 2.51E-02 | 1.56E-02 |
| BP | GO:0042403 | thyroid hormone metabolic process | 4.15E-03 | 2.51E-02 | 1.56E-02 |
| BP | GO:1903523 | negative regulation of blood circulation | 4.15E-03 | 2.51E-02 | 1.56E-02 |
| BP | GO:1904357 | negative regulation of telomere maintenance via telomere lengthening | 4.15E-03 | 2.51E-02 | 1.56E-02 |
| BP | GO:0014909 | smooth muscle cell migration | 4.20E-03 | 2.53E-02 | 1.57E-02 |
| BP | GO:0050804 | modulation of chemical synaptic transmission | 4.21E-03 | 2.53E-02 | 1.57E-02 |
| BP | GO:0009260 | ribonucleotide biosynthetic process | 4.21E-03 | 2.53E-02 | 1.57E-02 |
| BP | GO:0099177 | regulation of trans-synaptic signaling | 4.26E-03 | 2.55E-02 | 1.58E-02 |
| BP | GO:0061138 | morphogenesis of a branching epithelium | 4.29E-03 | 2.57E-02 | 1.59E-02 |
| BP | GO:0048732 | gland development | 4.30E-03 | 2.57E-02 | 1.59E-02 |
| BP | GO:0031647 | regulation of protein stability | 4.31E-03 | 2.57E-02 | 1.59E-02 |
| BP | GO:0007044 | cell-substrate junction assembly | 4.33E-03 | 2.57E-02 | 1.59E-02 |
| BP | GO:1903035 | negative regulation of response to wounding | 4.33E-03 | 2.57E-02 | 1.59E-02 |
| BP | GO:0000303 | response to superoxide | 4.45E-03 | 2.61E-02 | 1.62E-02 |
| BP | GO:0033688 | regulation of osteoblast proliferation | 4.45E-03 | 2.61E-02 | 1.62E-02 |
| BP | GO:0042133 | neurotransmitter metabolic process | 4.45E-03 | 2.61E-02 | 1.62E-02 |
| BP | GO:0042278 | purine nucleoside metabolic process | 4.45E-03 | 2.61E-02 | 1.62E-02 |
| BP | GO:1903319 | positive regulation of protein maturation | 4.45E-03 | 2.61E-02 | 1.62E-02 |
| BP | GO:1903672 | positive regulation of sprouting angiogenesis | 4.45E-03 | 2.61E-02 | 1.62E-02 |
| BP | GO:0006575 | cellular modified amino acid metabolic process | 4.55E-03 | 2.65E-02 | 1.64E-02 |
| BP | GO:0031099 | regeneration | 4.55E-03 | 2.65E-02 | 1.64E-02 |
| BP | GO:0002042 | cell migration involved in sprouting angiogenesis | 4.60E-03 | 2.66E-02 | 1.65E-02 |
| BP | GO:0045582 | positive regulation of T cell differentiation | 4.60E-03 | 2.66E-02 | 1.65E-02 |
| BP | GO:1901655 | cellular response to ketone | 4.60E-03 | 2.66E-02 | 1.65E-02 |
| BP | GO:1904035 | regulation of epithelial cell apoptotic process | 4.60E-03 | 2.66E-02 | 1.65E-02 |
| BP | GO:0022604 | regulation of cell morphogenesis | 4.62E-03 | 2.67E-02 | 1.65E-02 |
| BP | GO:0009749 | response to glucose | 4.63E-03 | 2.67E-02 | 1.65E-02 |
| BP | GO:0016236 | macroautophagy | 4.69E-03 | 2.69E-02 | 1.67E-02 |
| BP | GO:0060401 | cytosolic calcium ion transport | 4.72E-03 | 2.70E-02 | 1.67E-02 |
| BP | GO:0061136 | regulation of proteasomal protein catabolic process | 4.72E-03 | 2.70E-02 | 1.67E-02 |
| BP | GO:0048661 | positive regulation of smooth muscle cell proliferation | 4.74E-03 | 2.70E-02 | 1.67E-02 |
| BP | GO:0000305 | response to oxygen radical | 4.77E-03 | 2.70E-02 | 1.67E-02 |
| BP | GO:0009264 | deoxyribonucleotide catabolic process | 4.77E-03 | 2.70E-02 | 1.67E-02 |
| BP | GO:0010464 | regulation of mesenchymal cell proliferation | 4.77E-03 | 2.70E-02 | 1.67E-02 |
| BP | GO:0032369 | negative regulation of lipid transport | 4.77E-03 | 2.70E-02 | 1.67E-02 |
| BP | GO:0065005 | protein-lipid complex assembly | 4.77E-03 | 2.70E-02 | 1.67E-02 |
| BP | GO:0009746 | response to hexose | 4.99E-03 | 2.81E-02 | 1.74E-02 |
| BP | GO:0008593 | regulation of Notch signaling pathway | 5.02E-03 | 2.82E-02 | 1.75E-02 |
| BP | GO:0043279 | response to alkaloid | 5.02E-03 | 2.82E-02 | 1.75E-02 |
| BP | GO:0006734 | NADH metabolic process | 5.10E-03 | 2.84E-02 | 1.76E-02 |
| BP | GO:0034656 | nucleobase-containing small molecule catabolic process | 5.10E-03 | 2.84E-02 | 1.76E-02 |
| BP | GO:0046386 | deoxyribose phosphate catabolic process | 5.10E-03 | 2.84E-02 | 1.76E-02 |
| BP | GO:1903579 | negative regulation of ATP metabolic process | 5.10E-03 | 2.84E-02 | 1.76E-02 |
| BP | GO:1901214 | regulation of neuron death | 5.15E-03 | 2.86E-02 | 1.77E-02 |
| BP | GO:0000079 | regulation of cyclin-dependent protein serine/threonine kinase activity | 5.17E-03 | 2.86E-02 | 1.77E-02 |
| BP | GO:0150115 | cell-substrate junction organization | 5.17E-03 | 2.86E-02 | 1.77E-02 |
| BP | GO:0043393 | regulation of protein binding | 5.26E-03 | 2.91E-02 | 1.80E-02 |
| BP | GO:0007015 | actin filament organization | 5.30E-03 | 2.92E-02 | 1.81E-02 |
| BP | GO:0005979 | regulation of glycogen biosynthetic process | 5.44E-03 | 2.96E-02 | 1.83E-02 |
| BP | GO:0010880 | regulation of release of sequestered calcium ion into cytosol by sarcoplasmic reticulum | 5.44E-03 | 2.96E-02 | 1.83E-02 |
| BP | GO:0010962 | regulation of glucan biosynthetic process | 5.44E-03 | 2.96E-02 | 1.83E-02 |
| BP | GO:0045737 | positive regulation of cyclin-dependent protein serine/threonine  kinase activity | 5.44E-03 | 2.96E-02 | 1.83E-02 |
| BP | GO:0046825 | regulation of protein export from nucleus | 5.44E-03 | 2.96E-02 | 1.83E-02 |
| BP | GO:0060055 | angiogenesis involved in wound healing | 5.44E-03 | 2.96E-02 | 1.83E-02 |
| BP | GO:1902042 | negative regulation of extrinsic apoptotic signaling pathway via death domain receptors | 5.44E-03 | 2.96E-02 | 1.83E-02 |
| BP | GO:0032872 | regulation of stress-activated MAPK cascade | 5.46E-03 | 2.96E-02 | 1.84E-02 |
| BP | GO:0001763 | morphogenesis of a branching structure | 5.55E-03 | 3.01E-02 | 1.86E-02 |
| BP | GO:0060249 | anatomical structure homeostasis | 5.58E-03 | 3.02E-02 | 1.87E-02 |
| BP | GO:0046620 | regulation of organ growth | 5.62E-03 | 3.03E-02 | 1.88E-02 |
| BP | GO:1904029 | regulation of cyclin-dependent protein kinase activity | 5.62E-03 | 3.03E-02 | 1.88E-02 |
| BP | GO:0007179 | transforming growth factor beta receptor signaling pathway | 5.65E-03 | 3.04E-02 | 1.88E-02 |
| BP | GO:0070302 | regulation of stress-activated protein kinase signaling cascade | 5.75E-03 | 3.08E-02 | 1.91E-02 |
| BP | GO:1901888 | regulation of cell junction assembly | 5.75E-03 | 3.08E-02 | 1.91E-02 |
| BP | GO:0010644 | cell communication by electrical coupling | 5.79E-03 | 3.09E-02 | 1.91E-02 |
| BP | GO:0051085 | chaperone cofactor-dependent protein refolding | 5.79E-03 | 3.09E-02 | 1.91E-02 |
| BP | GO:1902115 | regulation of organelle assembly | 5.85E-03 | 3.11E-02 | 1.93E-02 |
| BP | GO:1903076 | regulation of protein localization to plasma membrane | 5.94E-03 | 3.15E-02 | 1.95E-02 |
| BP | GO:1905477 | positive regulation of protein localization to membrane | 5.94E-03 | 3.15E-02 | 1.95E-02 |
| BP | GO:0000302 | response to reactive oxygen species | 5.95E-03 | 3.15E-02 | 1.95E-02 |
| BP | GO:0007204 | positive regulation of cytosolic calcium ion concentration | 6.03E-03 | 3.18E-02 | 1.97E-02 |
| BP | GO:0043281 | regulation of cysteine-type endopeptidase activity involved in apoptotic process | 6.06E-03 | 3.19E-02 | 1.98E-02 |
| BP | GO:0014812 | muscle cell migration | 6.10E-03 | 3.20E-02 | 1.99E-02 |
| BP | GO:0001893 | maternal placenta development | 6.15E-03 | 3.20E-02 | 1.99E-02 |
| BP | GO:0009112 | nucleobase metabolic process | 6.15E-03 | 3.20E-02 | 1.99E-02 |
| BP | GO:0033687 | osteoblast proliferation | 6.15E-03 | 3.20E-02 | 1.99E-02 |
| BP | GO:1901099 | negative regulation of signal transduction in absence of ligand | 6.15E-03 | 3.20E-02 | 1.99E-02 |
| BP | GO:2001240 | negative regulation of extrinsic apoptotic signaling pathway in absence of ligand | 6.15E-03 | 3.20E-02 | 1.99E-02 |
| BP | GO:0045216 | cell-cell junction organization | 6.16E-03 | 3.20E-02 | 1.99E-02 |
| BP | GO:0046634 | regulation of alpha-beta T cell activation | 6.26E-03 | 3.23E-02 | 2.00E-02 |
| BP | GO:0071868 | cellular response to monoamine stimulus | 6.26E-03 | 3.23E-02 | 2.00E-02 |
| BP | GO:0071870 | cellular response to catecholamine stimulus | 6.26E-03 | 3.23E-02 | 2.00E-02 |
| BP | GO:0060562 | epithelial tube morphogenesis | 6.26E-03 | 3.23E-02 | 2.00E-02 |
| BP | GO:0030593 | neutrophil chemotaxis | 6.43E-03 | 3.31E-02 | 2.05E-02 |
| BP | GO:0071902 | positive regulation of protein serine/threonine kinase activity | 6.48E-03 | 3.32E-02 | 2.06E-02 |
| BP | GO:0016486 | peptide hormone processing | 6.52E-03 | 3.32E-02 | 2.06E-02 |
| BP | GO:0035308 | negative regulation of protein dephosphorylation | 6.52E-03 | 3.32E-02 | 2.06E-02 |
| BP | GO:0043276 | anoikis | 6.52E-03 | 3.32E-02 | 2.06E-02 |
| BP | GO:1900181 | negative regulation of protein localization to nucleus | 6.52E-03 | 3.32E-02 | 2.06E-02 |
| BP | GO:2000352 | negative regulation of endothelial cell apoptotic process | 6.52E-03 | 3.32E-02 | 2.06E-02 |
| BP | GO:1901215 | negative regulation of neuron death | 6.59E-03 | 3.34E-02 | 2.07E-02 |
| BP | GO:0008637 | apoptotic mitochondrial changes | 6.59E-03 | 3.34E-02 | 2.07E-02 |
| BP | GO:0045621 | positive regulation of lymphocyte differentiation | 6.59E-03 | 3.34E-02 | 2.07E-02 |
| BP | GO:0032496 | response to lipopolysaccharide | 6.67E-03 | 3.37E-02 | 2.09E-02 |
| BP | GO:0017038 | protein import | 6.70E-03 | 3.38E-02 | 2.10E-02 |
| BP | GO:0007173 | epidermal growth factor receptor signaling pathway | 6.77E-03 | 3.40E-02 | 2.11E-02 |
| BP | GO:0071867 | response to monoamine | 6.77E-03 | 3.40E-02 | 2.11E-02 |
| BP | GO:0071869 | response to catecholamine | 6.77E-03 | 3.40E-02 | 2.11E-02 |
| BP | GO:0008016 | regulation of heart contraction | 6.81E-03 | 3.40E-02 | 2.11E-02 |
| BP | GO:0042180 | cellular ketone metabolic process | 6.81E-03 | 3.40E-02 | 2.11E-02 |
| BP | GO:0001569 | branching involved in blood vessel morphogenesis | 6.90E-03 | 3.40E-02 | 2.11E-02 |
| BP | GO:0014808 | release of sequestered calcium ion into cytosol by sarcoplasmic reticulum | 6.90E-03 | 3.40E-02 | 2.11E-02 |
| BP | GO:0032205 | negative regulation of telomere maintenance | 6.90E-03 | 3.40E-02 | 2.11E-02 |
| BP | GO:0033146 | regulation of intracellular estrogen receptor signaling pathway | 6.90E-03 | 3.40E-02 | 2.11E-02 |
| BP | GO:0044319 | wound healing, spreading of cells | 6.90E-03 | 3.40E-02 | 2.11E-02 |
| BP | GO:0090505 | epiboly involved in wound healing | 6.90E-03 | 3.40E-02 | 2.11E-02 |
| BP | GO:1901658 | glycosyl compound catabolic process | 6.90E-03 | 3.40E-02 | 2.11E-02 |
| BP | GO:1904031 | positive regulation of cyclin-dependent protein kinase activity | 6.90E-03 | 3.40E-02 | 2.11E-02 |
| BP | GO:0071887 | leukocyte apoptotic process | 6.94E-03 | 3.41E-02 | 2.12E-02 |
| BP | GO:0001505 | regulation of neurotransmitter levels | 7.04E-03 | 3.44E-02 | 2.13E-02 |
| BP | GO:0006469 | negative regulation of protein kinase activity | 7.04E-03 | 3.44E-02 | 2.13E-02 |
| BP | GO:0034764 | positive regulation of transmembrane transport | 7.04E-03 | 3.44E-02 | 2.13E-02 |
| BP | GO:0044706 | multi-multicellular organism process | 7.04E-03 | 3.44E-02 | 2.13E-02 |
| BP | GO:0019216 | regulation of lipid metabolic process | 7.17E-03 | 3.50E-02 | 2.17E-02 |
| BP | GO:0045777 | positive regulation of blood pressure | 7.29E-03 | 3.52E-02 | 2.18E-02 |
| BP | GO:0071312 | cellular response to alkaloid | 7.29E-03 | 3.52E-02 | 2.18E-02 |
| BP | GO:0090504 | epiboly | 7.29E-03 | 3.52E-02 | 2.18E-02 |
| BP | GO:0140448 | signaling receptor ligand precursor processing | 7.29E-03 | 3.52E-02 | 2.18E-02 |
| BP | GO:1903514 | release of sequestered calcium ion into cytosol by endoplasmic reticulum | 7.29E-03 | 3.52E-02 | 2.18E-02 |
| BP | GO:0046632 | alpha-beta T cell differentiation | 7.30E-03 | 3.52E-02 | 2.18E-02 |
| BP | GO:0050866 | negative regulation of cell activation | 7.39E-03 | 3.56E-02 | 2.21E-02 |
| BP | GO:0071222 | cellular response to lipopolysaccharide | 7.50E-03 | 3.61E-02 | 2.24E-02 |
| BP | GO:0002685 | regulation of leukocyte migration | 7.63E-03 | 3.63E-02 | 2.25E-02 |
| BP | GO:0019221 | cytokine-mediated signaling pathway | 7.63E-03 | 3.63E-02 | 2.25E-02 |
| BP | GO:0018958 | phenol-containing compound metabolic process | 7.66E-03 | 3.63E-02 | 2.25E-02 |
| BP | GO:0003203 | endocardial cushion morphogenesis | 7.69E-03 | 3.63E-02 | 2.25E-02 |
| BP | GO:0009119 | ribonucleoside metabolic process | 7.69E-03 | 3.63E-02 | 2.25E-02 |
| BP | GO:0010923 | negative regulation of phosphatase activity | 7.69E-03 | 3.63E-02 | 2.25E-02 |
| BP | GO:0048009 | insulin-like growth factor receptor signaling pathway | 7.69E-03 | 3.63E-02 | 2.25E-02 |
| BP | GO:0051084 | 'de novo' post-translational protein folding | 7.69E-03 | 3.63E-02 | 2.25E-02 |
| BP | GO:0070873 | regulation of glycogen metabolic process | 7.69E-03 | 3.63E-02 | 2.25E-02 |
| BP | GO:0090218 | positive regulation of lipid kinase activity | 7.69E-03 | 3.63E-02 | 2.25E-02 |
| BP | GO:0003014 | renal system process | 7.85E-03 | 3.70E-02 | 2.29E-02 |
| BP | GO:0030518 | intracellular steroid hormone receptor signaling pathway | 8.04E-03 | 3.78E-02 | 2.34E-02 |
| BP | GO:1990748 | cellular detoxification | 8.04E-03 | 3.78E-02 | 2.34E-02 |
| BP | GO:0006936 | muscle contraction | 8.08E-03 | 3.79E-02 | 2.35E-02 |
| BP | GO:0001990 | regulation of systemic arterial blood pressure by hormone | 8.10E-03 | 3.79E-02 | 2.35E-02 |
| BP | GO:0060416 | response to growth hormone | 8.10E-03 | 3.79E-02 | 2.35E-02 |
| BP | GO:0045861 | negative regulation of proteolysis | 8.18E-03 | 3.82E-02 | 2.37E-02 |
| BP | GO:0071675 | regulation of mononuclear cell migration | 8.23E-03 | 3.84E-02 | 2.38E-02 |
| BP | GO:1903050 | regulation of proteolysis involved in cellular protein catabolic process | 8.24E-03 | 3.84E-02 | 2.38E-02 |
| BP | GO:0002831 | regulation of response to biotic stimulus | 8.27E-03 | 3.84E-02 | 2.38E-02 |
| BP | GO:0010817 | regulation of hormone levels | 8.40E-03 | 3.89E-02 | 2.41E-02 |
| BP | GO:0045446 | endothelial cell differentiation | 8.43E-03 | 3.90E-02 | 2.42E-02 |
| BP | GO:0070997 | neuron death | 8.47E-03 | 3.91E-02 | 2.43E-02 |
| BP | GO:0032570 | response to progesterone | 8.52E-03 | 3.92E-02 | 2.43E-02 |
| BP | GO:0032885 | regulation of polysaccharide biosynthetic process | 8.52E-03 | 3.92E-02 | 2.43E-02 |
| BP | GO:0097242 | amyloid-beta clearance | 8.52E-03 | 3.92E-02 | 2.43E-02 |
| BP | GO:0002237 | response to molecule of bacterial origin | 8.56E-03 | 3.93E-02 | 2.44E-02 |
| BP | GO:0051480 | regulation of cytosolic calcium ion concentration | 8.76E-03 | 4.02E-02 | 2.49E-02 |
| BP | GO:0051928 | positive regulation of calcium ion transport | 8.83E-03 | 4.04E-02 | 2.50E-02 |
| BP | GO:0001953 | negative regulation of cell-matrix adhesion | 8.94E-03 | 4.06E-02 | 2.52E-02 |
| BP | GO:0006730 | one-carbon metabolic process | 8.94E-03 | 4.06E-02 | 2.52E-02 |
| BP | GO:0051281 | positive regulation of release of sequestered calcium ion into cytosol | 8.94E-03 | 4.06E-02 | 2.52E-02 |
| BP | GO:0071392 | cellular response to estradiol stimulus | 8.94E-03 | 4.06E-02 | 2.52E-02 |
| BP | GO:0090100 | positive regulation of transmembrane receptor protein serine/threonine kinase signaling pathway | 9.03E-03 | 4.08E-02 | 2.53E-02 |
| BP | GO:0071219 | cellular response to molecule of bacterial origin | 9.03E-03 | 4.08E-02 | 2.53E-02 |
| BP | GO:2000116 | regulation of cysteine-type endopeptidase activity | 9.03E-03 | 4.08E-02 | 2.53E-02 |
| BP | GO:0038127 | ERBB signaling pathway | 9.24E-03 | 4.17E-02 | 2.58E-02 |
| BP | GO:0006458 | 'de novo' protein folding | 9.38E-03 | 4.20E-02 | 2.60E-02 |
| BP | GO:0007339 | binding of sperm to zona pellucida | 9.38E-03 | 4.20E-02 | 2.60E-02 |
| BP | GO:0010463 | mesenchymal cell proliferation | 9.38E-03 | 4.20E-02 | 2.60E-02 |
| BP | GO:0033574 | response to testosterone | 9.38E-03 | 4.20E-02 | 2.60E-02 |
| BP | GO:0150077 | regulation of neuroinflammatory response | 9.38E-03 | 4.20E-02 | 2.60E-02 |
| BP | GO:0010906 | regulation of glucose metabolic process | 9.66E-03 | 4.30E-02 | 2.67E-02 |
| BP | GO:0022612 | gland morphogenesis | 9.66E-03 | 4.30E-02 | 2.67E-02 |
| BP | GO:0097237 | cellular response to toxic substance | 9.66E-03 | 4.30E-02 | 2.67E-02 |
| BP | GO:0009167 | purine ribonucleoside monophosphate metabolic process | 9.83E-03 | 4.31E-02 | 2.67E-02 |
| BP | GO:0010907 | positive regulation of glucose metabolic process | 9.83E-03 | 4.31E-02 | 2.67E-02 |
| BP | GO:0032467 | positive regulation of cytokinesis | 9.83E-03 | 4.31E-02 | 2.67E-02 |
| BP | GO:0045022 | early endosome to late endosome transport | 9.83E-03 | 4.31E-02 | 2.67E-02 |
| BP | GO:0050691 | regulation of defense response to virus by host | 9.83E-03 | 4.31E-02 | 2.67E-02 |
| BP | GO:0051602 | response to electrical stimulus | 9.83E-03 | 4.31E-02 | 2.67E-02 |
| BP | GO:0060338 | regulation of type I interferon-mediated signaling pathway | 9.83E-03 | 4.31E-02 | 2.67E-02 |
| BP | GO:0060412 | ventricular septum morphogenesis | 9.83E-03 | 4.31E-02 | 2.67E-02 |
| BP | GO:0070296 | sarcoplasmic reticulum calcium ion transport | 9.83E-03 | 4.31E-02 | 2.67E-02 |
| BP | GO:1904706 | negative regulation of vascular associated smooth muscle cell proliferation | 9.83E-03 | 4.31E-02 | 2.67E-02 |
| BP | GO:1904375 | regulation of protein localization to cell periphery | 9.87E-03 | 4.32E-02 | 2.68E-02 |
| BP | GO:0033673 | negative regulation of kinase activity | 1.00E-02 | 4.37E-02 | 2.71E-02 |
| BP | GO:0007178 | transmembrane receptor protein serine/threonine kinase  signaling pathway | 1.00E-02 | 4.37E-02 | 2.71E-02 |
| BP | GO:0009394 | 2'-deoxyribonucleotide metabolic process | 1.03E-02 | 4.47E-02 | 2.77E-02 |
| BP | GO:0071364 | cellular response to epidermal growth factor stimulus | 1.03E-02 | 4.47E-02 | 2.77E-02 |
| BP | GO:0046395 | carboxylic acid catabolic process | 1.03E-02 | 4.47E-02 | 2.77E-02 |
| BP | GO:0071900 | regulation of protein serine/threonine kinase activity | 1.05E-02 | 4.54E-02 | 2.81E-02 |
| BP | GO:0018108 | peptidyl-tyrosine phosphorylation | 1.06E-02 | 4.58E-02 | 2.84E-02 |
| BP | GO:0009126 | purine nucleoside monophosphate metabolic process | 1.07E-02 | 4.58E-02 | 2.84E-02 |
| BP | GO:0009262 | deoxyribonucleotide metabolic process | 1.07E-02 | 4.58E-02 | 2.84E-02 |
| BP | GO:0045981 | positive regulation of nucleotide metabolic process | 1.07E-02 | 4.58E-02 | 2.84E-02 |
| BP | GO:0048286 | lung alveolus development | 1.07E-02 | 4.58E-02 | 2.84E-02 |
| BP | GO:0060443 | mammary gland morphogenesis | 1.07E-02 | 4.58E-02 | 2.84E-02 |
| BP | GO:1900544 | positive regulation of purine nucleotide metabolic process | 1.07E-02 | 4.58E-02 | 2.84E-02 |
| BP | GO:1903573 | negative regulation of response to endoplasmic reticulum stress | 1.07E-02 | 4.58E-02 | 2.84E-02 |
| BP | GO:2001239 | regulation of extrinsic apoptotic signaling pathway in absence of ligand | 1.07E-02 | 4.58E-02 | 2.84E-02 |
| BP | GO:0050714 | positive regulation of protein secretion | 1.08E-02 | 4.58E-02 | 2.84E-02 |
| BP | GO:0071621 | granulocyte chemotaxis | 1.08E-02 | 4.58E-02 | 2.84E-02 |
| BP | GO:1990266 | neutrophil migration | 1.08E-02 | 4.58E-02 | 2.84E-02 |
| BP | GO:0016054 | organic acid catabolic process | 1.09E-02 | 4.63E-02 | 2.87E-02 |
| BP | GO:0018212 | peptidyl-tyrosine modification | 1.09E-02 | 4.63E-02 | 2.87E-02 |
| BP | GO:0042593 | glucose homeostasis | 1.12E-02 | 4.69E-02 | 2.91E-02 |
| BP | GO:0042476 | odontogenesis | 1.12E-02 | 4.69E-02 | 2.91E-02 |
| BP | GO:0006984 | ER-nucleus signaling pathway | 1.12E-02 | 4.69E-02 | 2.91E-02 |
| BP | GO:0016572 | histone phosphorylation | 1.12E-02 | 4.69E-02 | 2.91E-02 |
| BP | GO:0019692 | deoxyribose phosphate metabolic process | 1.12E-02 | 4.69E-02 | 2.91E-02 |
| BP | GO:0032881 | regulation of polysaccharide metabolic process | 1.12E-02 | 4.69E-02 | 2.91E-02 |
| BP | GO:0045933 | positive regulation of muscle contraction | 1.12E-02 | 4.69E-02 | 2.91E-02 |
| BP | GO:0071827 | plasma lipoprotein particle organization | 1.12E-02 | 4.69E-02 | 2.91E-02 |
| BP | GO:0098927 | vesicle-mediated transport between endosomal compartments | 1.12E-02 | 4.69E-02 | 2.91E-02 |
| BP | GO:0033500 | carbohydrate homeostasis | 1.14E-02 | 4.74E-02 | 2.94E-02 |
| BP | GO:0051403 | stress-activated MAPK cascade | 1.14E-02 | 4.74E-02 | 2.94E-02 |
| BP | GO:0060047 | heart contraction | 1.17E-02 | 4.84E-02 | 3.00E-02 |
| BP | GO:0000723 | telomere maintenance | 1.17E-02 | 4.84E-02 | 3.00E-02 |
| BP | GO:0035270 | endocrine system development | 1.17E-02 | 4.84E-02 | 3.00E-02 |
| BP | GO:0005978 | glycogen biosynthetic process | 1.17E-02 | 4.84E-02 | 3.00E-02 |
| BP | GO:0009250 | glucan biosynthetic process | 1.17E-02 | 4.84E-02 | 3.00E-02 |
| BP | GO:0070741 | response to interleukin-6 | 1.17E-02 | 4.84E-02 | 3.00E-02 |
| BP | GO:0061041 | regulation of wound healing | 1.19E-02 | 4.92E-02 | 3.05E-02 |
| BP | GO:0019079 | viral genome replication | 1.22E-02 | 4.96E-02 | 3.07E-02 |
| BP | GO:0043467 | regulation of generation of precursor metabolites and energy | 1.22E-02 | 4.96E-02 | 3.07E-02 |
| BP | GO:0003197 | endocardial cushion development | 1.22E-02 | 4.96E-02 | 3.07E-02 |
| BP | GO:0042220 | response to cocaine | 1.22E-02 | 4.96E-02 | 3.07E-02 |
| BP | GO:0045776 | negative regulation of blood pressure | 1.22E-02 | 4.96E-02 | 3.07E-02 |
| BP | GO:0050919 | negative chemotaxis | 1.22E-02 | 4.96E-02 | 3.07E-02 |
| BP | GO:0051972 | regulation of telomerase activity | 1.22E-02 | 4.96E-02 | 3.07E-02 |
| BP | GO:0070849 | response to epidermal growth factor | 1.22E-02 | 4.96E-02 | 3.07E-02 |
| BP | GO:1905953 | negative regulation of lipid localization | 1.22E-02 | 4.96E-02 | 3.07E-02 |
| BP | GO:2000378 | negative regulation of reactive oxygen species metabolic process | 1.22E-02 | 4.96E-02 | 3.07E-02 |
| CC | GO:0101002 | ficolin-1-rich granule | 3.20E-11 | 6.98E-09 | 4.58E-09 |
| CC | GO:1904813 | ficolin-1-rich granule lumen | 9.78E-09 | 1.07E-06 | 7.00E-07 |
| CC | GO:0034774 | secretory granule lumen | 1.35E-07 | 4.48E-06 | 2.94E-06 |
| CC | GO:0060205 | cytoplasmic vesicle lumen | 1.47E-07 | 4.48E-06 | 2.94E-06 |
| CC | GO:0045121 | membrane raft | 1.52E-07 | 4.48E-06 | 2.94E-06 |
| CC | GO:0031983 | vesicle lumen | 1.56E-07 | 4.48E-06 | 2.94E-06 |
| CC | GO:0098857 | membrane microdomain | 1.56E-07 | 4.48E-06 | 2.94E-06 |
| CC | GO:0005925 | focal adhesion | 1.64E-07 | 4.48E-06 | 2.94E-06 |
| CC | GO:0030055 | cell-substrate junction | 2.03E-07 | 4.93E-06 | 3.24E-06 |
| CC | GO:0042470 | melanosome | 1.95E-06 | 3.87E-05 | 2.54E-05 |
| CC | GO:0048770 | pigment granule | 1.95E-06 | 3.87E-05 | 2.54E-05 |
| CC | GO:0044853 | plasma membrane raft | 2.41E-06 | 4.38E-05 | 2.88E-05 |
| CC | GO:0005901 | caveola | 8.94E-06 | 1.50E-04 | 9.85E-05 |
| CC | GO:0070820 | tertiary granule | 2.05E-05 | 3.20E-04 | 2.10E-04 |
| CC | GO:0062023 | collagen-containing extracellular matrix | 1.04E-04 | 1.51E-03 | 9.95E-04 |
| CC | GO:0005775 | vacuolar lumen | 3.23E-04 | 4.37E-03 | 2.87E-03 |
| CC | GO:0043202 | lysosomal lumen | 3.41E-04 | 4.37E-03 | 2.87E-03 |
| CC | GO:0098562 | cytoplasmic side of membrane | 5.18E-04 | 6.28E-03 | 4.12E-03 |
| CC | GO:1904090 | peptidase inhibitor complex | 6.21E-04 | 7.08E-03 | 4.65E-03 |
| CC | GO:1902911 | protein kinase complex | 6.49E-04 | 7.08E-03 | 4.65E-03 |
| CC | GO:0101003 | ficolin-1-rich granule membrane | 1.19E-03 | 1.24E-02 | 8.14E-03 |
| CC | GO:0072562 | blood microparticle | 1.62E-03 | 1.60E-02 | 1.05E-02 |
| CC | GO:0042581 | specific granule | 2.20E-03 | 2.09E-02 | 1.37E-02 |
| CC | GO:0019897 | extrinsic component of plasma membrane | 2.74E-03 | 2.33E-02 | 1.53E-02 |
| CC | GO:0031430 | M band | 2.78E-03 | 2.33E-02 | 1.53E-02 |
| CC | GO:0071682 | endocytic vesicle lumen | 2.78E-03 | 2.33E-02 | 1.53E-02 |
| CC | GO:0031252 | cell leading edge | 2.96E-03 | 2.39E-02 | 1.57E-02 |
| CC | GO:0036019 | endolysosome | 4.11E-03 | 3.20E-02 | 2.10E-02 |
| CC | GO:0030667 | secretory granule membrane | 4.28E-03 | 3.22E-02 | 2.11E-02 |
| CC | GO:0009897 | external side of plasma membrane | 4.58E-03 | 3.23E-02 | 2.12E-02 |
| CC | GO:0031234 | extrinsic component of cytoplasmic side of plasma membrane | 4.74E-03 | 3.23E-02 | 2.12E-02 |
| CC | GO:1902554 | serine/threonine protein kinase complex | 4.74E-03 | 3.23E-02 | 2.12E-02 |
| CC | GO:0030027 | lamellipodium | 5.07E-03 | 3.35E-02 | 2.20E-02 |
| CC | GO:0031672 | A band | 6.02E-03 | 3.86E-02 | 2.53E-02 |
| CC | GO:0030139 | endocytic vesicle | 6.28E-03 | 3.86E-02 | 2.53E-02 |
| CC | GO:0031904 | endosome lumen | 6.37E-03 | 3.86E-02 | 2.53E-02 |
| MF | GO:0004252 | serine-type endopeptidase activity | 6.84E-10 | 2.00E-07 | 1.40E-07 |
| MF | GO:0008236 | serine-type peptidase activity | 1.69E-09 | 2.02E-07 | 1.42E-07 |
| MF | GO:0017171 | serine hydrolase activity | 2.07E-09 | 2.02E-07 | 1.42E-07 |
| MF | GO:0004175 | endopeptidase activity | 4.12E-07 | 3.02E-05 | 2.12E-05 |
| MF | GO:0030145 | manganese ion binding | 3.83E-06 | 2.24E-04 | 1.57E-04 |
| MF | GO:0045296 | cadherin binding | 2.72E-05 | 1.33E-03 | 9.29E-04 |
| MF | GO:0004712 | protein serine/threonine/tyrosine kinase activity | 3.26E-05 | 1.37E-03 | 9.57E-04 |
| MF | GO:0030246 | carbohydrate binding | 5.54E-05 | 2.03E-03 | 1.42E-03 |
| MF | GO:0005158 | insulin receptor binding | 6.75E-05 | 2.20E-03 | 1.54E-03 |
| MF | GO:0097110 | scaffold protein binding | 1.03E-04 | 3.03E-03 | 2.12E-03 |
| MF | GO:0023026 | MHC class II protein complex binding | 1.27E-04 | 3.37E-03 | 2.36E-03 |
| MF | GO:0023023 | MHC protein complex binding | 3.02E-04 | 7.37E-03 | 5.16E-03 |
| MF | GO:0008237 | metallopeptidase activity | 6.24E-04 | 1.41E-02 | 9.85E-03 |
| MF | GO:0004222 | metalloendopeptidase activity | 7.16E-04 | 1.46E-02 | 1.02E-02 |
| MF | GO:0031625 | ubiquitin protein ligase binding | 7.46E-04 | 1.46E-02 | 1.02E-02 |
| MF | GO:0004674 | protein serine/threonine kinase activity | 9.39E-04 | 1.58E-02 | 1.11E-02 |
| MF | GO:0050661 | NADP binding | 9.48E-04 | 1.58E-02 | 1.11E-02 |
| MF | GO:0051082 | unfolded protein binding | 9.89E-04 | 1.58E-02 | 1.11E-02 |
| MF | GO:0044389 | ubiquitin-like protein ligase binding | 1.03E-03 | 1.58E-02 | 1.11E-02 |
| MF | GO:0016004 | phospholipase activator activity | 1.15E-03 | 1.69E-02 | 1.18E-02 |
| MF | GO:0000287 | magnesium ion binding | 1.28E-03 | 1.79E-02 | 1.25E-02 |
| MF | GO:0060229 | lipase activator activity | 1.51E-03 | 2.02E-02 | 1.41E-02 |
| MF | GO:0005539 | glycosaminoglycan binding | 1.62E-03 | 2.06E-02 | 1.44E-02 |
| MF | GO:0005518 | collagen binding | 1.95E-03 | 2.30E-02 | 1.61E-02 |
| MF | GO:0106310 | protein serine kinase activity | 1.96E-03 | 2.30E-02 | 1.61E-02 |
| MF | GO:0016835 | carbon-oxygen lyase activity | 2.99E-03 | 3.17E-02 | 2.22E-02 |
| MF | GO:0019199 | transmembrane receptor protein kinase activity | 2.99E-03 | 3.17E-02 | 2.22E-02 |
| MF | GO:0016757 | glycosyltransferase activity | 3.06E-03 | 3.17E-02 | 2.22E-02 |
| MF | GO:0043274 | phospholipase binding | 3.14E-03 | 3.17E-02 | 2.22E-02 |
| MF | GO:0051117 | ATPase binding | 3.32E-03 | 3.23E-02 | 2.26E-02 |
| MF | GO:0019865 | immunoglobulin binding | 3.42E-03 | 3.23E-02 | 2.26E-02 |
| MF | GO:0050660 | flavin adenine dinucleotide binding | 3.68E-03 | 3.37E-02 | 2.36E-02 |
| MF | GO:0046875 | ephrin receptor binding | 4.64E-03 | 4.12E-02 | 2.89E-02 |
| MF | GO:0016829 | lyase activity | 5.56E-03 | 4.79E-02 | 3.36E-02 |
| MF | GO:0016758 | hexosyltransferase activity | 5.87E-03 | 4.91E-02 | 3.44E-02 |
| KEGG | hsa05215 | Prostate cancer | 2.49E-08 | 5.98E-06 | 4.12E-06 |
| KEGG | hsa05417 | Lipid and atherosclerosis | 3.02E-07 | 3.62E-05 | 2.49E-05 |
| KEGG | hsa04931 | Insulin resistance | 9.16E-07 | 7.32E-05 | 5.04E-05 |
| KEGG | hsa04520 | Adherens junction | 1.09E-05 | 6.56E-04 | 4.52E-04 |
| KEGG | hsa01230 | Biosynthesis of amino acids | 1.50E-05 | 7.22E-04 | 4.97E-04 |
| KEGG | hsa04910 | Insulin signaling pathway | 5.30E-05 | 1.74E-03 | 1.20E-03 |
| KEGG | hsa04915 | Estrogen signaling pathway | 5.55E-05 | 1.74E-03 | 1.20E-03 |
| KEGG | hsa05418 | Fluid shear stress and atherosclerosis | 5.81E-05 | 1.74E-03 | 1.20E-03 |
| KEGG | hsa04213 | Longevity regulating pathway - multiple species | 7.83E-05 | 2.08E-03 | 1.43E-03 |
| KEGG | hsa04914 | Progesterone-mediated oocyte maturation | 8.65E-05 | 2.08E-03 | 1.43E-03 |
| KEGG | hsa05205 | Proteoglycans in cancer | 1.02E-04 | 2.22E-03 | 1.53E-03 |
| KEGG | hsa00010 | Glycolysis / Gluconeogenesis | 1.14E-04 | 2.27E-03 | 1.57E-03 |
| KEGG | hsa04066 | HIF-1 signaling pathway | 1.25E-04 | 2.31E-03 | 1.59E-03 |
| KEGG | hsa01200 | Carbon metabolism | 1.68E-04 | 2.88E-03 | 1.98E-03 |
| KEGG | hsa04151 | PI3K-Akt signaling pathway | 1.94E-04 | 3.11E-03 | 2.14E-03 |
| KEGG | hsa01521 | EGFR tyrosine kinase inhibitor resistance | 2.48E-04 | 3.58E-03 | 2.47E-03 |
| KEGG | hsa04611 | Platelet activation | 2.54E-04 | 3.58E-03 | 2.47E-03 |
| KEGG | hsa04068 | FoxO signaling pathway | 3.42E-04 | 4.55E-03 | 3.14E-03 |
| KEGG | hsa04510 | Focal adhesion | 5.69E-04 | 7.19E-03 | 4.95E-03 |
| KEGG | hsa05213 | Endometrial cancer | 7.80E-04 | 9.37E-03 | 6.45E-03 |
| KEGG | hsa04370 | VEGF signaling pathway | 8.33E-04 | 9.52E-03 | 6.55E-03 |
| KEGG | hsa04660 | T cell receptor signaling pathway | 8.84E-04 | 9.64E-03 | 6.64E-03 |
| KEGG | hsa04922 | Glucagon signaling pathway | 1.00E-03 | 1.03E-02 | 7.06E-03 |
| KEGG | hsa05166 | Human T-cell leukemia virus 1 infection | 1.03E-03 | 1.03E-02 | 7.06E-03 |
| KEGG | hsa00051 | Fructose and mannose metabolism | 1.68E-03 | 1.61E-02 | 1.11E-02 |
| KEGG | hsa04919 | Thyroid hormone signaling pathway | 1.74E-03 | 1.61E-02 | 1.11E-02 |
| KEGG | hsa04926 | Relaxin signaling pathway | 2.31E-03 | 2.05E-02 | 1.41E-02 |
| KEGG | hsa04210 | Apoptosis | 2.90E-03 | 2.39E-02 | 1.65E-02 |
| KEGG | hsa05135 | Yersinia infection | 2.99E-03 | 2.39E-02 | 1.65E-02 |
| KEGG | hsa05162 | Measles | 3.19E-03 | 2.39E-02 | 1.65E-02 |
| KEGG | hsa04610 | Complement and coagulation cascades | 3.22E-03 | 2.39E-02 | 1.65E-02 |
| KEGG | hsa05210 | Colorectal cancer | 3.36E-03 | 2.39E-02 | 1.65E-02 |
| KEGG | hsa05203 | Viral carcinogenesis | 3.38E-03 | 2.39E-02 | 1.65E-02 |
| KEGG | hsa04140 | Autophagy - animal | 3.39E-03 | 2.39E-02 | 1.65E-02 |
| KEGG | hsa04211 | Longevity regulating pathway | 3.80E-03 | 2.61E-02 | 1.80E-02 |
| KEGG | hsa05226 | Gastric cancer | 4.29E-03 | 2.86E-02 | 1.97E-02 |
| KEGG | hsa04657 | IL-17 signaling pathway | 4.63E-03 | 3.00E-02 | 2.07E-02 |
| KEGG | hsa04150 | mTOR signaling pathway | 5.21E-03 | 3.29E-02 | 2.27E-02 |
| KEGG | hsa01522 | Endocrine resistance | 5.36E-03 | 3.30E-02 | 2.27E-02 |
| KEGG | hsa04217 | Necroptosis | 5.65E-03 | 3.34E-02 | 2.30E-02 |
| KEGG | hsa04933 | AGE-RAGE signaling pathway in diabetic complications | 5.76E-03 | 3.34E-02 | 2.30E-02 |
| KEGG | hsa00270 | Cysteine and methionine metabolism | 5.84E-03 | 3.34E-02 | 2.30E-02 |
| KEGG | hsa05161 | Hepatitis B | 6.11E-03 | 3.41E-02 | 2.35E-02 |
| KEGG | hsa04625 | C-type lectin receptor signaling pathway | 6.61E-03 | 3.59E-02 | 2.47E-02 |
| KEGG | hsa04014 | Ras signaling pathway | 6.73E-03 | 3.59E-02 | 2.47E-02 |
| KEGG | hsa04144 | Endocytosis | 9.18E-03 | 4.79E-02 | 3.30E-02 |

**Table 4** Molecular docking energy of target proteins with LVFP

| **Number** | **Gene Symbol** | **PDB ID** | **ligand** | **energy (****kcal/mol)** |
| --- | --- | --- | --- | --- |
| 1 | AKT1 | 1UNP | Rhamnose (25310) | -7.36 |
| 2 | VEGFR2 | 3WZE | Rhamnose (25310) | -8.65 |
| 3 | HSP90AA1 | 5NJX | Rhamnose (25310) | -6.47 |
| 4 | PKM | 7R6Y | Rhamnose (25310) | -5.74 |
| 5 | HSP90AA1 | 5NJX | Glucose (5793) | -5.57 |
| 6 | RHOA | 1UIX | Glucose (5793) | -3.91 |
| 7 | ENO1 | 7V67 | Glucose (5793) | -5.4 |
| 8 | GSK3β | 2O5k | Galactose (6036) | -7.68 |
| 9 | RHOA | 1UIX | Galactose (6036) | -4.11 |
| 10 | HSP90AA1 | 5NJX | Galactose (6036) | -5.92 |
| 11 | ENO1 | 7V67 | Galactose (6036) | -5.37 |
| 12 | SRC | 1NZL | Arabinose (4319195) | -6.45 |
| 13 | HSP90AA1 | 5NJX | Arabinose (4319195) | -5.58 |
| 14 | ENO1 | 7V67 | Arabinose (4319195) | -4.5 |
| 15 | IL-2 | 7DR4 | Arabinose (4319195) | -2.84 |
| 19 | RHOA | 1UIX | Galactose (6036) | -3.94 |


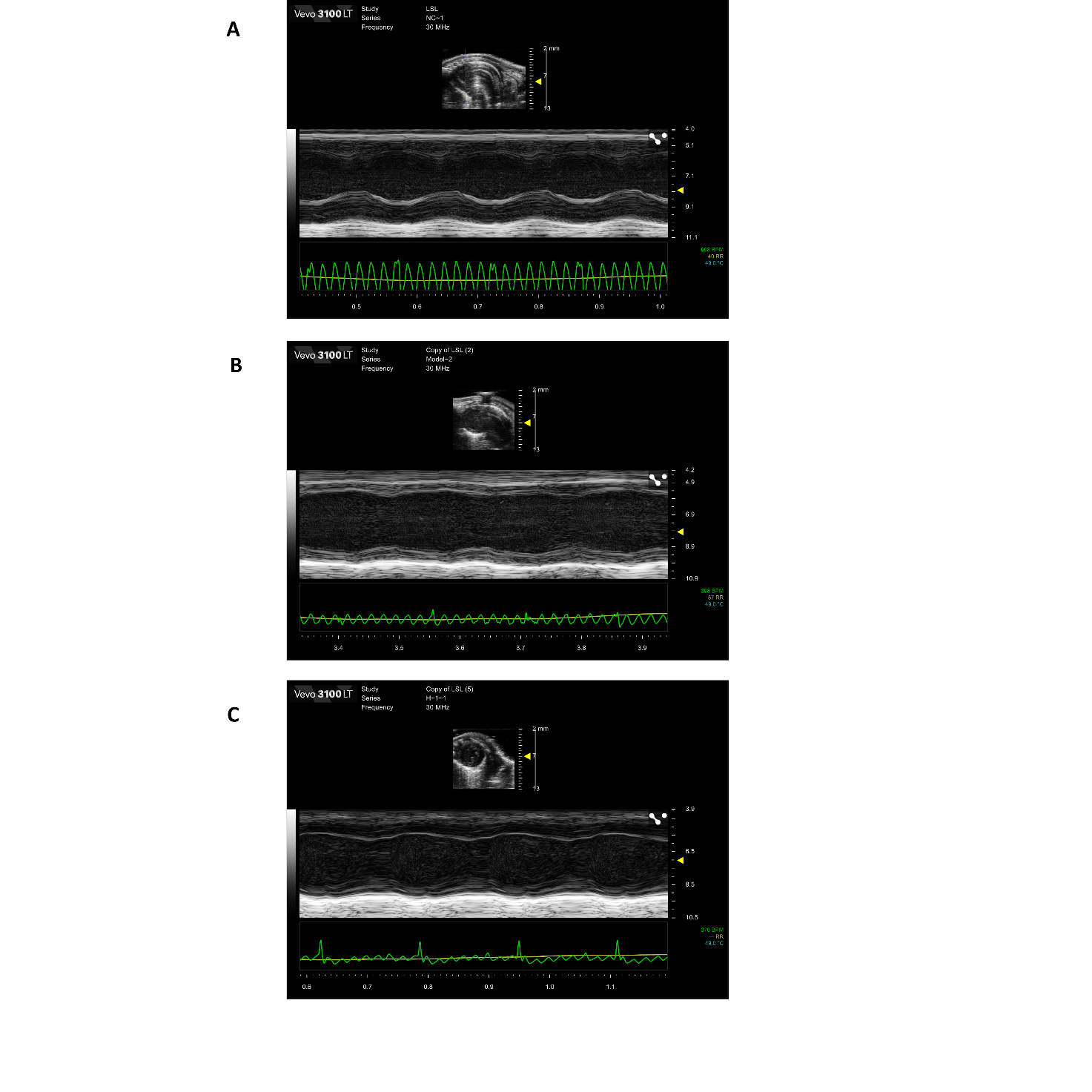


**Supplementary Figure 1** The original echocardiogram displays three conditions: (**A**) control, (**B**) ISO and (**C**) ISO+LVFP. Echo images with a resolution of 600dpi are end‐diastolic frames from the short‐axis view.


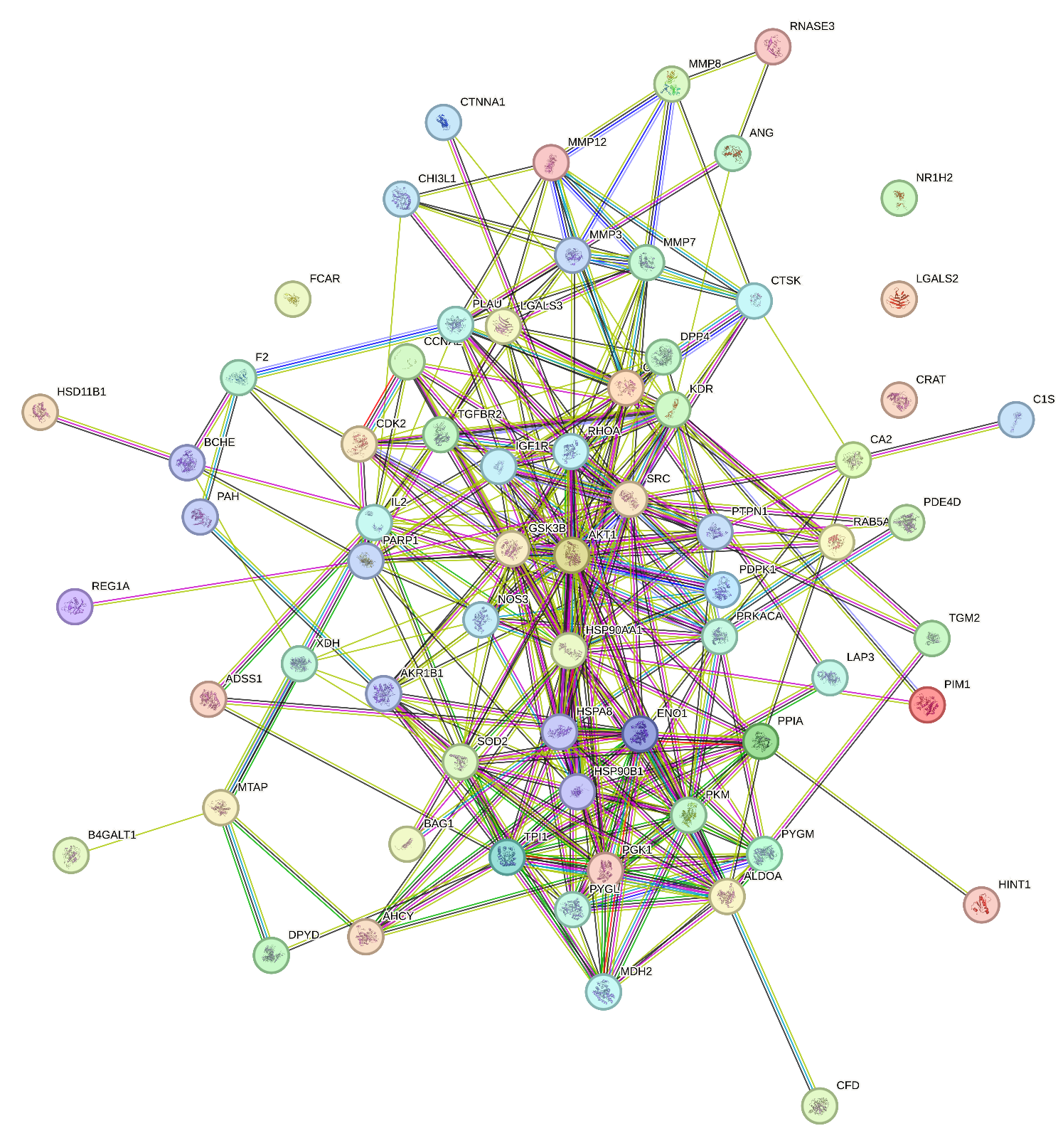


## Supplementary Figure 2 A Protein-protein interaction (PPI) network analysis was generated using String (Version 12.0) to evaluate 67 therapeutic targets involved in LVFP's treatment of MF.
